# Supplementary material for: Development, piloting and evaluation of an app-supported psychosocial prevention intervention to strengthen participation in working life: a study protocol of a mixed-methods approach
Source: BMJ Open. 2024 Feb 17;14(2):e081390. doi: 10.1136/bmjopen-2023-081390 (PMC10875476; doi:10.1136/bmjopen-2023-081390)
Supplement: Supplementary data [file bmjopen-2023-081390supp001.pdf]

Information on the validity and reliability of the assessment instruments

Instrument full name	Measurement	Number of items	Instrument abbreviation	Validity and reliability	Source
**Depression Anxiety Stress Scale-21**	depression, anxiety, stress	21	DASS-21	The participants in the total sample (n=307) can be divided into several groups based on their diagnosis and a group of people from the general population.<br>Panic disorder with or without agoraphobia: n=67, obsessive-compulsive disorder: n=54, social phobia: n=74, specific phobia: n=17, major depressive disorder: n=46, and non-clinical volunteers: n=49.<br>Internal consistency values for each subscale: depression α=0.94, anxiety α=0.87, and stress α=0.91.	[73]
			DASS-21 (German Version)	German Version of the DASS-21. Participants of the total sample size (n=714) was divided into two groups:<br>Pain patients: n=301, adults in the general population: n=413.<br>Internal consistency values for each subscale: depression α=0.88, anxiety α=0.76-0.80, stress α=0.86-0.87.	[74]
**eHealth Literacy Scale**	ability to find, evaluate, and apply health information from the internet	8	eHEALS	The total sample size comprised 664 participants aged 13–21 years.<br>The internal consistency of the overall scale is α=0.88.	[77]
			eHEALS	The total sample (n=866) consisted of people aged 50 years and over who were recruited for the Bone Power Study via two online portals.<br>The internal consistency of the overall scale is α=0.94.	[76]
			G-eHEALS (German Version)	This is a version of the eHEALS that has been translated into German. The sample (n=327) consisted of German students.<br>Reliability was tested using a 2-factor model, which resulted in α=0.88 for the information search dimension and α=0.82 for the information evaluation dimension.	[78]

Information on the validity and reliability of the assessment instruments

Instrument full name	Measurement	Number of items	Instrument abbreviation	Validity and reliability	Source
**Self-efficacy-optimism-pessimism questionnaire**	self-efficacy, optimism, pessimism	9	SWOP-K9	The SWOP-K9 consists of two questionnaires that were combined and shortened [82]. These are the self-efficacy questionnaire [79] and the optimism questionnaire [80].<br>The resulting SWOP-K9 was compared with the original questionnaires in five different clinical samples with n=726 inpatients. Statistical validation revealed good to optimal scores [81].<br>The reliability of the SWOP-K9 is based on the testing of the individual questionnaires on self-efficacy with an internal consistency of α=0.86 (n=208) and on optimism with an internal consistency of α=0.76 (n=624). According to the creators, the questionnaire is valid for adults.	[79-82]
**WHO Quality of Life Instrument, Short Form**	physical health, psychological health, social relationships, environment	26	WHOQOL-BREF	The total sample (n=11830) included individuals from 23 countries. Of these, n=2308 people from Germany participated. Participants were adults recruited from a variety of inpatient and outpatient healthcare facilities as well as from the general population.<br>For the German-speaking sample, the internal consistency for the subdomains was as follows: physical health α=0.88, mental health α=0.83, social relationships α=0.76, and environment α=0.78.	[71]
**Work Ability Index**	Ability to work, physical and mental health, job require-ment and job satisfaction	7	WAI	The sample size (n=3968) comprises people employed in Germany.<br>The internal consistency of the scale was α= 0.75.	[70]

α = Cronbach's alpha
