# Supplementary material for: Development, piloting and evaluation of an app-supported psychosocial prevention intervention to strengthen participation in working life: a study protocol of a mixed-methods approach
Source: BMJ Open. 2024 Feb 17;14(2):e081390. doi: 10.1136/bmjopen-2023-081390 (PMC10875476; doi:10.1136/bmjopen-2023-081390)
Supplement: Supplementary data [file bmjopen-2023-081390supp002.pdf]

Probandeninformation und Einwilligungserklärung zum Forschungsvorhaben: Erprobung und Evaluierung einer psychosozialen Präventionsleistung im Rahmen des Projektes PE³PP - Fokusgruppeninterviews

Probandeninformation

Sehr geehrte Teilnehmerin, sehr geehrter Teilnehmer,

wir bitten Sie, an einer wissenschaftlichen Studie teilzunehmen. In dieser Probandeninformation finden Sie alles Wesentliche zu der Studie.

Bitte lesen Sie diese Information sorgfältig durch. Wir werden zusätzlich mit Ihnen über die Studie sprechen und Ihre Fragen beantworten.

Pro Fokusgruppe werden 7-10 Teilnehmer:innen eingeschlossen. Insgesamt sollen über den Projektverlauf zwölf Fokusgruppen durchgeführt werden, sodass sich eine Teilnehmendenzahl von insgesamt 84 bis zu 120 Teilnehmer:innen ergibt.

Diese Studie wird vom Lehrstuhl für Soziale Determinanten der Gesundheit, Fakultät für Sport- und Gesundheitswissenschaften an der Technischen Universität München geplant und durchgeführt.

Sie wird durch öffentliche Mittel (Bundesministerium für Arbeit und Soziales) gefördert.

Die Studie wurde der zuständigen Ethikkommission vorgelegt. Sie hat keine Einwände erhoben.

Die Teilnahme an der Studie ist freiwillig. Wenn Sie nicht teilnehmen wollen oder wenn Sie später Ihre Einwilligung widerrufen, werden Ihnen daraus keine Nachteile entstehen.

Wenn Sie jetzt oder auch später weitere Fragen zur Studie haben, können Sie uns gerne kontaktieren. Wenden Sie sich dazu an Johannes Stephan, Georg-Brauchle-Ring 60/62, 80992 München, johannes.stephan2@tum.de.

Warum wird diese Studie durchgeführt?

Die Präventionsleistung RV Fit Psychische Gesundheit, an der Sie teilnehmen, ist ein neues Angebot der Deutschen Rentenversicherung Mitteldeutschland. Da es ein neues Angebot ist, soll untersucht werden, wie die Präventionsleistung aus Sicht der Teilnehmenden, also aus Ihrer Sicht, umgesetzt werden sollte. Es sollen Erkenntnisse darüber gewonnen werden, welche Erwartungen und Erfahrungen Sie bezüglich der Präventionsleistung haben. Ein weiteres Ziel der Fokusgruppeninterviews ist es, Punkte, durch die die Präventionsleistung verbessert werden kann, zu identifizieren. Die Ergebnisse dieser Untersuchung sollen dann im konkreten dazu genutzt werden die Präventionsleistung stetig zu verbessern und auf Basis Ihrer Erwartungen und Erfahrungen die Präventionsleistung auch für zukünftige Teilnehmer:inenn von RV Fit Psychische Gesundheit optimal gestalten zu können.

Wie läuft die Studie ab?

Zu Beginn des vereinbarten Termins geben wir Ihnen die Möglichkeit, aufkommende Fragen und Anmerkungen hinsichtlich der Einwilligungserklärung zu stellen. Nachdem Sie informiert

Stephan J, *et al. BMJ Open* 2024; 14:e081390. doi: 10.1136/bmjopen-2023-081390

wurden, bitten wir Sie die Einwilligungserklärung zu unterschreiben. Die unterschriebene Einwilligungserklärung können Sie dann entweder unmittelbar vor Ort, im Nachgang via E-Mail oder auch postalisch an die Forschenden übersenden. Die Fokusgruppen basieren auf einem wissenschaftlich konzipierten Leitfaden inklusive eines Erzählanreizes, der dazu dient den Austausch in der Gruppe zu initiieren. Die Fokusgruppe dauert zwischen 60 und 90 Minuten und wird aufgezeichnet. Die Fokusgruppe findet entweder in Präsenz vor Ort in den Kliniken oder online mittels einer Videokonferenz statt. Bei den Präsenzfokusgruppen erfolgt die Aufnahme mittels zweier im Raum verteilter Aufnahmegeräten. Die Präsenzfokusgruppen finden in entsprechenden passenden und geschützten Räumen in den Örtlichkeiten der Kliniken vor Ort statt. Die Onlinefokusgruppen finden über das Videokonferenztool Zoom statt. Die Tonaufnahme wird dabei anhand eines zusätzlich in das Zoom Meeting eingeloggten Computers sichergestellt, auf welchem über ein Aufnahmeprogramm aufgezeichnet wird.

Gibt es einen persönlichen Nutzen durch die Teilnahme an der Studie?

Durch die Teilnahme an den Fokusgruppeninterviews können Sie ihre Erfahrungen und Sichtweisen auf die absolvierte Präventionsleistung teilen und damit die Gestaltung und den Ablauf der Präventionsleistung mitgestalten. Ihre Perspektiven werden als wissenschaftliche fundiertes Feedback auch in das Projektteam rückgespiegelt.

Weiterhin ist es allerdings möglich, dass Sie durch Ihre Teilnahme keinen direkten Nutzen haben. Die Ergebnisse der Studie können jedoch in Zukunft anderen Menschen helfen.

Welche Risiken sind mit einer Teilnahme an der Studie verbunden?

Die Teilnahme an der Studie ist mit keinen Risiken verbunden.

Entstehen zusätzliche Kosten?

Durch die Teilnahme an der Studie entstehen Ihnen keine Kosten.

Was wird von mir erwartet?

Für die Teilnahme benötigen Sie nichts Weiteres. Alle notwendigen Materialien werden zur Verfügung gestellt. Die einzige Erwartung an Sie ist, dass Sie ihre Perspektive zu den verhandelten Themen kundtun.

Ist es möglich, im Verlauf aus der Studie auszuscheiden?

Ihre Teilnahme an dieser Studie ist freiwillig. Sie können jederzeit die Teilnahme beenden. Sie müssen dies nicht begründen. Es entstehen für Sie dadurch auch keine Nachteile.

Information zum Datenschutz

In dieser Studie ist der Lehrstuhl für Soziale Determinanten der Gesundheit für die Datenverarbeitung verantwortlich. Rechtsgrundlage für die Verarbeitung ist die persönliche

Einwilligung (Art. 6 Abs. 1a, Art. 9 Abs. 2a DSGVO). Die Daten werden zu jeder Zeit vertraulich behandelt.

Die Daten werden ausschließlich zum Zweck dieser oben beschriebenen Studie erhoben und nur in diesem Rahmen verwendet.

Die erfassten Daten umfassen auch personenbezogene Informationen, wie Ihre Stimme in der Tonbandaufnahme. Im nächsten Schritt wird die Tonbandaufnahme wortwörtlich niedergeschrieben, und danach wird die Aufnahme gelöscht. Alle Daten, durch die Sie unmittelbar identifiziert werden könnten, z.B. Ihr Name oder Ihr Geburtsdatum, werden durch einen Identifizierungscode ersetzt (pseudonymisiert). Damit ist es Unbefugten fast unmöglich, Sie zu identifizieren.

Die Daten werden an der Technische Universität München, Fakultät Sport- und Gesundheitswissenschaften, Lehrstuhl für Soziale Determinanten der Gesundheit gespeichert.

Wir bewahren die personenbezogenen Daten nur solange auf, wie dies für den oben genannten Zweck erforderlich ist. Die Daten werden spätestens nach Ablauf von 10 Jahre nach Abbruch oder Beendigung der Studie gelöscht.

Für die Studie führen wir eine Fokusgruppe mit Ihnen durch. Die Fokusgruppe vor Ort in Klinken wird mit Aufnahmegeräten aufgezeichnet. Die Fokusgruppen, welche während der Trainingsphase online via Videokonferenz durchgeführt werden, werden mittels eines zusätzlich eingeloggten Computers, auf welchem ein Aufnahmeprogramm läuft, aufgezeichnet. Die Tonaufnahme der Fokusgruppe wird zunächst an der Fakultät für Sport- und Gesundheitswissenschaften gespeichert. Das Gespräch wird innerhalb von sechs Monaten wortwörtlich aufgeschrieben (transkribiert). Die Tonaufnahmen werden dann gelöscht, sodass nur noch diese Abschrift des Gesprächs existiert. Alle Informationen, die dritten Personen Rückschluss auf Sie ermöglichen würden, werden bei der Abschrift so verändert, dass ein Rückschluss nicht mehr möglich ist. Der pseudonymisierte, aufgeschriebene Text wird für 10 Jahre in der Fakultät für Sport- und Gesundheitswissenschaften gespeichert und dann gelöscht. Wir übermitteln die personenbezogenen Daten weder an andere Einrichtungen in Deutschland, der EU, noch an ein Drittland außerhalb der EU oder an eine internationale Organisation.

Die Einwilligung zur Verarbeitung Ihrer Daten ist freiwillig. Sie können jederzeit die Einwilligung ohne Angabe von Gründen und ohne Nachteile für Sie widerrufen. Danach werden keine Daten mehr erhoben.

Sie können im Fall des Widerrufs die Löschung der erhobenen Daten verlangen. Die Daten können auch in anonymisierter Form weiterverwendet werden, wenn Sie dem zum Zeitpunkt Ihres Widerrufs zustimmen.

Sie haben das Recht, Auskunft über die Daten zu erhalten, auch in Form einer unentgeltlichen Kopie. Darüber hinaus können Sie die Berichtigung, Sperrung, Einschränkung der Verarbeitung oder Löschung sowie gegebenenfalls eine Übertragung der Daten verlangen. Die Rechtmäßigkeit der aufgrund der Einwilligung bis zum Widerruf erfolgten Verarbeitung wird hiervon nicht berührt.

Wenden Sie sich in diesen Fällen an:

Stephan J, *et al. BMJ Open* 2024; 14:e081390. doi: 10.1136/bmjopen-2023-081390

Prof. Dr. Matthias Richter
Lehrstuhlinhaber - Soziale Determinanten der Gesundheit
Georg-Brauchle-Ring 60/62
80992 München
Tel.: +49 89 289 24190
richter.matthias@tum.de

Bei Rückfragen zur Datenverarbeitung und zur Einhaltung des Datenschutzes wenden Sie sich bitte an den Datenschutzbeauftragten:

Behördlicher Datenschutzbeauftragter der Technischen Universität München
Postanschrift: Arcisstr. 21, 80333 München
Telefon: 089/289-17052
E-Mail: beauftragter@datenschutz.tum.de

Sie haben ebenfalls das Recht, sich bei jeder Datenschutzaufsichtsbehörde zu beschweren. Eine Liste der Aufsichtsbehörden in Deutschland finden Sie unter:
https://www.bfdi.bund.de/DE/Infothek/Anschriften_Links/anschriften_links-node.html

Die für Sie zuständige Aufsichtsbehörde erreichen Sie unter:

Bayerischer Landesbeauftragter für den Datenschutz
Postanschrift: Postfach 22 12 19, 80502 München
Hausanschrift: Wagmüllerstraße 18, 80538 München
E-Mail: poststelle@datenschutz-bayern.de

Stephan J, *et al. BMJ Open* 2024; 14:e081390. doi: 10.1136/bmjopen-2023-081390

Probandeninformation und Einwilligungserklärung zum Forschungsvorhaben: Projekt zur Entwicklung, Erprobung und Evaluierung einer psychosozialen Präventionsleistung [PE³PP] - Fokussgruppeninterviews Einwilligungserklärung

Einwilligung zur Teilnahme

Ich wurde von ____________________________ über die Studie informiert. Ich habe die schriftliche Information und Einwilligungserklärung zu der oben genannten Studie erhalten und gelesen. Ich wurde ausführlich schriftlich und mündlich über den Zweck und den Verlauf der Studie, die Chancen und Risiken der Teilnahme und meine Rechte und Pflichten aufgeklärt. Ich hatte Gelegenheit Fragen zu stellen. Diese wurden zufriedenstellend und vollständig beantwortet. Zusätzlich zur schriftlichen Information wurden folgende Punkte besprochen:

____

____

Meine Einwilligung in die Teilnahme an der Studie ist freiwillig. Ich habe das Recht, meine Einwilligung jederzeit ohne Angabe von Gründen zu widerrufen, ohne dass mir dadurch Nachteile entstehen.

Ich willige hiermit in die Teilnahme an der oben genannten Studie ein.

____
Name der teilnehmenden Person in Druckbuchstaben

____
Ort, Datum Unterschrift der teilnehmenden Person

____
Name der aufklärenden Person in Druckbuchstaben

____
Ort, Datum Unterschrift der aufklärenden Person

Einwilligung zur Datenverarbeitung

Die Verarbeitung und Nutzung der persönlichen Daten für die oben genannte Studie erfolgt ausschließlich wie in der Information zur Studie beschrieben.

Ich willige hiermit in die beschriebene Verarbeitung meiner personenbezogenen Daten, insbesondere auch Gesundheitsdaten, ein.

____
Ort, Datum Unterschrift der teilnehmenden Person

____
Ort, Datum Unterschrift der aufklärenden Person

Stephan J, *et al. BMJ Open* 2024; 14:e081390. doi: 10.1136/bmjopen-2023-081390
