# Supplementary material for: Development, piloting and evaluation of an app-supported psychosocial prevention intervention to strengthen participation in working life: a study protocol of a mixed-methods approach
Source: BMJ Open. 2024 Feb 17;14(2):e081390. doi: 10.1136/bmjopen-2023-081390 (PMC10875476; doi:10.1136/bmjopen-2023-081390)
Supplement: Author's manuscript [file bmjopen-2023-081390.draft_revisions.pdf]

BMJ Open

BMJ Open is committed to open peer review. As part of this commitment we make the peer review history of every article we publish publicly available.

When an article is published we post the peer reviewers' comments and the authors' responses online. We also post the versions of the paper that were used during peer review. These are the versions that the peer review comments apply to.

The versions of the paper that follow are the versions that were submitted during the peer review process. They are not the versions of record or the final published versions. They should not be cited or distributed as the published version of this manuscript.

BMJ Open is an open access journal and the full, final, typeset and author-corrected version of record of the manuscript is available on our site with no access controls, subscription charges or pay-per-view fees (http://bmjopen.bmj.com).

If you have any questions on BMJ Open's open peer review process please email info.bmjopen@bmj.com

BMJ Open

Development, piloting, and evaluation of an app-supported psychosocial prevention intervention: A study protocol of a mixed-methods approach

Journal:	*BMJ Open*
Manuscript ID	bmjopen-2023-081390
Article Type:	Protocol
Date Submitted by the Author:	26-Oct-2023

Complete List of Authors:	Stephan, Johannes; Technical University of Munich, TUM School of Medicine and Health, Chair of Social Determinants of Health<br>Gehrmann, Jan; Technical University of Munich, TUM School of Medicine and Health, Chair of Social Determinants of Health; Technical University of Munich, Institute of General Practice and Health Services Research<br>Stullich, Ananda; Technical University of Munich, TUM School of Medicine and Health, Chair of Social Determinants of Health<br>Hoffmann, Laura; Technical University of Munich, TUM School of Medicine and Health, Chair of Social Determinants of Health<br>Richter, Matthias; Technical University of Munich, TUM School of Medicine and Health, Chair of Social Determinants of Health<br>for the, PE³PP study group; for the PE³PP study group

Keywords:	MENTAL HEALTH, PREVENTIVE MEDICINE, Burnout, Occupational Stress, QUALITATIVE RESEARCH, Quality of Life

SCHOLARONE™
Manuscripts

Title

Development, piloting, and evaluation of an app-supported psychosocial prevention intervention: A study protocol of a mixed-methods approach

Authors:

Johannes Stephan*[1], Jan Gehrmann*[1,2], Ananda Stullich[1], Laura Hoffmann[1], Matthias Richter[1] for the PE³PP study group

Study group: Olaf Ballaschke, Sylvia Berlin, Florian Finke, Frank Gabel, Anastasia Grossmann, Claudia Gruhner, Kathleen Kaminski, Andreas Seemann (in alphabetical order)

* Both authors contributed equally to the manuscript and share the first authorship.

[1] Technical University of Munich, TUM School of Medicine and Health, Department Health and Sport Sciences, Chair of Social Determinants of Health

[2] Technical University of Munich, TUM School of Medicine and Health, Institute of General Practice and Health Services Research

Correspondence:

Johannes Stephan, Technical University of Munich, TUM School of Medicine and Health, Department Health and Sport Sciences, Chair of Social Determinants of Health, Georg-Brauchle-Ring 60/62, D-80922 Munich, Germany, Johannes.Stephan2@tum.de

For peer review only

Abstract

Introduction

Rates of incapacity to work due to mental disorders have increased in many European countries. The consequences of persistent work-related stress can impact individuals' physical and psychological well-being and gradually develop into chronic work stress. Mental disorders or symptoms of burnout syndrome can have severe consequences. Mental disorders leading to work incapacity significantly burden the health system. Prevention interventions can protect against burnout, depression, anxiety, and other mental health disorders. Digital health is a promising approach to increase the utilization of effective prevention interventions. This mixed-methods study evaluates a newly developed app-supported psychosocial prevention intervention called 'RV Fit Mental Health'.

Methods and analysis

The study uses a three-stage parallel mixed-methods design. This study accompanies the development (stage 1), piloting (stage 2), and evaluation (stage 3) of the new intervention.

Within the stages, there is a quantitative as well as a qualitative research strand. Employed persons with an incipient mental disorder will be included. Additionally, experts within the project or connected areas will be included. Quantitative data will be analyzed using multifactorial variance analyses in a pre-post design. Qualitative data will be analyzed using qualitative content analysis. The study is a comprehensive research approach to investigate the development, piloting, and evaluation of an app-supported psychosocial app-based prevention intervention. The rigor of the study will be achieved through data triangulation.

Ethics and dissemination

All participants will receive detailed study information and give written informed consent before data collection. Ethical approval was obtained from the Technical University of Munich Ethics Committee. All data collection will follow all legislative rules regarding data protection, also following the Declaration of Helsinki. The study results will be disseminated in peer-reviewed journals and presented at international conferences.

Trial registration number DRKS-ID: DRKS00030818

Wordcount: 4.464 words

Keywords

mental health care, digital health, mixed methods, study protocol, prevention

Strengths and limitations of the study

- A mixed-methods research design is used for the entire study, which allows multiple perspectives to be focused on the same topic, leading to comprehensive results.
- The generalization of quantitative and qualitative results is challenging but offers various potentials for comparison and contrast regarding other innovative projects.
- The holistic approach integrating qualitative and quantitative data collection offers the potential to analyze the peculiarities of the digital dimension of the intervention

For peer review only

1. Introduction

Over the past two decades, cases of incapacity to work due to mental disorders have increased in many European countries and represent a significant burden of disease [1, 2]. About one in five people have a current mental disorder (18% in Germany, 17.3% in the EU, 18.9% in the US, and 17.6% worldwide) [3–5]. In a study commissioned by the German health insurance company 'Techniker Krankenkasse' (TK), 47% of respondents reported that work, school, or study caused them to feel stressed. Participants stated that the main stressors were excessive workload, time pressure, rush, interruptions, and disruptions [6]. Hogan et al. described that long-term workplace stress is often caused by an imbalance between high work demands and the limited personal resources of workers [7]. The consequences of persistent work-related stress can impact individuals' physical and psychological well-being [8]. Excessive demands at work can gradually develop into chronic work stress, whereby symptoms of burnout syndrome can be consequences [8]. Long-term symptoms of burnout syndrome increase the risk of mental disorders such as sleep disorders, depressive symptoms, adjustment disorders, and physical health [8–10]. Employee performance, sick leave, absenteeism, accidents, and staff turnover are strongly influenced by mental health problems [9]. A relationship can also be shown between burnout and incapacity to work, as well as early entry into a disability pension [10].

A study by another German health insurance company, the 'Deutsche Angestellten Krankenkasse' (DAK), showed that the number of sick leave cases caused by mental health issues has almost tripled over the last 25 years [11]. The health report of the TK showed similar findings: The number of sick days due to mentally caused inability to work more than doubled from 2000 (129 sick days per 100 insurance years) to 2021 (283 sick days per 100 insurance years) in Germany. The main reasons for this increase were depressive episodes and reactions to severe stress and adjustment disorders [12]. Mental disorders cause the most days of incapacity to work at a stretch in Germany [11, 12]. Individuals who are permanently unable to work due to health reasons can apply for and receive a disability pension. The German Pension Insurance Association reports a significant increase, by double, in first-time granted disability pensions due to mental disorders in the last 25 years [13]. As a result, in the year 2021, mental disorders (41.7%) became the primary reason for disability pensions, followed by new formations such as cancer (15.3%), musculoskeletal disorders (11.5%), and cardiovascular diseases (9.1%) [13].

According to the OECD, in 2015, the costs of mental disorders in Germany accounted for approximately 4.8% of the Gross Domestic Product (GDP) [4]. Compared to the OECD-28 average, Germany's expenditures related to mental disorders are 0.7 percentage points above the average; Germany spends proportionally the second most on mental disorders in Europe [4].

For peer review only - http://bmjopen.bmj.com/site/about/guidelines.xhtml

The European Psychiatric Association states that various mental disorders can be prevented using evidence-based interventions [14]. Consequently, psychosocial prevention interventions can protect against burnout, depression, anxiety, and other mental health disorders [8, 11, 15, 16]. Effective prevention interventions strengthen environmental and individual resources while reducing risk factors [17].

In Germany, the National Prevention Conference has therefore highlighted protecting and strengthening mental health in the workplace as a priority [18]. The German Pension Insurance supports this and acts according to the principle of ‘prevention before rehabilitation’ [19]. There is a general need to expand attractive evidence-based prevention interventions and improve access to and utilization of these services [20].

Determinants of the use of digital health applications

Digital Health is a promising approach to increase the utilization and quality of prevention interventions by using information and communication technologies to meet health-related needs [21]. Studies have shown that psychosocial aspects can be strengthened during inpatient treatment [22–24]. Additionally, positive outcomes have been demonstrated for digitally supported mental health prevention interventions [25–27]. Individuals using digital health applications are exposed to various determinants influencing their effective and efficient use [28, 29]. One of the crucial determinants seems to be digital health literacy (DHL) [30]. DHL encompasses searching for and finding health information from digital sources, comprehending and assessing it, and applying acquired knowledge to address health-specific questions and solve problems [31]. It also encompasses skills and knowledge to interact productively with technology-enabled health tools [34, 35]. Other determinants that influence the utilization of digital health applications include cultural and social factors [32–34], motivation and interest [34–37], and the accessibility and availability of technology [38–40]. Subsequently, there is evidence of a relationship between DHL and adherence to digital health interventions [41, 42].

When investigating the effectiveness of an app-supported psychosocial prevention intervention, it is crucial to collect data on the different determinants that affect utilizing a digital health application. By expanding concepts of DHL to include socio-cultural factors, motivation, interest, technology accessibility, and availability, a holistic assessment of DHL can be operationalized that incorporates the relevant determinants and allows predictions about the use of a health application.

Implementation and acceptance of digital health applications

The importance of analyzing the acceptance and framework conditions and contexts of digital health applications is emphasized regarding the use of digital health applications. Therefore, it is essential

For peer review only - http://bmjopen.bmj.com/site/about/guidelines.xhtml

to account for organizational and contextual factors that impact the implementation of innovations in healthcare settings [43–45]. This is particularly important as it has been shown that evidence of effectiveness has not readily led to sustained implementation [46]. Especially in mental healthcare, the implementation of digital health applications poses various and unique challenges, highlighting the importance of evaluating the effectiveness and factors for successful implementation [43, 47]. Various studies highlight that it is necessary to analyze the interrelation between digital intervention and the healthcare context in which it is implemented [48, 49]. Thus, many digital mental health care interventions show better effects when paired with some therapeutic or doctoral support [50]. It is also highlighted that the perspective of health professionals as well as patients needs to be analyzed in the development to enable successful implementation [51–53]. Analyzing the implementation of a digital intervention needs to apply a comprehensive approach using quantitative and qualitative data. As the development, piloting, and evaluation is an iterative process, there is a need to analyze the different stages, anticipating the various perspectives involved, like users as well as healthcare professionals, but also focus on the contextual factors like organizational routines, established processes, and regulatory or policy aspects [49, 54].

Problem summary

In summary, the following problems were identified: First, the stress experienced by individuals in their work environment can become chronic, negatively affecting their mental and physical well-being. Consequently, this places an increasing burden on the healthcare systems. Second, prevention interventions can protect against the chronification of work-related stress; however, prevention interventions are rarely taken up in Germany. Third, digitally enabled prevention interventions can increase utilization. However, it cannot be assumed that different people can use digital health applications equally effectively. Fourth, there are measurement tools for assessing DHL, but existing measurement tools do not account for potential determinants that may impact the effective use of digital health applications. Fifth, regarding developing and implementing a new digital intervention, there is a need to analyze the acceptance and contextual factors.

The study aims to investigate the development, piloting, and evaluation of a psychosocial app-based prevention intervention using a comprehensive mixed methods approach. The study protocol was developed using the SPIRIT checklist [55].

2. Methods and analysis

Study background

The project ‘Development, piloting, and evaluation of an app-supported psychosocial prevention intervention’ (German acronym PE³PP)” is funded under the federal program ‘Innovative Ways to

For peer review only - http://bmjopen.bmj.com/site/about/guidelines.xhtml

Participate in Working Life – rehapro' of the Federal Ministry of Labor and Social Affairs Germany. The project runs from October 2021 until September 2026 and is led by the German Pension Insurance Central Germany.

This study protocol outlines the planned scientific accompaniment for developing, piloting, and evaluating a psychosocial app-supported prevention intervention called 'RV Fit Mental Health' by the Chair of Social Determinants of Health of the Technical University of Munich (TUM). The intervention aims to promote and strengthen individuals' participation in work life. It is a prospective multicentric study. The two central German rehabilitation clinics, Median Klinik Bad Gottleuba and SRH Medinet Burgenlandklinik, will lead in developing and conducting the intervention. Further PE³PP project partners are the health insurance companies 'Allgemeine Ortskrankenkasse Sachsen-Anhalt' (AOK SAN) in the State of Saxony-Anhalt and 'Allgemeine Ortskrankenkasse PLUS' (AOK PLUS) in the states of Saxony and Thuringia.

Study intervention

Innovation of the intervention

The psychosocial prevention intervention 'RV Fit Mental Health' aims to improve existing prevention interventions of the German Pension Insurance. The secondary prevention intervention is conceptually innovative, focusing on the psychological, psychosomatic, and psychosocial aspects of work-related participation disorders. It is formally innovative in terms of its temporal and methodological design. The duration of the intervention will be 14 weeks. It commences with a two-week inpatient initial phase in a rehabilitation clinic and a 12-week app-supported training phase. Participants receive intensive therapeutic support during the inpatient and training phases. The proactive recruitment of participants by German Pension Insurance Central Germany and the two health insurances, AOK SAN and AOK Plus, is also promising.

The framework of the intervention

The study is multicentric and will be conducted simultaneously at two clinics. 1080 participants are expected to undergo the intervention from September 2023 to September 2026. Participants complete the two-week inpatient phase in small groups of ten participants per clinic. Each participant will be assigned a therapeutic support person for both intervention phases. During the inpatient phase, the participant and therapeutic support person collaboratively establish prevention goals, resulting in a therapy plan for the inpatient and training phases.

Modules of the intervention

For peer review only

The prevention intervention covers various topics, including psychological stabilization, self-care, finding meaning and purpose, coping with multiple stressors and increasing digitalization, achieving work-life balance, and providing ‘first aid’ on topics such as burnout, depression, anxiety, sleep disorders, and pain. In addition to psychoeducational elements, the intervention includes training in social and emotional competencies, professional and personal stress management, interaction, and communication training, as well as the provision of mindfulness-based and body-oriented therapeutic elements. These substantive topics will be consolidated into modules, conveyed, and practiced during the inpatient and training phases.

Didactical realization of the intervention

During the inpatient phase, therapists conduct the modules in groups. In this process, all participants attend most of the modules. Additionally, there will be specific modules tailored to the individual needs of the participants. The content of the modules is conveyed and trained through presentations, lectures, exercises, and self-development phases.

In the training phase, the participants could flexibly arrange their training in terms of time and location. The training phase will be supported by an app, creating a digital connection between the rehabilitation clinic and participants. The app will contain the training plan designed by the therapeutic support person to achieve the prevention goals. The training plan will include various modules for practice during this phase.

The app provides various methods and formats for delivering module content, such as exercises, instructions, training, and seminars in video and audio formats. In addition, interaction and communication features, including group chats and video call functions, enable participants to engage with their therapeutic support person and other participants. Consequently, the app supports and accompanies the transfer of knowledge and behaviors initiated during the inpatient phase into the participants' daily lives.

During the training phase, each participant will have a coaching session with their therapeutic support person every four weeks. Through the coaching sessions, the therapeutic support person and the participant will (re)evaluate the progress toward achieving the prevention goals. Moreover, group sessions will take place during the training phase, where participants from the same group in the inpatient phase and a therapeutic support person will work on relevant intervention content and exchange experiences within the group. In the 14th week of the prevention intervention, each participant will have an individual concluding session with their therapeutic support person, ending the intervention.

Design, aims, and research questions

For peer review only - http://bmjopen.bmj.com/site/about/guidelines.xhtml

The study follows a parallel mixed-methods design integrating quantitative and qualitative data [56–58]. This approach offers the opportunity to highlight multiple perspectives on the same topic and consequently betters comprehension. Mixed-methods research has several methodological challenges, especially regarding study rigor, data triangulation, and integration of the various data [57–59]. Data triangulation is a methodological approach that will contribute to the validity of the results by linking qualitative and quantitative data, offering various potentials for analysis through the dynamic and emergent process of data collection and analysis [60–63]. This is especially beneficial in evaluation and implementation research [64].

The study consists of three stages in which various research questions are answered (Figure 1). In stage one, we will collect qualitative data. Stages two and three have a qualitative and quantitative research strand with corresponding objectives and research questions.

Figure 1: Overview of the study stages

Stage 1: Development (July 2022 – August 2023)

Stage one (S1) aims to scientifically accompany the development of the 'RV Fit Mental Health' intervention. The central research question is: What needs to be done to provide an attractive, innovative psychosocial prevention intervention?

In this stage, the different dimensions of the development process and the requirements for the organizational implementation are of interest. This stage consists of a qualitative research strand. The results thus have an evaluative character in that they enable the optimization and adaptation of the needs of the experts and provide indications for successful cooperation between the actors.

Qualitative research questions

We will answer the following sub-research questions:

- What are the needs and requirements of project actors with regard to the development of psychosocial intervention?
- Does the app fulfill the required functionality for the app-supported training phase?

Stage 2: Piloting (September 2023 – December 2023)

In stage two (S2), we aim to answer the central research question: How should the psychosocial prevention intervention 'RV Fit Mental Health' be implemented?

For peer review only - http://bmjopen.bmj.com/site/about/guidelines.xhtml

We will pilot the intervention and assess its feasibility. Thus, three research objectives emerged for stage two. First, the needs and experiences of the participants will be analyzed to identify standards for addressing potential participants and to optimize the intervention. Second, the constructed instrument for evaluating the intervention will undergo a pretest and a feasibility assessment. As the instrument will include an assessment of DHL, we aim to identify additional skills, competencies, and prerequisites that need to be analyzed to predict effectiveness. This results in five qualitative and one quantitative sub-research questions for stage two.

Qualitative research questions

- How should an app-supported prevention intervention be designed and organized to be feasible and accepted by the participants and the experts?
- What needs, expectations, and experiences were the first participants able to perceive during the initial run-through of the prevention intervention?
- What are facilitating factors and barriers regarding the implementation of the intervention?
- Which possibilities for optimization occur to increase the fit and acceptance of the prevention intervention, especially regarding the fit between the inpatient and the training phase?
- What competencies, skills, and prerequisites should users of digital health apps possess besides digital health literacy?

Quantitative research question

- Is the constructed survey instrument to measure primary and secondary outcomes feasible for participants?

__Stage 3: Evaluation (January 2023 – April 2026)__

Stage three (S3) aims to analyze the implementation and execution of the intervention. The key research questions in the evaluation stage are: Is the implementation of the psychosocial prevention intervention achieved? Is the psychosocial prevention intervention successful?

We will examine organizational conditions, accessibility of the target group, and collaboration among actors to implement the prevention intervention. We will investigate this from the perspectives of professionals and intervention participants. Additionally, we will assess the effectiveness of the intervention. This results in six qualitative and four quantitative sub-research questions for stage three.

Qualitative research questions

For peer review only - http://bmjopen.bmj.com/site/about/guidelines.xhtml

- Is the implementation of the psychosocial prevention intervention successful? What facilitating factors and barriers occurred?
- Is the app-supported prevention intervention feasible and accepted by the participants and the experts?
- What are the conditions for the implementing prevention intervention in terms of organizational processes and resources? What are the requirements from the health care professional’s point of view?
- How is the dovetailing between the inpatient and the digital phase? What are the facilitating factors and barriers in the independent use of the app, and what are the determinants for successful therapeutic support in the digital phase?
- How does the cooperation and collaboration of the different actors develop during the project?
- What are the potentials, challenges, and needs for adaptation?

Quantitative research questions

- Can the intervention strengthen workability?
- Can the intervention improve the quality of life?
- Can the intervention reduce work-related stress levels?
- Can the level of digital health literacy affect workability, quality of life, or work-related stress levels?

Intervention participants and study sampling

Participants in the prevention intervention

There are two pathways through which individuals are recruited for the prevention intervention. Pathway one is carried out by the German Pension Insurance Central Germany, a regional institution responsible for two million employed people and over one million pensioners. Pathway two is carried out by the health insurance companies AOK SAN and AOK PLUS, regional institutions of the Health Insurance AOK in the east of Germany, insuring a combined 4.3 million citizens. The three insurance companies will contact eligible insureds and proactively suggest the RV Fit Mental Health prevention intervention.

Sample and recruitment for all study stages

For peer review only

Quantitative Sample - Participants of the Intervention (S2, S3)

We aim to survey all participants from the intervention's piloting (S2) and evaluation (S3) stages. We will use a non-probability sample for recruitment. Participants in the prevention intervention will receive a flyer on recruitment for our study via the German Pension Insurance and the intervention clinics. On the flyer are instructions and online access for our quantitative data collection. In S2, we expect a total of 60 participants. In S3, we intend to include 1020 participants for the pre-post-design effectiveness assessment. We expect dropouts, especially during the digital training phase.

Qualitative Sample - Participants of the Intervention (S2, S3)

Focus groups (FG) with intervention participants will be conducted during the piloting (S2) and evaluation (S3) stages. We will sample through a random sampling method, where data collection time points will be predetermined. We plan four FG in the S2 with 6-10 participants each. Ten FG, also with 6-10 participants each, are planned in S3. Overall, we plan up to 14 FG with intervention participants. Contact with the individual group participants for the qualitative data collection is initiated via the respective clinic staff. If there is interest in participation, the clinics forward the time and number of participants.

Qualitative Sample - Experts (S1, S2, S3)

Expert interviews with representatives of the German Pension Insurance Central Germany, the AOK SAN, and AOK Plus, and with the executing healthcare professionals from the intervention clinics, will be conducted during the development (S1) and evaluation (S3) stages. There will also be FG in the pilot (S2) stage with these experts. Experts involved in planning and conducting the intervention are included in all stages. Up to ten participants will participate in each point of the data collection. The TUM project team will proactively recruit the participants of the expert interviews and the FG.

Furthermore, additional expert interviews will be conducted in S2 with healthcare professionals who have prescribed or recommended app-based (digital) health applications for disease management, health promotion, or prevention as part of a treatment pathway. This may include physicians, psychologists, and psychotherapists but exclude people who have prescribed or recommended other digital health apps, such as wearables (smartwatches, trackers, etc.), and people who do not work in healthcare. Likewise, we are recruiting individuals who are involved in health research and researching the subject matter of digital health and digital health literacy. We will interview ten to twelve experts. The experts will be recruited via professional associations and proactively through the study team.

Data collection

For peer review only - http://bmjopen.bmj.com/site/about/guidelines.xhtml

Quantitative data collection

Quantitative data will be collected from the study participants in S2 and S3 at four different points (t0-t3) through (online) surveys, depicted graphically in Figure 2.

- t0 (first survey) transpires when insured persons are confirmed that participation in the prevention intervention is possible.
- t1 (second survey) takes place at the beginning of the prevention intervention within the first two days of the initial inpatient phase.
- t2 (third survey) is conducted two weeks after t1, at the end of the initial inpatient phase.
- t3 (fourth survey) occurs 12 weeks after the inpatient phase at the end of the 12-week training phase.

Figure 2: Overview of quantitative data collection

The primary outcomes of interest are workability, assessed using the German Work Ability Index [65]; quality of life, using the German WHOQOL-BREF [66]; stress levels, evaluated using the DASS-21 [67]; and eHealth literacy, which will be assessed using the G-eHEALS [68, 69], and a self-developed instrument for holistic DHL.

The secondary outcomes are anxiety and depressive symptoms (DASS-21) and self-efficacy using the SWOP K9 [70]. In addition, we are interested in health behavior as a secondary outcome, which we operationalize as alcohol consumption, smoking, physical activity, and dietary behavior. The training phase behavioral data follow the FITT criteria [71, 72]: frequency, intensity, time, and type. We will use FITT data to examine participants' adherence to the eHealth application [73]. Additionally, sociodemographic data will be collected during survey t1, and behavioral data from the training phase will be gathered during survey t3.

For peer review only

Qualitative data collection

The overall qualitative data collection is depicted graphically in Figure 3.

Figure 3: Overview of qualitative data collection

*1*The FG with participants occurs both during the initial inpatient phase on-site at the clinics and online during the digital training phase. The FG in the inpatient phase occurs towards the end of the inpatient stay. The online FG takes place when at least four weeks of the digital training phase have been completed.

The interviews with the experts will be conducted online. Depending on the research questions in each stage, a semi-structured interview guide will be developed and pretested for each expert group. The interview guides will be developed following the principles of Helfferich [74]. Furthermore, we are following the methodical principles of expert interviews and recognize the context of the new intervention as well as the evaluative character of the expert interviews [75–77].

The FG with the experts will take place during a project workshop on-site. The FG is based on a purposive moderation guide starting with a stimulus recognizing the specified topics, the organizational contexts, and the environment of the intervention [78].

Data analysis

Quantitative analysis

Quantitative data analysis will be conducted using the IBM *Statistical Package for Social Sciences* (SPSS) software. During S2, the dataset will undergo a preliminary examination for meaningfulness. This involves checking for the data's completeness, outliers, and plausibility. A descriptive characterization of the quantitative S2 sample will be performed based on sociodemographic data to gain initial insights into the sample.

For S3, we plan a pre-post design effectiveness evaluation. The baseline data will be compared with the data from t1-t3. For this, we will conduct multifactorial variance analyses to investigate whether there are differences in the groups of individual intervention time points regarding the primary and secondary outcomes, thus demonstrating the potential effect of the prevention intervention. Additionally, we will perform regression analyses to interpret the relationship between the collected variables, allowing for statements about the influence of individual variables on different outcomes. Similarly, we present a differentiated sample characterization for S3.

Qualitative analysis

The semi-structured interviews and the FG will be conducted by the research team (JG, JS), audiotaped, verbatim transcribed, and analyzed following Content Analysis, using MAXQDA 2022 [79, 80]. Qualitative content analysis is a systematic, rule-bound approach orientated towards rules of text analysis laid down in advance and contributes to the intersubjectivity of the analysis procedure regarding the defined interests [80]. The analytical steps taken by the category system are, firstly, the summary and the inductive category formation, then secondly by the explication and the context analysis, finalized by the structuring and the deductive category formation. The category system will then be re-checked by applying it to the material, the research questions, and the case itself [72]. Constant exchange within the study team and the working group for Qualitative Research at the Chair

For peer review only - http://bmjopen.bmj.com/site/about/guidelines.xhtml

for Social Determinants of Health will ensure the rigor and integrity of the analysis. The analysis will be framed by applying content-analytical quality criteria and reported using the consolidated criteria for reporting qualitative research (COREQ) [73].

3. Ethics and dissemination

Ethical approval was obtained from the Ethics Committee at the TUM School of Medicine and Health (2022-523-S-SR; 2023-316-S-SB). Following the Declaration of Helsinki, we will conduct the study according to ethical research principles, including the primacy of participant welfare, informed consent, scientific validity, review by an ethics committee, appropriate risk-benefit balance, protection of vulnerable groups, data security and confidentiality, transparent reporting, and voluntary participation. If any study participant decides not to participate or not continue the study, they may withdraw consent at any time without any consequences. All data management follows the requirements of the German version of the General Data Protection Regulation and is approved by the TUM internal department for data security. All data collected in the study will be pseudonymized or, if possible, anonymized. The pseudonymization through a code ensures data protection of all data. All data containing identificatory characteristics (e.g., record, pseudonym assignment list, etc.) is secured separately from study data. They are stored in locked rooms at the Chair of Social Determinants of Health. We will delete all data once the study has been completed, but at the latest, after the given legal restriction of 10 years. In case of withdrawal, all data will be deleted entirely or, in extraordinary cases, evaluated carefully anonymized. Deleting data from already analyzed or published data is not possible. We will publish all analyzed data without any possibility of identifying persons. All results of the quantitative analysis will be published anonymously, and the results of the qualitative analysis will be pseudonymized. The study results will be disseminated in international peer-reviewed journals and presented at international conferences.

4. References

1 Hildebrandt-Heene S, Dehl T, Zich K, et al. Gesundheitsreport 2023: Analyse der Arbeitsunfähigkeiten. Gesundheitsrisiko Personalmangel: Arbeitswelt unter Druck. Heidelberg: medhochzwei Verlag GmbH 2023.

2 Salomonsson S, Hedman-Lagerlöf E, Öst L-G. Sickness absence: a systematic review and meta-analysis of psychological treatments for individuals on sick leave due to common mental disorders. *Psychol Med* 2018;48(12):1954–65. doi:10.1017/S0033291718000065 [published Online First: 30 January 2018].

3 Müller G, Bombana M, Heinzel-Gutenbrenner M, et al. Socio-economic consequences of mental distress: quantifying the impact of self-reported mental distress on the days of incapacity to work and medical costs in a two-year period: a longitudinal study in Germany. *BMC Public Health* 2021;21(1):625. doi:10.1186/s12889-021-10637-8 [published Online First: 31 March 2021].

4 OECD. Health at a Glance: Europe 2018. Brussels: OECD 2018.

For peer review only - http://bmjopen.bmj.com/site/about/guidelines.xhtml

5 Steel Z, Marnane C, Iranpour C, et al. The global prevalence of common mental disorders: a systematic review and meta-analysis 1980-2013. *Int J Epidemiol* 2014;43(2):476–93. doi:10.1093/ije/dyu038 [published Online First: 19 March 2014].
6 Meyer B, Zill A, Dibla D. Entspann dich, Deutschland!: TK Stressstudie 2021. Hamburg: Technicker Krankenkasse 2021.
7 Hogan L-M, Björklund Carlstedt A, Wagman P. Occupational therapy and stress-related exhaustion - a scoping review. *Scand J Occup Ther* 2023:1–17. doi:10.1080/11038128.2023.2207802 [published Online First: 8 June 2023].
8 Edú-Valsania S, Laguía A, Moriano JA. Burnout: A Review of Theory and Measurement. *Int J Environ Res Public Health* 2022;19(3). doi:10.3390/ijerph19031780 [published Online First: 4 February 2022].
9 Wilken U, Breucker G. Mental health in the workplace: Situation analysis Germany. Geneva: International Labour Organization 2000.
10 Ahola K, Gould R, Virtanen M, et al. Occupational burnout as a predictor of disability pension: a population-based cohort study. *Occup Environ Med* 2009;66(5):284-90; discussion 282-3. doi:10.1136/oem.2008.038935 [published Online First: 18 November 2008].
11 Marschall J, Hildebrandt S, Nolting H-D. DAK-Gesundheitsreport 2019 2019. https://www.dak.de/dak/download/dak-gesundheitsreport-2019-sucht-pdf-2073718.pdf.
12 Grobe T, Bessel S. Gesundheitsreport 2022 Arbeitsunfähigkeit. Göttingen: aQua – Institut für angewandte Qualitätsförderung und Forschung im Gesundheitswesen GmbH 2022.
13 German Pension Insurance Association. Erwerbsminderungsrenten im Zeitablauf 2022 2022. Available at: https://www.deutsche-rentenversicherung.de/DRV/DE/Experten/Zahlen-und-Fakten/Statistiken-und-Berichte/statistiken-und-berichte_node.html Accessed July 27, 2023.
14 Campion J, Bhui K, Bhugra D. European Psychiatric Association (EPA) guidance on prevention of mental disorders. *Eur Psychiatry* 2012;27(2):68–80. doi:10.1016/j.eurpsy.2011.10.004 [published Online First: 28 January 2012].
15 Lehofer M, Glehr R, Haring C, et al. Burnout & Depression. Ein Leitfaden zur Prävention, Früherkennung und Behandlung. Experten-Statement [Burnout and Depression. A Guideline for Prevention, Early Diagnosis, and Intervention. Experts Statement] 2011.
16 Singh V, Kumar A, Gupta S. Mental Health Prevention and Promotion-A Narrative Review. *Front Psychiatry* 2022;13:898009. doi:10.3389/fpsyt.2022.898009 [published Online First: 26 July 2022].
17 World Health Organization. Mental Health 2022. Available at: https://www.who.int/news-room/fact-sheets/detail/mental-health-strengthening-our-response Accessed July 25, 2023.
18 National Prevention Conference. Spezielle Ziele für die Zielgruppe Erwerbstätige 2022. Available at: https://www.npk-info.de/praeventionsstrategie/bundesrahmenempfehlungen/spezielle-ziele-fuer-die-zielgruppe-erwerbstaetige Accessed July 26, 2023.
19 German Pension Insurance Association. Reha-Bericht 2021. Berlin: H. Heenemann GmbH & Co. KG 2021.
20 Robert Koch-Institut. Wie steht es um Prävention und Gesundheitsförderung?: RKI-Bib1 (Robert Koch-Institut) 2015.
21 World Health Organization. Global strategy on digital health 2020-2025. Geneve: World Health Organization 2021.

For peer review only

22 Booth S, Ryan R, Clow A, et al. Enhancing well-being and building resilience in people living with cancer part 2: a central role for nurses. *Cancer Nursing Practice* 2019;18(1):42–50.

23 Goldbach N, Reif A, Preuss H, et al. A randomized trial of group therapy for resource activation for patients with affective disorder. *Journal of Psychotherapy Integration* 2023;33(1):1–19.

24 Breuer K, John SM, Finkeldey F, et al. Tertiary individual prevention improves mental health in patients with severe occupational hand eczema. *J Eur Acad Dermatol Venereol* 2015;29(9):1724–31. doi:10.1111/jdv.12975 [published Online First: 4 February 2015].

25 Bostock S, Crosswell AD, Prather AA, et al. Mindfulness on-the-go: Effects of a mindfulness meditation app on work stress and well-being. *J Occup Health Psychol* 2019;24(1):127–38.

26 Champion L, Economides M, Chandler C. The efficacy of a brief app-based mindfulness intervention on psychosocial outcomes in healthy adults: A pilot randomised controlled trial. *PLoS One* 2018;13(12):e0209482.

27 Zhou T, Li X, Pei Y, et al. Internet-based cognitive behavioural therapy for subthreshold depression: a systematic review and meta-analysis. *BMC Psychiatry* 2016;16(1):356.

28 Li J, Theng Y-L, Foo S. Predictors of online health information seeking behavior: Changes between 2002 and 2012. *Health Informatics J* 2016;22(4):804–14. doi:10.1177/1460458215595851 [published Online First: 10 August 2015].

29 Perich T, Andriessen K. Predictors of digital technology-based mental health programs in young adults for mental health support. *Health Promot Int* 2023;38(3).

30 van Kessel R, Wong BLH, Clemens T, et al. Digital health literacy as a super determinant of health: More than simply the sum of its parts. *Internet Interv* 2022;27:100500. doi:10.1016/j.invent.2022.100500 [published Online First: 7 February 2022].

31 Norman CD, Skinner HA. eHealth Literacy: Essential Skills for Consumer Health in a Networked World. *J Med Internet Res* 2006;8(2):e9. doi:10.2196/jmir.8.2.e9 [published Online First: 16 June 2006].

32 Ehrari H, Tordrup L, Müller S. The Digital Divide in Healthcare: A Socio-Cultural Perspective of Digital Literacy. Proceedings of the 55th Hawaii International Conference on System Sciences 2022 2022.

33 Hsieh JJP-A, Rai A, Keil M. Addressing Digital Inequality for the Socioeconomically Disadvantaged Through Government Initiatives: Forms of Capital That Affect ICT Utilization. *Information Systems Research* 2011;22(2):233–53.

34 Sifat MS, Saperstein SL, Tasnim N, et al. Motivations Toward Using Digital Health and Exploring the Possibility of Using Digital Health for Mental Health in Bangladesh University Students: Cross-sectional Questionnaire Study. *JMIR Form Res* 2022;6(3):e34901. doi:10.2196/34901 [published Online First: 4 March 2022].

35 Dias N, Yamamoto R, Faulkner KG. Preferences and motivational factors for the use of digital technology in real world oncology studies. *JCO* 2020;38(15_suppl):e14119-e14119.

36 Kaihlanen A-M, Virtanen L, Buchert U, et al. Towards digital health equity - a qualitative study of the challenges experienced by vulnerable groups in using digital health services in the COVID-19 era. *BMC Health Serv Res* 2022;22(1):188. doi:10.1186/s12913-022-07584-4 [published Online First: 12 February 2022].

37 Park DY, Kim H. Determinants of Intentions to Use Digital Mental Healthcare Content among University Students, Faculty, and Staff: Motivation, Perceived Usefulness,

For peer review only - http://bmjopen.bmj.com/site/about/guidelines.xhtml

Perceived Ease of Use, and Parasocial Interaction with AI Chatbot. *Sustainability* 2023;15(1):872.
38 World Health Organization. Equity within digital health technology within the WHO European Region: a scoping review. Copenhagen: WHO Regional Office for Europe 2022.
39 Sieck CJ, Sheon A, Ancker JS, et al. Digital inclusion as a social determinant of health. *NPJ Digit Med* 2021;4(1):52. doi:10.1038/s41746-021-00413-8 [published Online First: 17 March 2021].
40 Richardson S, Lawrence K, Schoenthaler AM, et al. A framework for digital health equity. *NPJ Digit Med* 2022;5(1):119. doi:10.1038/s41746-022-00663-0 [published Online First: 18 August 2022].
41 Lu X, Zhang R. Association Between eHealth Literacy in Online Health Communities and Patient Adherence: Cross-sectional Questionnaire Study. *J Med Internet Res* 2021;23(9):e14908. doi:10.2196/14908 [published Online First: 13 September 2021].
42 Do BN, Tran TV, Phan DT, et al. Health Literacy, eHealth Literacy, Adherence to Infection Prevention and Control Procedures, Lifestyle Changes, and Suspected COVID-19 Symptoms Among Health Care Workers During Lockdown: Online Survey. *J Med Internet Res* 2020;22(11):e22894. doi:10.2196/22894 [published Online First: 12 November 2020].
43 Graham AK, Lattie EG, Powell BJ, et al. Implementation strategies for digital mental health interventions in health care settings. *Am Psychol* 2020;75(8):1080–92.
44 Powell BJ, Waltz TJ, Chinman MJ, et al. A refined compilation of implementation strategies: results from the Expert Recommendations for Implementing Change (ERIC) project. *Implement Sci* 2015;10:21. doi:10.1186/s13012-015-0209-1 [published Online First: 12 February 2015].
45 Powell BJ, McMillen JC, Proctor EK, et al. A compilation of strategies for implementing clinical innovations in health and mental health. *Med Care Res Rev* 2012;69(2):123–57. doi:10.1177/1077558711430690 [published Online First: 26 December 2011].
46 Mohr DC, Weingardt KR, Reddy M, et al. Three Problems With Current Digital Mental Health Research . and Three Things We Can Do About Them. *Psychiatr Serv* 2017;68(5):427–29. doi:10.1176/appi.ps.201600541 [published Online First: 17 April 2017].
47 Mohr DC, Lyon AR, Lattie EG, et al. Accelerating Digital Mental Health Research From Early Design and Creation to Successful Implementation and Sustainment. *J Med Internet Res* 2017;19(5):e153. doi:10.2196/jmir.7725 [published Online First: 10 May 2017].
48 Marwaha JS, Landman AB, Brat GA, et al. Deploying digital health tools within large, complex health systems: key considerations for adoption and implementation. *npj Digit. Med.* 2022;5(1):13. doi:10.1038/s41746-022-00557-1 [published Online First: 27 January 2022].
49 Shaw J, Agarwal P, Desveaux L, et al. Beyond "implementation": digital health innovation and service design. *npj Digital Med* 2018;1(1):48. doi:10.1038/s41746-018-0059-8 [published Online First: 20 September 2018].
50 Lattie EG, Stiles-Shields C, Graham AK. An overview of and recommendations for more accessible digital mental health services. *Nat Rev Psychol* 2022;1(2):87–100. https://www.nature.com/articles/s44159-021-00003-1.
51 Birnbaum F, Lewis D, Rosen RK, et al. Patient engagement and the design of digital health. *Acad Emerg Med* 2015;22(6):754–56. doi:10.1111/acem.12692 [published Online First: 21 May 2015].

For peer review only - http://bmjopen.bmj.com/site/about/guidelines.xhtml

52 Lukka L, Karhulahti V-M, Palva JM. Factors Affecting Digital Tool Use in Client Interaction According to Mental Health Professionals: Interview Study. *JMIR Hum Factors* 2023;10:e44681. doi:10.2196/44681 [published Online First: 10 July 2023].

53 Mendes-Santos C, Nunes F, Weiderpass E, et al. Understanding Mental Health Professionals' Perspectives and Practices Regarding the Implementation of Digital Mental Health: Qualitative Study. *JMIR Form Res* 2022;6(4):e32558. doi:10.2196/32558 [published Online First: 12 April 2022].

54 Desveaux L, Soobiah C, Bhatia RS, et al. Identifying and Overcoming Policy-Level Barriers to the Implementation of Digital Health Innovation: Qualitative Study. *J Med Internet Res* 2019;21(12):e14994. doi:10.2196/14994 [published Online First: 20 December 2019].

55 Chan AW, Tetzlaff JM, Altman DG, et al. SPIRIT 2013 statement: defining standard protocol items for clinical trials. *Ann Intern Med* 2013;158(3):200–07.

56 Tariq S, Woodman J. Using mixed methods in health research. *JRSM Short Rep* 2013;4(6):2042533313479197. doi:10.1177/2042533313479197 [published Online First: 7 May 2013].

57 Kuckartz U. Mixed Methods: Methodologie, Forschungsdesigns und Analyseverfahren: Springer Fachmedien Wiesbaden 2014.

58 Fetters MD, Curry LA, Creswell JW. Achieving integration in mixed methods designs-principles and practices. *Health Services Research* 2013;48(6 Pt 2):2134–56. doi:10.1111/1475-6773.12117 [published Online First: 23 October 2013].

59 Wisdom JP, Cavaleri MA, Onwuegbuzie AJ, et al. Methodological reporting in qualitative, quantitative, and mixed methods health services research articles. *Health Services Research* 2012;47(2):721–45. doi:10.1111/j.1475-6773.2011.01344.x [published Online First: 8 November 2011].

60 Casey D, Murphy K. Issues in using methodological triangulation in research. *Nurse Researcher* 2009;16(4):40–55.

61 Farmer T, Robinson K, Elliott SJ, et al. Developing and implementing a triangulation protocol for qualitative health research. *Qualitative Health Research* 2006;16(3):377–94.

62 Gibson CB. Elaboration, Generalization, Triangulation, and Interpretation. *Organizational Research Methods* 2017;20(2):193–223.

63 Jick TD. Mixing Qualitative and Quantitative Methods: Triangulation in Action. *Administrative Science Quarterly* 1979;24(4):602.

64 Green CA, Duan N, Gibbons RD, et al. Approaches to Mixed Methods Dissemination and Implementation Research: Methods, Strengths, Caveats, and Opportunities. *Adm Policy Ment Health* 2015;42(5):508–23.

65 Freyer M. Eine Konstruktvalidierung des Work Ability Index anhand einer repräsentativen Stichprobe von Erwerbstätigen in Deutschland: Bundesanstalt für Arbeitsschutz und Arbeitsmedizin (BAuA) 2019.

66 World Health Organization. WHOQOL. User Manual. Geneva: World Health Organization 2012.

67 Nilges P, Essau C. DASS. Depressions-Angst-Stress-Skalen - deutschsprachige Kurzfassung: ZPID (Leibniz Institute for Psychology) – Open Test Archive 2021.

68 Norman CD, Skinner HA. eHEALS: The eHealth Literacy Scale. *J Med Internet Res* 2006;8(4):e27. doi:10.2196/jmir.8.4.e27 [published Online First: 14 November 2006].

69 Soellner R, Huber S, Reder M. The Concept of eHealth Literacy and Its Measurement. *Journal of Media Psychology* 2014;26(1):29–38.

For peer review only - http://bmjopen.bmj.com/site/about/guidelines.xhtml

70 Scholler G, Fliege H, Klapp BF. SWOP-K9 - Fragebogen zu Selbstwirksamkeit-Optimismus-Pessimismus Kurzform: ZPID (Leibniz Institute for Psychology Information) – Testarchiv 1999.
71 Barisic A, Leatherdale ST, Kreiger N. Importance of frequency, intensity, time and type (FITT) in physical activity assessment for epidemiological research. *Can J Public Health* 2011;102(3):174–75.
72 Short CE, DeSmet A, Woods C, et al. Measuring Engagement in eHealth and mHealth Behavior Change Interventions: Viewpoint of Methodologies. *J Med Internet Res* 2018;20(11):e292. doi:10.2196/jmir.9397 [published Online First: 16 November 2018].
73 Sieverink F, Kelders SM, van Gemert-Pijnen JE. Clarifying the Concept of Adherence to eHealth Technology: Systematic Review on When Usage Becomes Adherence. *J Med Internet Res* 2017;19(12):e402. doi:10.2196/jmir.8578 [published Online First: 6 December 2017].
74 Helfferich C. Die Qualität qualitativer Daten: Manual für die Durchführung qualitativer Interviews, 4th edn. Wiesbaden: VS Verlag 2011.
75 Meuser M, Nagel U. The Expert Interview and Changes in Knowledge Production. In: Interviewing Experts: Palgrave Macmillan, London 2009:17–42.
76 Döringer S. 'The problem-centred expert interview'. Combining qualitative interviewing approaches for investigating implicit expert knowledge. *International Journal of Social Research Methodology* 2021;24(3):265–78.
77 Wroblewski A, Leitner A. Between Scientific Standards and Claims to Efficiency: Expert Interviews in Programme Evaluation. In: Interviewing Experts: Palgrave Macmillan, London 2009:235–51.
78 Smithson J. Using and analysing focus groups: Limitations and possibilities. *International Journal of Social Research Methodology* 2000;3(2):103–19.
79 Mayring P. Qualitative Content Analysis. *Forum qualitative Sozialforschung* 2000;1(2).
80 Mayring P. Qualitative content analysis: theoretical foundation, basic procedures and software solution. Klagenfurt 2014.

5. Authors´ contributions

JG and JS wrote the initial manuscript draft, will collect the quantitative and qualitative data, and are responsible for data analyses. LH and MR were involved in the grant application. JS and JG wrote the study protocol for the Ethics Review Committee. LH, AS, and MR critically reviewed the article several times and counseled the entire process of this study protocol and research. All authors have read and approved the final version of the manuscript.

6. Funding statement

This study is supported by the Federal Ministry of Labour and Social Affairs as part of the rehapro funding program and is led by the German Pension Insurance of Central Germany (662S0033X1).

7. Competing interests statement

The authors of this study declare no conflicts of interest.

For peer review only

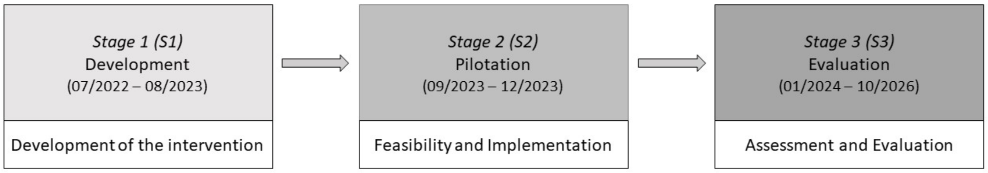

Figure 1: Overview of the study stages

338x190mm (96 x 96 DPI)

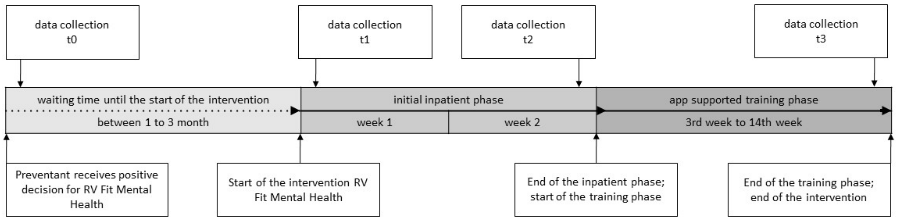

Figure 2: Overview of quantitative data collection

338x190mm (96 x 96 DPI)

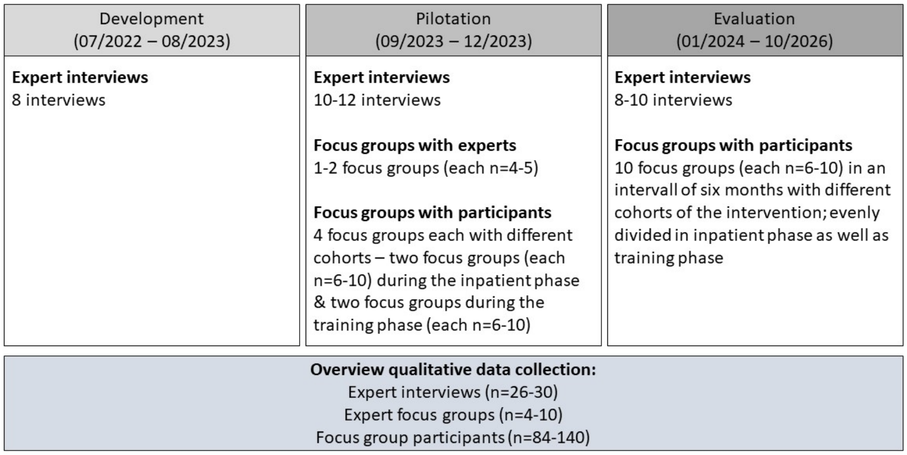

Figure 3: Overview of qualitative data collection

338x190mm (96 x 96 DPI)

Reporting checklist for protocol of a clinical trial.

Based on the SPIRIT guidelines.

Instructions to authors

Complete this checklist by entering the page numbers from your manuscript where readers will find each of the items listed below.

Your article may not currently address all the items on the checklist. Please modify your text to include the missing information. If you are certain that an item does not apply, please write "n/a" and provide a short explanation.

Upload your completed checklist as an extra file when you submit to a journal.

In your methods section, say that you used the SPIRITreporting guidelines, and cite them as:

Chan A-W, Tetzlaff JM, Gøtzsche PC, Altman DG, Mann H, Berlin J, Dickersin K, Hróbjartsson A, Schulz KF, Parulekar WR, Krleža-Jerić K, Laupacis A, Moher D. SPIRIT 2013 Explanation and Elaboration: Guidance for protocols of clinical trials. BMJ. 2013;346:e7586

		Reporting Item	Page Number
**Administrative information**			
Title	#1	Descriptive title identifying the study design, population, interventions, and, if applicable, trial acronym	1
Trial registration	#2a	Trial identifier and registry name. If not yet registered,	2

For peer review only

		name of intended registry	
Trial registration: data set	#2b	All items from the World Health Organization Trial Registration Data Set	n/a
Protocol version	#3	Date and version identifier	final
Funding	#4	Sources and types of financial, material, and other support	5; 19
Roles and responsibilities: contributorship	#5a	Names, affiliations, and roles of protocol contributors	19
Roles and responsibilities: sponsor contact information	#5b	Name and contact information for the trial sponsor	19
Roles and responsibilities: sponsor and funder	#5c	Role of study sponsor and funders, if any, in study design; collection, management, analysis, and interpretation of data; writing of the report; and the decision to submit the report for publication, including whether they will have ultimate authority over any of these activities	6-6
Roles and responsibilities: committees	#5d	Composition, roles, and responsibilities of the coordinating centre, steering committee, endpoint adjudication committee, data management team, and other individuals or groups overseeing the trial, if applicable (see Item 21a for data monitoring committee)	n/a

Introduction

For peer review only

Background and rationale	#6a	Description of research question and justification for undertaking the trial, including summary of relevant studies (published and unpublished) examining benefits and harms for each intervention	4-5; 8-10
Background and rationale: choice of comparators	#6b	Explanation for choice of comparators	n/a
Objectives	#7	Specific objectives or hypotheses	6; 8-10
Trial design	#8	Description of trial design including type of trial (eg, parallel group, crossover, factorial, single group), allocation ratio, and framework (eg, superiority, equivalence, non-inferiority, exploratory)	8
**Methods: Participants, interventions, and outcomes**			
Study setting	#9	Description of study settings (eg, community clinic, academic hospital) and list of countries where data will be collected. Reference to where list of study sites can be obtained	6
Eligibility criteria	#10	Inclusion and exclusion criteria for participants. If applicable, eligibility criteria for study centres and individuals who will perform the interventions (eg,	11-12

For peer review only

		surgeons, psychotherapists)	
Interventions: description	#11a	Interventions for each group with sufficient detail to allow replication, including how and when they will be administered	6-7
Interventions: modifications	#11b	Criteria for discontinuing or modifying allocated interventions for a given trial participant (eg, drug dose change in response to harms, participant request, or improving / worsening disease)	n/a
Interventions: adherance	#11c	Strategies to improve adherence to intervention protocols, and any procedures for monitoring adherence (eg, drug tablet return; laboratory tests)	n/a
Interventions: concomitant care	#11d	Relevant concomitant care and interventions that are permitted or prohibited during the trial	n/a
Outcomes	#12	Primary, secondary, and other outcomes, including the specific measurement variable (eg, systolic blood pressure), analysis metric (eg, change from baseline, final value, time to event), method of aggregation (eg, median, proportion), and time point for each outcome. Explanation of the clinical relevance of chosen efficacy and harm outcomes is strongly recommended	12-13
Participant timeline	#13	Time schedule of enrolment, interventions (including any run-ins and washouts), assessments, and visits for participants. A schematic diagram is highly recommended (see Figure)	10

For peer review only

Sample size	#14	Estimated number of participants needed to achieve study objectives and how it was determined, including clinical and statistical assumptions supporting any sample size calculations	11
Recruitment	#15	Strategies for achieving adequate participant enrolment to reach target sample size	11
**Methods: Assignment of interventions (for controlled trials)**			
Allocation: sequence generation	#16a	Method of generating the allocation sequence (eg, computer-generated random numbers), and list of any factors for stratification. To reduce predictability of a random sequence, details of any planned restriction (eg, blocking) should be provided in a separate document that is unavailable to those who enrol participants or assign interventions	n/a
Allocation concealment mechanism	#16b	Mechanism of implementing the allocation sequence (eg, central telephone; sequentially numbered, opaque, sealed envelopes), describing any steps to conceal the sequence until interventions are assigned	n/a
Allocation: implementation	#16c	Who will generate the allocation sequence, who will enrol participants, and who will assign participants to interventions	n/a

For peer review only

Blinding (masking)	#17a	Who will be blinded after assignment to interventions (eg, trial participants, care providers, outcome assessors, data analysts), and how	n/a
Blinding (masking): emergency unblinding	#17b	If blinded, circumstances under which unblinding is permissible, and procedure for revealing a participant's allocated intervention during the trial	n/a
**Methods: Data collection, management, and analysis**			
Data collection plan	#18a	Plans for assessment and collection of outcome, baseline, and other trial data, including any related processes to promote data quality (eg, duplicate measurements, training of assessors) and a description of study instruments (eg, questionnaires, laboratory tests) along with their reliability and validity, if known. Reference to where data collection forms can be found, if not in the protocol	12-13
Data collection plan: retention	#18b	Plans to promote participant retention and complete follow-up, including list of any outcome data to be collected for participants who discontinue or deviate from intervention protocols	n/a
Data management	#19	Plans for data entry, coding, security, and storage, including any related processes to promote data quality (eg, double data entry; range checks for data values).	14

For peer review only

		Reference to where details of data management procedures can be found, if not in the protocol	
Statistics: outcomes	#20a	Statistical methods for analysing primary and secondary outcomes. Reference to where other details of the statistical analysis plan can be found, if not in the protocol	13
Statistics: additional analyses	#20b	Methods for any additional analyses (eg, subgroup and adjusted analyses)	n/a
Statistics: analysis population and missing data	#20c	Definition of analysis population relating to protocol non-adherence (eg, as randomised analysis), and any statistical methods to handle missing data (eg, multiple imputation)	n/a
**Methods: Monitoring**			
Data monitoring: formal committee	#21a	Composition of data monitoring committee (DMC); summary of its role and reporting structure; statement of whether it is independent from the sponsor and competing interests; and reference to where further details about its charter can be found, if not in the protocol. Alternatively, an explanation of why a DMC is not needed	14
Data monitoring: interim analysis	#21b	Description of any interim analyses and stopping guidelines, including who will have access to these interim results and make the final decision to terminate the trial	n/a
Harms	#22	Plans for collecting, assessing, reporting, and managing solicited and spontaneously reported adverse events and other unintended effects of trial interventions or trial	n/a

For peer review only

		conduct	
Auditing	#23	Frequency and procedures for auditing trial conduct, if any, and whether the process will be independent from investigators and the sponsor	n/a
**Ethics and dissemination**			
Research ethics approval	#24	Plans for seeking research ethics committee / institutional review board (REC / IRB) approval	14
Protocol amendments	#25	Plans for communicating important protocol modifications (eg, changes to eligibility criteria, outcomes, analyses) to relevant parties (eg, investigators, REC / IRBs, trial participants, trial registries, journals, regulators)	n/a
Consent or assent	#26a	Who will obtain informed consent or assent from potential trial participants or authorised surrogates, and how (see Item 32)	14
Consent or assent: ancillary studies	#26b	Additional consent provisions for collection and use of participant data and biological specimens in ancillary studies, if applicable	n/a
Confidentiality	#27	How personal information about potential and enrolled participants will be collected, shared, and maintained in order to protect confidentiality before, during, and after the trial	14
Declaration of	#28	Financial and other competing interests for principal	19

For peer review only

interests		investigators for the overall trial and each study site	
Data access	#29	Statement of who will have access to the final trial dataset, and disclosure of contractual agreements that limit such access for investigators	14
Ancillary and post trial care	#30	Provisions, if any, for ancillary and post-trial care, and for compensation to those who suffer harm from trial participation	n/a
Dissemination policy: trial results	#31a	Plans for investigators and sponsor to communicate trial results to participants, healthcare professionals, the public, and other relevant groups (eg, via publication, reporting in results databases, or other data sharing arrangements), including any publication restrictions	14
Dissemination policy: authorship	#31b	Authorship eligibility guidelines and any intended use of professional writers	14
Dissemination policy: reproducible research	#31c	Plans, if any, for granting public access to the full protocol, participant-level dataset, and statistical code	n/a
**Appendices**			
Informed consent materials	#32	Model consent form and other related documentation given to participants and authorised surrogates	14
Biological specimens	#33	Plans for collection, laboratory evaluation, and storage of biological specimens for genetic or molecular analysis in the current trial and for future use in ancillary studies, if applicable	n/a

For peer review only

The SPIRIT Explanation and Elaboration paper is distributed under the terms of the Creative Commons Attribution License CC-BY-NC. This checklist was completed on 16. October 2023 using https://www.goodreports.org/, a tool made by the EQUATOR Network in collaboration with Penelope.ai

For peer review only

BMJ Open

Development, piloting, and evaluation of an app-supported psychosocial prevention intervention: A study protocol of a mixed-methods approach

Journal:	*BMJ Open*
Manuscript ID	bmjopen-2023-081390.R1
Article Type:	Protocol
Date Submitted by the Author:	05-Jan-2024
Complete List of Authors:	Stephan, Johannes; Technical University of Munich, TUM School of Medicine and Health, Chair of Social Determinants of Health<br>Gehrmann, Jan; Technical University of Munich, TUM School of Medicine and Health, Chair of Social Determinants of Health; Technical University of Munich, Institute of General Practice and Health Services Research<br>Stullich, Ananda; Technical University of Munich, TUM School of Medicine and Health, Chair of Social Determinants of Health<br>Hoffmann, Laura; Technical University of Munich, TUM School of Medicine and Health, Chair of Social Determinants of Health<br>Richter, Matthias; Technical University of Munich, TUM School of Medicine and Health, Chair of Social Determinants of Health<br>for the, PE³PP study group; for the PE³PP study group
**Primary Subject Heading**:	Health services research
Secondary Subject Heading:	Mental health, Public health
Keywords:	MENTAL HEALTH, PREVENTIVE MEDICINE, Burnout, QUALITATIVE RESEARCH, Quality of Life, Stress, Psychological

SCHOLARONE™
Manuscripts

Title

Development, piloting, and evaluation of an app-supported psychosocial prevention intervention: A study protocol of a mixed-methods approach

Authors:

Johannes Stephan*[1], Jan Gehrmann*[1,2], Ananda Stullich[1], Laura Hoffmann[1], Matthias Richter[1] for the PE³PP study group

Study group: Olaf Ballaschke, Sylvia Berlin, Florian Finke, Frank Gabel, Anastasia Grossmann, Claudia Gruhner, Kathleen Kaminski, Andreas Seemann (in alphabetical order)

* Both authors contributed equally to the manuscript and share the first authorship.

[1] Technical University of Munich, TUM School of Medicine and Health, Department Health and Sport Sciences, Chair of Social Determinants of Health

[2] Technical University of Munich, TUM School of Medicine and Health, Institute of General Practice and Health Services Research

Correspondence:

Johannes Stephan, Technical University of Munich, TUM School of Medicine and Health, Department Health and Sport Sciences, Chair of Social Determinants of Health, Georg-Brauchle-Ring 60/62, D-80922 Munich, Germany, Johannes.Stephan2@tum.de

For peer review only

Abstract

Introduction

Rates of incapacity to work due to mental disorders have increased in many European countries. The consequences of persistent stress can impact individuals' physical and psychological well-being and gradually develop into chronic stress. Mental disorders or symptoms of burnout syndrome can have severe consequences. Mental disorders leading to work incapacity significantly burden the health system. Prevention interventions can protect against burnout, depression, anxiety, and other mental health disorders. Digital health is a promising approach to increase the utilization of effective prevention interventions. This mixed-methods study evaluates a newly developed app-supported psychosocial prevention intervention called 'RV Fit Mental Health' to strengthen participation in working life.

Methods and analysis

The study uses a three-stage parallel mixed-methods design. This study accompanies the development (stage 1), piloting (stage 2), and evaluation (stage 3) of the new intervention.

For peer review only - http://bmjopen.bmj.com/site/about/guidelines.xhtml

Within the stages, there is a quantitative as well as a qualitative research strand. Employed persons with an incipient mental disorder will be included. Additionally, experts within the project or connected areas will be included. Quantitative data will be analyzed using multifactorial variance analyses in a pre-post design. Qualitative data will be analyzed using qualitative content analysis. The study is a comprehensive research approach to investigate the development, piloting, and evaluation of an app-supported psychosocial app-based prevention intervention. The rigor of the study will be achieved through data triangulation.

Ethics and dissemination

All participants will receive detailed study information and give written informed consent before data collection. Ethical approval was obtained from the Technical University of Munich Ethics Committee. All data collection will follow all legislative rules regarding data protection, also following the Declaration of Helsinki. The study results will be disseminated in peer-reviewed journals and presented at international conferences.

Trial registration number DRKS-ID: DRKS00030818 & DRKS00033080

Wordcount: 4.975 words

Keywords

mental health care, digital health, mixed methods, study protocol, prevention

Strengths and limitations of the study

- A mixed-methods research design is used for the entire study, which allows multiple perspectives to be focused on the same topic, leading to comprehensive results.
- The generalization of quantitative and qualitative results is challenging but offers various potentials for comparison and contrast regarding other innovative projects.
- The holistic approach integrating qualitative and quantitative data collection offers the potential to analyze the peculiarities of the digital dimension of the intervention.

For peer review only

1. Introduction

Over the past two decades, cases of incapacity to work due to mental disorders have increased in many European countries and represent a significant burden of disease [1, 2]. About one in five people have a current mental disorder (18% in Germany, 17.3% in the EU, 18.9% in the US, and 17.6% worldwide) [3–5]. In a study commissioned by the German health insurance company 'Techniker Krankenkasse' (TK), 47% of respondents reported that work, school, or study caused them to feel stressed. Participants stated that the main stressors were excessive workload, time pressure, rush, interruptions, and disruptions [6]. Hogan et al. described that long-term workplace stress is often caused by an imbalance between high work demands and the limited personal resources of workers [7]. The consequences of persistent work-related stress can impact individuals' physical and psychological well-being [8]. Excessive demands at work can gradually develop into chronic work stress, whereby symptoms of burnout syndrome can be consequences [8]. Long-term symptoms of burnout syndrome increase the risk of mental disorders such as sleep disorders, depressive symptoms, adjustment disorders, and physical health [8–10]. Employee performance, sick leave, absenteeism, accidents, and staff turnover are strongly influenced by mental health problems [9]. A relationship can also be shown between burnout and incapacity to work, as well as early entry into a disability pension [10].

A study by another German health insurance company, the 'Deutsche Angestellten Krankenkasse' (DAK), showed that the number of sick leave cases caused by mental health issues has almost tripled over the last 25 years [11]. The health report of the TK showed similar findings: The number of sick days due to mentally caused inability to work more than doubled from 2000 (129 sick days per 100 insurance years) to 2021 (283 sick days per 100 insurance years) in Germany. The main reasons for this increase were depressive episodes and reactions to severe stress and adjustment disorders [12]. Mental disorders cause the most days of incapacity to work at a stretch in Germany [11, 12]. Individuals who are permanently unable to work due to health reasons can apply for and receive a disability pension. The German Pension Insurance Association reports a significant increase, by double, in first-time granted disability pensions due to mental disorders in the last 25 years [13]. As a result, in the year 2021, mental disorders (41.7%) became the primary reason for disability pensions, followed by new formations such as cancer (15.3%), musculoskeletal disorders (11.5%), and cardiovascular diseases (9.1%) [13].

According to the OECD, in 2015, the costs of mental disorders in Germany accounted for approximately 4.8% of the Gross Domestic Product (GDP) [4]. Compared to the OECD-28 average, Germany's expenditures related to mental disorders are 0.7 percentage points above the average; Germany spends proportionally the second most on mental disorders in Europe [4].

For peer review only - http://bmjopen.bmj.com/site/about/guidelines.xhtml

The European Psychiatric Association states that various mental disorders can be prevented through evidence-based interventions by strengthening protective factors or reducing risk factors to promote mental health and disease prevention, e.g. through psychoeducation, skills training, stress management or other various therapeutic elements [14]. Consequently, psychosocial prevention interventions can protect against burnout, depression, anxiety, and other mental health disorders [8, 11, 15, 16]. Effective prevention interventions strengthen environmental and individual resources while reducing risk factors [17].

In Germany, the National Prevention Conference has therefore highlighted protecting and strengthening mental health in the workplace as a priority [18]. The German Pension Insurance supports this and acts according to the principle of 'prevention before rehabilitation' [19]. There is a general need to expand attractive evidence-based prevention interventions and improve access to and utilization of these services [20].

Determinants of the use of digital health applications

Digital Health is a promising approach to increase the utilization and quality of prevention interventions by using information and communication technologies to meet health-related needs [21]. Studies have shown that psychosocial aspects can be strengthened during inpatient treatment with the application of various therapies or elements such as individual or group training or psychoeducation [22–24]. Additionally, positive outcomes have been demonstrated for digitally supported mental health prevention interventions using a variety of therapeutic or psychoeducational elements [25–27]. Among the digitally supported prevention interventions that were not exclusively related to work, various outcomes were examined in employees, such as work stress [25], perceived stress [26], anxiety, and quality of life [27], which are either related to or exacerbated by the work environment. Digital health applications can be used flexibly in terms of time and location and can therefore be integrated into everyday life alongside work [25–27]. Individuals using digital health applications are exposed to various determinants influencing their effective and efficient use [28, 29]. One of the crucial determinants seems to be digital health literacy (DHL) [30]. DHL encompasses searching for and finding health information from digital sources, comprehending and assessing it, and applying acquired knowledge to address health-specific questions and solve problems [31]. It also encompasses skills and knowledge to interact productively with technology-enabled health tools [32, 33]. Other determinants that influence the utilization of digital health applications include cultural and social factors [32, 34, 35], motivation and interest [32, 33, 36, 37], and the accessibility and availability of technology [38–40]. Subsequently, there is evidence of a relationship between DHL and adherence to digital health interventions [41, 42].

For peer review only - http://bmjopen.bmj.com/site/about/guidelines.xhtml

When investigating the effectiveness of an app-supported psychosocial prevention intervention, it is crucial to collect data on the different determinants that affect utilizing a digital health application. By expanding concepts of DHL to include socio-cultural factors, motivation, interest, technology accessibility, and availability, a holistic assessment of DHL can be operationalized that incorporates the relevant determinants and allows predictions about the use of a health application.

Implementation and acceptance of digital health applications

The importance of analyzing the acceptance and framework conditions and contexts of digital health applications is emphasized regarding the use of digital health applications. Therefore, it is essential to account for organizational and contextual factors that impact the implementation of innovations in healthcare settings [43–45]. This is particularly important as it has been shown that evidence of effectiveness has not readily led to sustained implementation [46]. Especially in mental healthcare, the implementation of digital health applications poses various and unique challenges, highlighting the importance of evaluating the effectiveness and factors for successful implementation [45, 47]. Various studies highlight that it is necessary to analyze the interrelation between digital intervention and the healthcare context in which it is implemented [48, 49]. Thus, many digital mental health care interventions show better effects when paired with some therapeutic or doctoral support [50]. It is also highlighted that the perspective of health professionals as well as patients needs to be analyzed in the development to enable successful implementation [51–53]. Analyzing the implementation of a digital intervention needs to apply a comprehensive approach using quantitative and qualitative data. As the development, piloting, and evaluation is an iterative process, there is a need to analyze the different stages, anticipating the various perspectives involved, like users as well as healthcare professionals, but also focus on the contextual factors like organizational routines, established processes, and regulatory or policy aspects [48, 54].

Problem summary

In summary, the following problems were identified: First, stress experienced by individuals related to or exacerbated by the work environment can become chronic, negatively affecting their mental and physical well-being. Consequently, this places an increasing burden on the healthcare systems. Second, prevention interventions can protect against the chronification of stress; however, prevention interventions are rarely taken up in Germany. Third, digitally enabled prevention interventions can increase utilization. However, it cannot be assumed that different people can use digital health applications equally effectively. Fourth, there are measurement tools for assessing DHL, but existing measurement tools do not account for potential determinants that may impact the effective use of digital health applications. Fifth, regarding developing and implementing a new digital intervention, there is a need to analyze the acceptance and contextual factors.

For peer review only - http://bmjopen.bmj.com/site/about/guidelines.xhtml

The study aims to investigate the development, piloting, and evaluation of a psychosocial app-based prevention intervention using a comprehensive mixed methods approach. The intervention explicitly focused on mental health problems related to or exacerbated by the work environment, such as affective disorders, phobic and other anxiety disorders, adjustment disorders, somatoform disorders, and burnout. Its main aim is to improve the participation of those affected in their working life. The study protocol was developed using the SPIRIT checklist [55].

2. Methods and analysis

Study background

The project 'Development, piloting, and evaluation of an app-supported psychosocial prevention intervention' (German acronym PE³PP)" is funded under the federal program 'Innovative Ways to Participate in Working Life – rehapro' of the Federal Ministry of Labor and Social Affairs Germany. The project runs from October 2021 until September 2026 and is led by the German Pension Insurance Central Germany.

This study protocol outlines the planned scientific accompaniment for developing, piloting, and evaluating a psychosocial app-supported prevention intervention called 'RV Fit Mental Health' by the Chair of Social Determinants of Health of the Technical University of Munich (TUM). The intervention aims to promote and strengthen individuals' participation in work life. It is a prospective multicentric study. The two central German rehabilitation clinics, Median Klinik Bad Gottleuba and SRH Medinet Burgenlandklinik, will lead in developing and conducting the intervention. Further PE³PP project partners are the health insurance companies 'Allgemeine Ortskrankenkasse Sachsen-Anhalt' (AOK SAN) in the State of Saxony-Anhalt and 'Allgemeine Ortskrankenkasse PLUS' (AOK PLUS) in the states of Saxony and Thuringia.

Study intervention

Innovation of the intervention

The psychosocial prevention intervention 'RV Fit Mental Health' aims to improve existing prevention interventions of the German Pension Insurance. The secondary prevention intervention is conceptually innovative, focusing on the psychological, psychosomatic, and psychosocial aspects of work-related participation disorders. It is formally innovative in terms of its temporal and methodological design. The duration of the intervention will be 14 weeks. It commences with a two-week inpatient initial phase in a rehabilitation clinic and a 12-week app-supported training phase. Participants receive intensive therapeutic support during the inpatient and training phases. The proactive recruitment of participants by German Pension Insurance Central Germany and the two health insurances, AOK SAN and AOK Plus, is also promising.

For peer review only - http://bmjopen.bmj.com/site/about/guidelines.xhtml

The framework of the intervention

The study is multicentric and will be conducted simultaneously at two clinics. 1020 participants are expected to undergo the intervention from September 2023 to September 2026. Participants complete the two-week inpatient phase in small groups of ten participants per clinic. Each participant will be assigned a therapeutic support person for both intervention phases. During the inpatient phase, the participant and therapeutic support person collaboratively establish prevention goals, resulting in a therapy plan for the inpatient and training phases.

Modules of the intervention

The prevention intervention covers various topics, including psychological stabilization, self-care, finding meaning and purpose, coping with multiple stressors and increasing digitalization, achieving work-life balance, and providing 'first aid' on topics such as burnout, depression, anxiety, sleep disorders, and pain. In addition to psychoeducational elements, the intervention includes training in social and emotional competencies, professional and personal stress management, interaction, and communication training, as well as the provision of mindfulness-based and body-oriented therapeutic elements. These substantive topics will be consolidated into modules, conveyed, and practiced during the inpatient and training phases.

Didactical realization of the intervention

During the inpatient phase, therapists conduct the modules in groups. Therapists conducting the modules include psychosomatic physicians, nutritionists, sports and physiotherapists, and psychologists, each possessing diverse expertise in interventions using a digital health application. In this process, all participants attend most of the modules. Additionally, there will be specific modules tailored to the individual needs of the participants. The content of the modules is conveyed and trained through presentations, lectures, exercises, and self-development phases.

In the training phase, the participants could flexibly arrange their training in terms of time and location. The training phase will be supported by an app, creating a digital connection between the rehabilitation clinic and participants. The app will contain the training plan designed by the therapeutic support person to achieve the prevention goals. The training plan will include various modules for practice during this phase. The therapeutic support person assigned to the training phase is a psychologist trained in the administrative aspects of using the app.

The app provides various methods and formats for delivering module content, such as exercises, instructions, training, and seminars in video and audio formats. In addition, interaction and communication features, including group chats and video call functions, enable participants to engage

For peer review only - http://bmjopen.bmj.com/site/about/guidelines.xhtml

with their therapeutic support person and other participants. Consequently, the app supports and accompanies the transfer of knowledge and behaviors initiated during the inpatient phase into the participants' daily lives.

During the training phase, each participant will have a coaching session with their therapeutic support person every four weeks. Participants also confer with their therapeutic support person the level of encouragement they needed to consistently train in the individual modules, as outlined in the intervention plan, between coaching sessions. This method aims to increase and maintain commitment to participation in therapy. Through the coaching sessions, the therapeutic support person and the participant will (re)evaluate the progress toward achieving the prevention goals. Moreover, group sessions will take place during the training phase, where participants from the same group in the inpatient phase and a therapeutic support person will work on relevant intervention content and exchange experiences within the group. In the 14th week of the prevention intervention, each participant will have an individual concluding session with their therapeutic support person, ending the intervention.

Design, aims, and research questions

The study follows a parallel mixed-methods design integrating quantitative and qualitative data [56–58]. This approach offers the opportunity to highlight multiple perspectives on the same topic and consequently betters comprehension. Mixed-methods research has several methodological challenges, especially regarding study rigor, data triangulation, and integration of the various data [57–59]. Data triangulation is a methodological approach that will contribute to the validity of the results by linking qualitative and quantitative data, offering various potentials for analysis through the dynamic and emergent process of data collection and analysis [60–63]. This is especially beneficial in evaluation and implementation research [64].

The study consists of three stages in which various research questions are answered (Figure 1). In stage one, we will collect qualitative data. Stages two and three have a qualitative and quantitative research strand with corresponding objectives and research questions.

Figure 1: Overview of the study stages

Stage 1: Development (July 2022 – August 2023)

Stage one (S1) aims to scientifically accompany the development of the ‘RV Fit Mental Health’ intervention. The central research question is: What needs to be done to provide an attractive, innovative psychosocial prevention intervention?

For peer review only - http://bmjopen.bmj.com/site/about/guidelines.xhtml

In this stage, the different dimensions of the development process and the requirements for the organizational implementation are of interest. This stage consists of a qualitative research strand. The results thus have an evaluative character in that they enable the optimization and adaptation of the needs of the experts involved in planning and conducting the intervention and provide indications for successful cooperation between the project partners.

Qualitative research questions

We will answer the following sub-research questions:

- What are the needs and requirements of project partners with regard to the development of psychosocial intervention?
- Does the app fulfill the required functionality for the app-supported training phase?

Stage 2: Piloting (September 2023 – December 2023)

In stage two (S2), we aim to answer the central research question: How should the psychosocial prevention intervention 'RV Fit Mental Health' be implemented?

We will pilot the intervention and assess its feasibility. Thus, three research objectives emerged for stage two. First, the needs and experiences of the participants will be analyzed to identify standards for addressing potential participants and to optimize the intervention. Second, the constructed instrument for evaluating the intervention will undergo a pretest and a feasibility assessment. As the instrument will include an assessment of DHL, we aim to identify additional skills, competencies, and prerequisites that need to be analyzed to predict effectiveness. This results in five qualitative and one quantitative sub-research questions for stage two.

Qualitative research questions

- How should an app-supported prevention intervention be designed and organized to be feasible and accepted by the participants and the experts?
- What needs, expectations, and experiences were the first participants able to perceive during the initial run-through of the prevention intervention?
- What are facilitating factors and barriers regarding the implementation of the intervention?
- Which possibilities for optimization occur to increase the fit and acceptance of the prevention intervention, especially regarding the fit between the inpatient and the training phase?

For peer review only - http://bmjopen.bmj.com/site/about/guidelines.xhtml

- What competencies, skills, and prerequisites should users of digital health apps possess besides digital health literacy?

Quantitative research question

- Is the constructed survey instrument to measure primary and secondary outcomes feasible for participants?

Stage 3: Evaluation (January 2023 – April 2026)

Stage three (S3) aims to analyze the implementation and execution of the intervention. The key research questions in the evaluation stage are: Is the implementation of the psychosocial prevention intervention achieved? Is the psychosocial prevention intervention successful?

We will examine organizational conditions, accessibility of the target group, and collaboration among the project partners to implement the prevention intervention. We will investigate this from the perspectives of professionals and intervention participants. Additionally, we will assess the effectiveness of the intervention. This results in six qualitative and four quantitative sub-research questions for stage three.

Qualitative research questions

- Is the implementation of the psychosocial prevention intervention successful? What facilitating factors and barriers occurred?
- Is the app-supported prevention intervention feasible and accepted by the participants and the experts?
- What are the conditions for the implementing prevention intervention in terms of organizational processes and resources? What are the requirements from the health care professional's point of view?
- How is the dovetailing between the inpatient and the digital phase? What are the facilitating factors and barriers in the independent use of the app, and what are the determinants for successful therapeutic support in the digital phase?
- How does the cooperation and collaboration of the different partners develop during the project?
- What are the potentials, challenges, and needs for adaptation?

For peer review only - http://bmjopen.bmj.com/site/about/guidelines.xhtml

Quantitative research questions

- What effect does the intervention have on the participants' ability to work, as measured by the Work Ability Index (WAI)?
- What effect does the intervention have on quality of life as measured by the World Health Organization of Life-BREF (WHOQOL-BREF)?
- What effect does the intervention have on perceived stress levels as measured by the Depression Anxiety Stress Scale-21 (DASS-21)?
- Is there a correlation between the level of digital health literacy, measured with the G-eHEALS and a holistic DHL instrument, and post-intervention levels of work ability, quality of life, and perceived stress?

Intervention participants and study sampling

Participants in the prevention intervention

There are two pathways through which individuals are recruited for the prevention intervention. Pathway one is carried out by the German Pension Insurance Central Germany, a regional institution responsible for two million employed people and over one million pensioners. Pathway two is carried out by the health insurance companies AOK SAN and AOK PLUS, regional institutions of the Health Insurance AOK in the east of Germany, insuring a combined 4.3 million citizens. The three insurance companies will contact eligible insureds and proactively suggest the RV Fit Mental Health prevention intervention.

Sample and recruitment for all study stages

Qualitative Sample - Participants of the Intervention (S2, S3)

Focus groups (FG) with intervention participants will be conducted during the piloting (S2) and evaluation (S3) stages. We will sample through a random sampling method, where data collection time points will be predetermined. We plan four FG in the S2 with 6-10 participants each. Ten FG, also with 6-10 participants each, are planned in S3. Overall, we plan up to 14 FG with intervention participants. Contact with the individual group participants for the qualitative data collection is initiated via the respective clinic staff. If there is interest in participation, the clinics forward the time and number of participants. Inclusion criteria are the participation in the prevention intervention and sufficient German language skills.

For peer review only - http://bmjopen.bmj.com/site/about/guidelines.xhtml

Qualitative Sample - Experts (S1, S2, S3)

Expert interviews with representatives of the German Pension Insurance Central Germany, the AOK SAN, and AOK Plus, and with the executing healthcare professionals from the intervention clinics, will be conducted during the development (S1) and evaluation (S3) stages. There will also be FG in the pilot (S2) stage with these experts. Experts involved in planning and conducting the intervention are included in all stages. Inclusion criteria are the involvement in the development and/or implementation of the intervention, either organizationally or therapeutically. Up to ten participants will participate in each point of the data collection. The TUM project team will proactively recruit the participants of the expert interviews and the FG.

Furthermore, additional expert interviews will be conducted in S2. Included are healthcare professionals (this may include physicians, psychologists, and psychotherapists) who have prescribed or recommended app-based (digital) health applications for disease management, health promotion, or prevention as part of a treatment pathway, individuals who are involved in health research regarding the topic of digital health and digital health literacy as well as individuals from health policy in the field of digital health. Excluded are individuals who have prescribed, recommended or are experienced in other digital health technologies, such as wearables (smartwatches, trackers, etc.), and people who do not work in healthcare. We will interview ten to twelve experts. The experts will be recruited via professional associations and proactively through the study team.

Quantitative Sample - Participants of the Intervention (S2, S3)

We aim to survey all participants from the intervention's piloting (S2) and evaluation (S3) stages. We will use a non-probability sample for recruitment. Participants in the prevention intervention will receive a flyer on recruitment for our study via the German Pension Insurance and the intervention clinics. On the flyer are instructions and online access for our quantitative data collection. In S2, we expect a total of 60 participants. In S3, we intend to include 960 participants for the pre-post-design effectiveness assessment. Inclusion criteria are the participation in the prevention intervention and sufficient German language skills. We expect dropouts, especially during the digital training phase.

Data collection

Qualitative data collection

The overall qualitative data collection is depicted graphically in Figure 2.

Figure 2: Overview of qualitative data collection

For peer review only - http://bmjopen.bmj.com/site/about/guidelines.xhtml

The FG with participants occurs both during the initial inpatient phase on-site at the clinics and online during the digital training phase. The FG in the inpatient phase occurs towards the end of the inpatient stay. The online FG takes place when at least four weeks of the digital training phase have been completed.

The interviews with the experts will be conducted online. Depending on the research questions in each stage, a semi-structured interview guide will be developed and pretested for each expert group. The interview guides will be developed following the principles of Helfferich [65]. Furthermore, we are following the methodical principles of expert interviews and recognize the context of the new intervention as well as the evaluative character of the expert interviews [66–68].

The FG with the experts will take place during a project workshop on-site. The FG is based on a purposive moderation guide starting with a stimulus recognizing the specified topics, the organizational contexts, and the environment of the intervention [69].

Quantitative data collection

Quantitative data will be collected from the study participants in S2 and S3 at four different points (t0-t3) through (online) surveys, depicted graphically in Figure 3.

- t0 (first survey) transpires when insured persons are confirmed that participation in the prevention intervention is possible.
- t1 (second survey) takes place at the beginning of the prevention intervention within the first two days of the initial inpatient phase.
- t2 (third survey) is conducted two weeks after t1, at the end of the initial inpatient phase.
- t3 (fourth survey) occurs 12 weeks after the inpatient phase at the end of the 12-week training phase.

Figure 3: Overview of quantitative data collection

The primary outcomes of interest are workability, assessed using the German Work Ability Index [70]; quality of life, using the German WHOQOL-BREF [71]; stress levels, evaluated using the DASS-21 [72]; and eHealth literacy, which will be assessed using the G-eHEALS [73, 74], and a self-developed instrument for holistic DHL.

The secondary outcomes are anxiety and depressive symptoms (DASS-21) and self-efficacy using the SWOP K9 [75]. In addition, we are interested in health behavior as a secondary outcome, which we operationalize as alcohol consumption, smoking, physical activity, and dietary behavior. The training phase behavioral data follow the FITT criteria [76, 77]: frequency, intensity, time, and type. We will use FITT data to examine participants' adherence to the eHealth application [78]. Additionally,

For peer review only - http://bmjopen.bmj.com/site/about/guidelines.xhtml
For peer review only

sociodemographic data will be collected during survey t1, and behavioral data from the training phase will be gathered during survey t3.

Data analysis

Qualitative analysis

The semi-structured interviews and the FG will be conducted by the research team (JG, JS), audiotaped, verbatim transcribed, and analyzed following Content Analysis, using MAXQDA 2022 [79, 80]. Qualitative content analysis is a systematic, rule-bound approach orientated towards rules of text analysis laid down in advance and contributes to the intersubjectivity of the analysis procedure regarding the defined interests [80]. The analytical steps taken by the category system are, firstly, the summary and the inductive category formation, then secondly by the explication and the context analysis, finalized by the structuring and the deductive category formation. The category system will then be re-checked by applying it to the material, the research questions, and the case itself [72]. Constant exchange within the study team and the working group for Qualitative Research at the Chair for Social Determinants of Health will ensure the rigor and integrity of the analysis. The analysis will be framed by applying content-analytical quality criteria and reported using the consolidated criteria for reporting qualitative research (COREQ) [73].

Quantitative analysis

Quantitative data analysis will be conducted using the IBM *Statistical Package for Social Sciences* (SPSS) software. During S2, the dataset will undergo a preliminary examination for meaningfulness. This involves checking for the data's completeness, outliers, and plausibility. A descriptive characterization of the quantitative S2 sample will be performed based on sociodemographic data to gain initial insights into the sample.

For S3, we plan a pre-post design effectiveness evaluation. The baseline data will be compared with the data from t1-t3. For this, we will conduct multifactorial variance analyses to investigate whether there are differences in the groups of individual intervention time points regarding the primary and secondary outcomes, thus demonstrating the potential effect of the prevention intervention. Additionally, we will perform regression analyses to interpret the relationship between the collected variables, allowing for statements about the influence of individual variables on different outcomes. Similarly, we present a differentiated sample characterization for S3. In addition, we add a waitlist-controlled component. After collecting data from t0 to t4, we divide participants of S3 into an intervention and waitlist group. We conduct t-tests within each group to analyze effects between t0 and t1 for the waitlist group and between t1 and t3 for the intervention group. Calculating Cohen's d

For peer review only - http://bmjopen.bmj.com/site/about/guidelines.xhtml

helps us quantify effect sizes from these tests. We then use a Z-test to compare Cohen's d values of both groups to determine if there are significant differences in effect sizes.

Patient and public involvement

None.

3. Ethics and dissemination

Ethical approval was obtained from the Ethics Committee at the TUM School of Medicine and Health (2022-523-S-SR; 2023-316-S-SB). Following the Declaration of Helsinki, we will conduct the study according to ethical research principles, including the primacy of participant welfare, informed consent, scientific validity, review by an ethics committee, appropriate risk-benefit balance, protection of vulnerable groups, data security and confidentiality, transparent reporting, and voluntary participation. In the inpatient phase, therapists inform participants about the potential disadvantages of app use for the training phase and discuss strategies to mitigate these issues. During the coaching sessions in the training phase, the therapeutic support person systematically identifies any possible disadvantages of using the app. If any study participant decides not to participate or not continue the study, they may withdraw consent at any time without any consequences. All data management follows the requirements of the German version of the General Data Protection Regulation and is approved by the TUM internal department for data security. All data collected in the study will be pseudonymized or, if possible, anonymized. The pseudonymization through a code ensures data protection of all data. All data containing identificatory characteristics (e.g., record, pseudonym assignment list, etc.) is secured separately from study data. They are stored in locked rooms at the Chair of Social Determinants of Health. We will delete all data once the study has been completed, but at the latest, after the given legal restriction of 10 years. In case of withdrawal, all data will be deleted entirely or, in extraordinary cases, evaluated carefully anonymized. Deleting data from already analyzed or published data is not possible. We will publish all analyzed data without any possibility of identifying persons. All results of the quantitative analysis will be published anonymously, and the results of the qualitative analysis will be pseudonymized. The study results will be disseminated in international peer-reviewed journals and presented at international conferences.

4. Authors´ contributions

JG and JS wrote the initial manuscript draft, will collect the quantitative and qualitative data, and are responsible for data analyses. LH and MR were involved in the grant application. JS and JG wrote the study protocol for the Ethics Review Committee. LH, AS, and MR critically reviewed the article several times and counseled the entire process of this study protocol and research. All authors have read and approved the final version of the manuscript.

For peer review only - http://bmjopen.bmj.com/site/about/guidelines.xhtml

5. Funding statement

This study is supported by the Federal Ministry of Labour and Social Affairs as part of the rehapro funding program and is led by the German Pension Insurance of Central Germany (662S0033X1).

6. Competing interests statement

The authors of this study declare no conflicts of interest.

7. References

REFERENCES

1 Hildebrandt-Heene S, Dehl T, Zich K, et al. Gesundheitsreport 2023: Analyse der Arbeitsunfähigkeiten. Gesundheitsrisiko Personalmangel: Arbeitswelt unter Druck. Heidelberg: medhochzwei Verlag GmbH 2023.
2 Salomonsson S, Hedman-Lagerlöf E, Öst L-G. Sickness absence: a systematic review and meta-analysis of psychological treatments for individuals on sick leave due to common mental disorders. *Psychol Med* 2018;48(12):1954–65. doi:10.1017/S0033291718000065 [published Online First: 30 January 2018].
3 Müller G, Bombana M, Heinzel-Gutenbrenner M, et al. Socio-economic consequences of mental distress: quantifying the impact of self-reported mental distress on the days of incapacity to work and medical costs in a two-year period: a longitudinal study in Germany. *BMC Public Health* 2021;21(1):625. doi:10.1186/s12889-021-10637-8 [published Online First: 31 March 2021].
4 OECD. Health at a Glance: Europe 2018. Brussels: OECD 2018.
5 Steel Z, Marnane C, Iranpour C, et al. The global prevalence of common mental disorders: a systematic review and meta-analysis 1980-2013. *Int J Epidemiol* 2014;43(2):476–93. doi:10.1093/ije/dyu038 [published Online First: 19 March 2014].
6 Meyer B, Zill A, Dibla D. Entspann dich, Deutschland!: TK Stressstudie 2021. Hamburg: Technicker Krankenkasse 2021.
7 Hogan L-M, Björklund Carlstedt A, Wagman P. Occupational therapy and stress-related exhaustion - a scoping review. *Scand J Occup Ther* 2023:1–17. doi:10.1080/11038128.2023.2207802 [published Online First: 8 June 2023].
8 Edú-Valsania S, Laguía A, Moriano JA. Burnout: A Review of Theory and Measurement. *Int J Environ Res Public Health* 2022;19(3). doi:10.3390/ijerph19031780 [published Online First: 4 February 2022].
9 Wilken U, Breucker G. Mental health in the workplace: Situation analysis Germany. Geneva: International Labour Organization 2000.
10 Ahola K, Gould R, Virtanen M, et al. Occupational burnout as a predictor of disability pension: a population-based cohort study. *Occup Environ Med* 2009;66(5):284-90; discussion 282-3. doi:10.1136/oem.2008.038935 [published Online First: 18 November 2008].
11 Marschall J, Hildebrandt S, Nolting H-D. DAK-Gesundheitsreport 2019 2019. https://www.dak.de/dak/download/dak-gesundheitsreport-2019-sucht-pdf-2073718.pdf.
12 Grobe T, Bessel S. Gesundheitsreport 2022 Arbeitsunfähigkeit. Göttingen: aQua – Institut für angewandte Qualitätsförderung und Forschung im Gesundheitswesen GmbH 2022.
13 German Pension Insurance Association. Erwerbsminderungsrenten im Zeitablauf 2022 2022. Available at: https://www.deutsche-

For peer review only - http://bmjopen.bmj.com/site/about/guidelines.xhtml

rentenversicherung.de/DRV/DE/Experten/Zahlen-und-Fakten/Statistiken-und-Berichte/statistiken-und-berichte_node.html Accessed July 27, 2023.
14 Campion J, Bhui K, Bhugra D. European Psychiatric Association (EPA) guidance on prevention of mental disorders. *Eur Psychiatry* 2012;27(2):68–80. doi:10.1016/j.eurpsy.2011.10.004 [published Online First: 28 January 2012].
15 Lehofer M, Glehr R, Haring C, et al. Burnout & Depression. Ein Leitfaden zur Prävention, Früherkennung und Behandlung. Experten-Statement [Burnout and Depression. A Guideline for Prevention, Early Diagnosis, and Intervention. Experts Statement] 2011.
16 Singh V, Kumar A, Gupta S. Mental Health Prevention and Promotion-A Narrative Review. *Front Psychiatry* 2022;13:898009. doi:10.3389/fpsyt.2022.898009 [published Online First: 26 July 2022].
17 World Health Organization. Mental Health 2022. Available at: https://www.who.int/news-room/fact-sheets/detail/mental-health-strengthening-our-response Accessed July 25, 2023.
18 National Prevention Conference. Spezielle Ziele für die Zielgruppe Erwerbstätige 2022. Available at: https://www.npk-info.de/praeventionsstrategie/bundesrahmenempfehlungen/spezielle-ziele-fuer-die-zielgruppe-erwerbstaetige Accessed July 26, 2023.
19 German Pension Insurance Association. Reha-Bericht 2021. Berlin: H. Heenemann GmbH & Co. KG 2021.
20 Robert Koch-Institut. Wie steht es um Prävention und Gesundheitsförderung?: RKI-Bib1 (Robert Koch-Institut) 2015.
21 World Health Organization. Global strategy on digital health 2020-2025. Geneve: World Health Organization 2021.
22 Booth S, Ryan R, Clow A, et al. Enhancing well-being and building resilience in people living with cancer part 2: a central role for nurses. *Cancer Nursing Practice* 2019;18(1):42–50.
23 Goldbach N, Reif A, Preuss H, et al. A randomized trial of group therapy for resource activation for patients with affective disorder. *Journal of Psychotherapy Integration* 2023;33(1):1–19.
24 Breuer K, John SM, Finkeldey F, et al. Tertiary individual prevention improves mental health in patients with severe occupational hand eczema. *J Eur Acad Dermatol Venereol* 2015;29(9):1724–31. doi:10.1111/jdv.12975 [published Online First: 4 February 2015].
25 Bostock S, Crosswell AD, Prather AA, et al. Mindfulness on-the-go: Effects of a mindfulness meditation app on work stress and well-being. *J Occup Health Psychol* 2019;24(1):127–38.
26 Champion L, Economides M, Chandler C. The efficacy of a brief app-based mindfulness intervention on psychosocial outcomes in healthy adults: A pilot randomised controlled trial. *PLoS One* 2018;13(12):e0209482.
27 Zhou T, Li X, Pei Y, et al. Internet-based cognitive behavioural therapy for subthreshold depression: a systematic review and meta-analysis. *BMC Psychiatry* 2016;16(1):356.
28 Li J, Theng Y-L, Foo S. Predictors of online health information seeking behavior: Changes between 2002 and 2012. *Health Informatics J* 2016;22(4):804–14. doi:10.1177/1460458215595851 [published Online First: 10 August 2015].
29 Perich T, Andriessen K. Predictors of digital technology-based mental health programs in young adults for mental health support. *Health Promot Int* 2023;38(3).

For peer review only - http://bmjopen.bmj.com/site/about/guidelines.xhtml

30 van Kessel R, Wong BLH, Clemens T, et al. Digital health literacy as a super determinant of health: More than simply the sum of its parts. *Internet Interv* 2022;27:100500. doi:10.1016/j.invent.2022.100500 [published Online First: 7 February 2022].
31 Norman CD, Skinner HA. eHealth Literacy: Essential Skills for Consumer Health in a Networked World. *J Med Internet Res* 2006;8(2):e9. doi:10.2196/jmir.8.2.e9 [published Online First: 16 June 2006].
32 Sifat MS, Saperstein SL, Tasnim N, et al. Motivations Toward Using Digital Health and Exploring the Possibility of Using Digital Health for Mental Health in Bangladesh University Students: Cross-sectional Questionnaire Study. *JMIR Form Res* 2022;6(3):e34901. doi:10.2196/34901 [published Online First: 4 March 2022].
33 Dias N, Yamamoto R, Faulkner KG. Preferences and motivational factors for the use of digital technology in real world oncology studies. *JCO* 2020;38(15_suppl):e14119-e14119.
34 Ehrari H, Tordrup L, Müller S. The Digital Divide in Healthcare: A Socio-Cultural Perspective of Digital Literacy. Proceedings of the 55th Hawaii International Conference on System Sciences 2022 2022.
35 Hsieh JJP-A, Rai A, Keil M. Addressing Digital Inequality for the Socioeconomically Disadvantaged Through Government Initiatives: Forms of Capital That Affect ICT Utilization. *Information Systems Research* 2011;22(2):233–53.
36 Kaihlanen A-M, Virtanen L, Buchert U, et al. Towards digital health equity - a qualitative study of the challenges experienced by vulnerable groups in using digital health services in the COVID-19 era. *BMC Health Serv Res* 2022;22(1):188. doi:10.1186/s12913-022-07584-4 [published Online First: 12 February 2022].
37 Park DY, Kim H. Determinants of Intentions to Use Digital Mental Healthcare Content among University Students, Faculty, and Staff: Motivation, Perceived Usefulness, Perceived Ease of Use, and Parasocial Interaction with AI Chatbot. *Sustainability* 2023;15(1):872.
38 World Health Organization. Equity within digital health technology within the WHO European Region: a scoping review. Copenhagen: WHO Regional Office for Europe 2022.
39 Sieck CJ, Sheon A, Ancker JS, et al. Digital inclusion as a social determinant of health. *NPJ Digit Med* 2021;4(1):52. doi:10.1038/s41746-021-00413-8 [published Online First: 17 March 2021].
40 Richardson S, Lawrence K, Schoenthaler AM, et al. A framework for digital health equity. *NPJ Digit Med* 2022;5(1):119. doi:10.1038/s41746-022-00663-0 [published Online First: 18 August 2022].
41 Lu X, Zhang R. Association Between eHealth Literacy in Online Health Communities and Patient Adherence: Cross-sectional Questionnaire Study. *J Med Internet Res* 2021;23(9):e14908. doi:10.2196/14908 [published Online First: 13 September 2021].
42 Do BN, Tran TV, Phan DT, et al. Health Literacy, eHealth Literacy, Adherence to Infection Prevention and Control Procedures, Lifestyle Changes, and Suspected COVID-19 Symptoms Among Health Care Workers During Lockdown: Online Survey. *J Med Internet Res* 2020;22(11):e22894. doi:10.2196/22894 [published Online First: 12 November 2020].
43 Powell BJ, McMillen JC, Proctor EK, et al. A compilation of strategies for implementing clinical innovations in health and mental health. *Med Care Res Rev* 2012;69(2):123–57. doi:10.1177/1077558711430690 [published Online First: 26 December 2011].
44 Powell BJ, Waltz TJ, Chinman MJ, et al. A refined compilation of implementation strategies: results from the Expert Recommendations for Implementing Change (ERIC)

For peer review only

project. *Implement Sci* 2015;10:21. doi:10.1186/s13012-015-0209-1 [published Online First: 12 February 2015].
45 Graham AK, Lattie EG, Powell BJ, et al. Implementation strategies for digital mental health interventions in health care settings. *Am Psychol* 2020;75(8):1080–92.
46 Mohr DC, Weingardt KR, Reddy M, et al. Three Problems With Current Digital Mental Health Research . and Three Things We Can Do About Them. *Psychiatr Serv* 2017;68(5):427–29. doi:10.1176/appi.ps.201600541 [published Online First: 17 April 2017].
47 Mohr DC, Lyon AR, Lattie EG, et al. Accelerating Digital Mental Health Research From Early Design and Creation to Successful Implementation and Sustainment. *J Med Internet Res* 2017;19(5):e153. doi:10.2196/jmir.7725 [published Online First: 10 May 2017].
48 Shaw J, Agarwal P, Desveaux L, et al. Beyond "implementation": digital health innovation and service design. *npj Digital Med* 2018;1(1):48. doi:10.1038/s41746-018-0059-8 [published Online First: 20 September 2018].
49 Marwaha JS, Landman AB, Brat GA, et al. Deploying digital health tools within large, complex health systems: key considerations for adoption and implementation. *npj Digit. Med.* 2022;5(1):13. doi:10.1038/s41746-022-00557-1 [published Online First: 27 January 2022].
50 Lattie EG, Stiles-Shields C, Graham AK. An overview of and recommendations for more accessible digital mental health services. *Nat Rev Psychol* 2022;1(2):87–100. https://www.nature.com/articles/s44159-021-00003-1.
51 Mendes-Santos C, Nunes F, Weiderpass E, et al. Understanding Mental Health Professionals' Perspectives and Practices Regarding the Implementation of Digital Mental Health: Qualitative Study. *JMIR Form Res* 2022;6(4):e32558. doi:10.2196/32558 [published Online First: 12 April 2022].
52 Lukka L, Karhulahti V-M, Palva JM. Factors Affecting Digital Tool Use in Client Interaction According to Mental Health Professionals: Interview Study. *JMIR Hum Factors* 2023;10:e44681. doi:10.2196/44681 [published Online First: 10 July 2023].
53 Birnbaum F, Lewis D, Rosen RK, et al. Patient engagement and the design of digital health. *Acad Emerg Med* 2015;22(6):754–56. doi:10.1111/acem.12692 [published Online First: 21 May 2015].
54 Desveaux L, Soobiah C, Bhatia RS, et al. Identifying and Overcoming Policy-Level Barriers to the Implementation of Digital Health Innovation: Qualitative Study. *J Med Internet Res* 2019;21(12):e14994. doi:10.2196/14994 [published Online First: 20 December 2019].
55 Chan AW, Tetzlaff JM, Altman DG, et al. SPIRIT 2013 statement: defining standard protocol items for clinical trials. *Ann Intern Med* 2013;158(3):200–07.
56 Tariq S, Woodman J. Using mixed methods in health research. *JRSM Short Rep* 2013;4(6):2042533313479197. doi:10.1177/2042533313479197 [published Online First: 7 May 2013].
57 Kuckartz U. Mixed Methods: Methodologie, Forschungsdesigns und Analyseverfahren: Springer Fachmedien Wiesbaden 2014.
58 Fetters MD, Curry LA, Creswell JW. Achieving integration in mixed methods designs-principles and practices. *Health Services Research* 2013;48(6 Pt 2):2134–56. doi:10.1111/1475-6773.12117 [published Online First: 23 October 2013].
59 Wisdom JP, Cavaleri MA, Onwuegbuzie AJ, et al. Methodological reporting in qualitative, quantitative, and mixed methods health services research articles. *Health Services Research* 2012;47(2):721–45. doi:10.1111/j.1475-6773.2011.01344.x [published Online First: 8 November 2011].

For peer review only - http://bmjopen.bmj.com/site/about/guidelines.xhtml

60 Casey D, Murphy K. Issues in using methodological triangulation in research. *Nurse Researcher* 2009;16(4):40–55.
61 Farmer T, Robinson K, Elliott SJ, et al. Developing and implementing a triangulation protocol for qualitative health research. *Qualitative Health Research* 2006;16(3):377–94.
62 Gibson CB. Elaboration, Generalization, Triangulation, and Interpretation. *Organizational Research Methods* 2017;20(2):193–223.
63 Jick TD. Mixing Qualitative and Quantitative Methods: Triangulation in Action. *Administrative Science Quarterly* 1979;24(4):602.
64 Green CA, Duan N, Gibbons RD, et al. Approaches to Mixed Methods Dissemination and Implementation Research: Methods, Strengths, Caveats, and Opportunities. *Adm Policy Ment Health* 2015;42(5):508–23.
65 Helfferich C. Die Qualität qualitativer Daten: Manual für die Durchführung qualitativer Interviews, 4th edn. Wiesbaden: VS Verlag 2011.
66 Meuser M, Nagel U. The Expert Interview and Changes in Knowledge Production. In: Interviewing Experts: Palgrave Macmillan, London 2009:17–42.
67 Döringer S. 'The problem-centred expert interview'. Combining qualitative interviewing approaches for investigating implicit expert knowledge. *International Journal of Social Research Methodology* 2021;24(3):265–78.
68 Wroblewski A, Leitner A. Between Scientific Standards and Claims to Efficiency: Expert Interviews in Programme Evaluation. In: Interviewing Experts: Palgrave Macmillan, London 2009:235–51.
69 Smithson J. Using and analysing focus groups: Limitations and possibilities. *International Journal of Social Research Methodology* 2000;3(2):103–19.
70 Freyer M. Eine Konstruktvalidierung des Work Ability Index anhand einer repräsentativen Stichprobe von Erwerbstätigen in Deutschland: Bundesanstalt für Arbeitsschutz und Arbeitsmedizin (BAuA) 2019.
71 World Health Organization. WHOQOL. User Manual. Geneva: World Health Organization 2012.
72 Nilges P, Essau C. DASS. Depressions-Angst-Stress-Skalen - deutschsprachige Kurzfassung: ZPID (Leibniz Institute for Psychology) – Open Test Archive 2021.
73 Norman CD, Skinner HA. eHEALS: The eHealth Literacy Scale. *J Med Internet Res* 2006;8(4):e27. doi:10.2196/jmir.8.4.e27 [published Online First: 14 November 2006].
74 Soellner R, Huber S, Reder M. The Concept of eHealth Literacy and Its Measurement. *Journal of Media Psychology* 2014;26(1):29–38.
75 Scholler G, Fliege H, Klapp BF. SWOP-K9 - Fragebogen zu Selbstwirksamkeit-Optimismus-Pessimismus Kurzform: ZPID (Leibniz Institute for Psychology Information) – Testarchiv 1999.
76 Barisic A, Leatherdale ST, Kreiger N. Importance of frequency, intensity, time and type (FITT) in physical activity assessment for epidemiological research. *Can J Public Health* 2011;102(3):174–75.
77 Short CE, DeSmet A, Woods C, et al. Measuring Engagement in eHealth and mHealth Behavior Change Interventions: Viewpoint of Methodologies. *J Med Internet Res* 2018;20(11):e292. doi:10.2196/jmir.9397 [published Online First: 16 November 2018].
78 Sieverink F, Kelders SM, van Gemert-Pijnen JE. Clarifying the Concept of Adherence to eHealth Technology: Systematic Review on When Usage Becomes Adherence. *J Med Internet Res* 2017;19(12):e402. doi:10.2196/jmir.8578 [published Online First: 6 December 2017].
79 Mayring P. Qualitative Content Analysis. *Forum qualitative Sozialforschung* 2000;1(2).

For peer review only - http://bmjopen.bmj.com/site/about/guidelines.xhtml

80 Mayring P. Qualitative content analysis: theoretical foundation, basic procedures and software solution. Klagenfurt 2014.

For peer review only

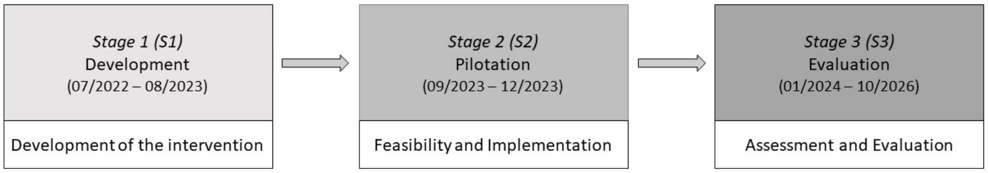

Figure1: Overview of the study stages

338x190mm (96 x 96 DPI)

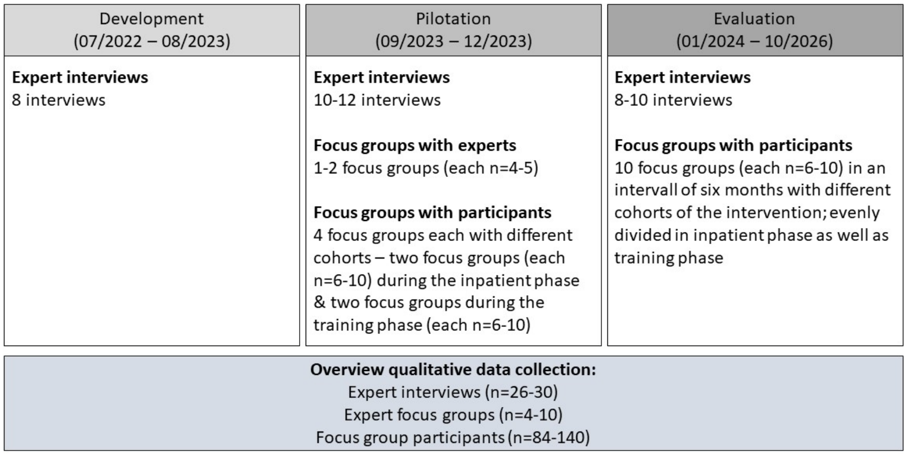

Figure2: Overview of qualitative data collection

338x190mm (96 x 96 DPI)

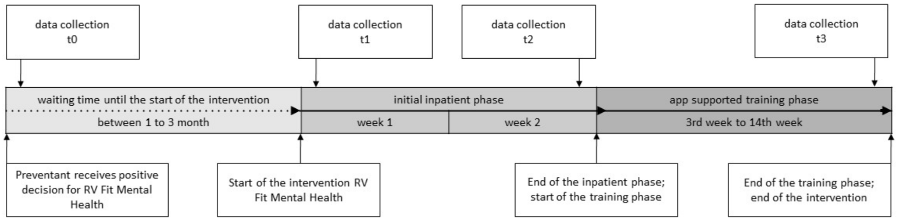

Figure 3: Overview of quantitative data collection

338x190mm (96 x 96 DPI)

Probandeninformation und Einwilligungserklärung zum Forschungsvorhaben: Erprobung und Evaluierung einer psychosozialen Präventionsleistung im Rahmen des Projektes PE³PP - Fokusgruppeninterviews

Probandeninformation

Sehr geehrte Teilnehmerin, sehr geehrter Teilnehmer,

wir bitten Sie, an einer wissenschaftlichen Studie teilzunehmen. In dieser Probandeninformation finden Sie alles Wesentliche zu der Studie.

Bitte lesen Sie diese Information sorgfältig durch. Wir werden zusätzlich mit Ihnen über die Studie sprechen und Ihre Fragen beantworten.

Pro Fokusgruppe werden 7-10 Teilnehmer:innen eingeschlossen. Insgesamt sollen über den Projektverlauf zwölf Fokusgruppen durchgeführt werden, sodass sich eine Teilnehmendenzahl von insgesamt 84 bis zu 120 Teilnehmer:innen ergibt.

Diese Studie wird vom Lehrstuhl für Soziale Determinanten der Gesundheit, Fakultät für Sport- und Gesundheitswissenschaften an der Technischen Universität München geplant und durchgeführt.

Sie wird durch öffentliche Mittel (Bundesministerium für Arbeit und Soziales) gefördert.

Die Studie wurde der zuständigen Ethikkommission vorgelegt. Sie hat keine Einwände erhoben.

Die Teilnahme an der Studie ist freiwillig. Wenn Sie nicht teilnehmen wollen oder wenn Sie später Ihre Einwilligung widerrufen, werden Ihnen daraus keine Nachteile entstehen.

Wenn Sie jetzt oder auch später weitere Fragen zur Studie haben, können Sie uns gerne kontaktieren. Wenden Sie sich dazu an Johannes Stephan, Georg-Brauchle-Ring 60/62, 80992 München, johannes.stephan2@tum.de.

Warum wird diese Studie durchgeführt?

Die Präventionsleistung RV Fit Psychische Gesundheit, an der Sie teilnehmen, ist ein neues Angebot der Deutschen Rentenversicherung Mitteldeutschland. Da es ein neues Angebot ist, soll untersucht werden, wie die Präventionsleistung aus Sicht der Teilnehmenden, also aus Ihrer Sicht, umgesetzt werden sollte. Es sollen Erkenntnisse darüber gewonnen werden, welche Erwartungen und Erfahrungen Sie bezüglich der Präventionsleistung haben. Ein weiteres Ziel der Fokusgruppeninterviews ist es, Punkte, durch die die Präventionsleistung verbessert werden kann, zu identifizieren. Die Ergebnisse dieser Untersuchung sollen dann im konkreten dazu genutzt werden die Präventionsleistung stetig zu verbessern und auf Basis Ihrer Erwartungen und Erfahrungen die Präventionsleistung auch für zukünftige Teilnehmer:inenn von RV Fit Psychische Gesundheit optimal gestalten zu können.

Wie läuft die Studie ab?

Zu Beginn des vereinbarten Termins geben wir Ihnen die Möglichkeit, aufkommende Fragen und Anmerkungen hinsichtlich der Einwilligungserklärung zu stellen. Nachdem Sie informiert

For peer review only

For peer review only - http://bmjopen.bmj.com/site/about/guidelines.xhtml

wurden, bitten wir Sie die Einwilligungserklärung zu unterschreiben. Die unterschriebene Einwilligungserklärung können Sie dann entweder unmittelbar vor Ort, im Nachgang via E-Mail oder auch postalisch an die Forschenden übersenden. Die Fokusgruppen basieren auf einem wissenschaftlich konzipierten Leitfaden inklusive eines Erzählanreizes, der dazu dient den Austausch in der Gruppe zu initiieren. Die Fokusgruppe dauert zwischen 60 und 90 Minuten und wird aufgezeichnet. Die Fokusgruppe findet entweder in Präsenz vor Ort in den Kliniken oder online mittels einer Videokonferenz statt. Bei den Präsenzfokusgruppen erfolgt die Aufnahme mittels zweier im Raum verteilter Aufnahmegeräten. Die Präsenzfokusgruppen finden in entsprechenden passenden und geschützten Räumen in den Örtlichkeiten der Kliniken vor Ort statt. Die Onlinefokusgruppen finden über das Videokonferenztool Zoom statt. Die Tonaufnahme wird dabei anhand eines zusätzlich in das Zoom Meeting eingeloggten Computers sichergestellt, auf welchem über ein Aufnahmeprogramm aufgezeichnet wird.

Gibt es einen persönlichen Nutzen durch die Teilnahme an der Studie?

Durch die Teilnahme an den Fokusgruppeninterviews können Sie ihre Erfahrungen und Sichtweisen auf die absolvierte Präventionsleistung teilen und damit die Gestaltung und den Ablauf der Präventionsleistung mitgestalten. Ihre Perspektiven werden als wissenschaftliche fundiertes Feedback auch in das Projektteam rückgespiegelt.

Weiterhin ist es allerdings möglich, dass Sie durch Ihre Teilnahme keinen direkten Nutzen haben. Die Ergebnisse der Studie können jedoch in Zukunft anderen Menschen helfen.

Welche Risiken sind mit einer Teilnahme an der Studie verbunden?

Die Teilnahme an der Studie ist mit keinen Risiken verbunden.

Entstehen zusätzliche Kosten?

Durch die Teilnahme an der Studie entstehen Ihnen keine Kosten.

Was wird von mir erwartet?

Für die Teilnahme benötigen Sie nichts Weiteres. Alle notwendigen Materialien werden zur Verfügung gestellt. Die einzige Erwartung an Sie ist, dass Sie ihre Perspektive zu den verhandelten Themen kundtun.

Ist es möglich, im Verlauf aus der Studie auszuscheiden?

Ihre Teilnahme an dieser Studie ist freiwillig. Sie können jederzeit die Teilnahme beenden. Sie müssen dies nicht begründen. Es entstehen für Sie dadurch auch keine Nachteile.

Information zum Datenschutz

In dieser Studie ist der Lehrstuhl für Soziale Determinanten der Gesundheit für die Datenverarbeitung verantwortlich. Rechtsgrundlage für die Verarbeitung ist die persönliche

For peer review only - http://bmjopen.bmj.com/site/about/guidelines.xhtml

Einwilligung (Art. 6 Abs. 1a, Art. 9 Abs. 2a DSGVO). Die Daten werden zu jeder Zeit vertraulich behandelt.

Die Daten werden ausschließlich zum Zweck dieser oben beschriebenen Studie erhoben und nur in diesem Rahmen verwendet.

Die erfassten Daten umfassen auch personenbezogene Informationen, wie Ihre Stimme in der Tonbandaufnahme. Im nächsten Schritt wird die Tonbandaufnahme wortwörtlich niedergeschrieben, und danach wird die Aufnahme gelöscht. Alle Daten, durch die Sie unmittelbar identifiziert werden könnten, z.B. Ihr Name oder Ihr Geburtsdatum, werden durch einen Identifizierungscode ersetzt (pseudonymisiert). Damit ist es Unbefugten fast unmöglich, Sie zu identifizieren.

Die Daten werden an der Technische Universität München, Fakultät Sport- und Gesundheitswissenschaften, Lehrstuhl für Soziale Determinanten der Gesundheit gespeichert.

Wir bewahren die personenbezogenen Daten nur solange auf, wie dies für den oben genannten Zweck erforderlich ist. Die Daten werden spätestens nach Ablauf von 10 Jahre nach Abbruch oder Beendigung der Studie gelöscht.

Für die Studie führen wir eine Fokusgruppe mit Ihnen durch. Die Fokusgruppe vor Ort in Klinken wird mit Aufnahmegeräten aufgezeichnet. Die Fokusgruppen, welche während der Trainingsphase online via Videokonferenz durchgeführt werden, werden mittels eines zusätzlich eingeloggten Computers, auf welchem ein Aufnahmeprogramm läuft, aufgezeichnet. Die Tonaufnahme der Fokusgruppe wird zunächst an der Fakultät für Sport- und Gesundheitswissenschaften gespeichert. Das Gespräch wird innerhalb von sechs Monaten wortwörtlich aufgeschrieben (transkribiert). Die Tonaufnahmen werden dann gelöscht, sodass nur noch diese Abschrift des Gesprächs existiert. Alle Informationen, die dritten Personen Rückschluss auf Sie ermöglichen würden, werden bei der Abschrift so verändert, dass ein Rückschluss nicht mehr möglich ist. Der pseudonymisierte, aufgeschriebene Text wird für 10 Jahre in der Fakultät für Sport- und Gesundheitswissenschaften gespeichert und dann gelöscht. Wir übermitteln die personenbezogenen Daten weder an andere Einrichtungen in Deutschland, der EU, noch an ein Drittland außerhalb der EU oder an eine internationale Organisation.

Die Einwilligung zur Verarbeitung Ihrer Daten ist freiwillig. Sie können jederzeit die Einwilligung ohne Angabe von Gründen und ohne Nachteile für Sie widerrufen. Danach werden keine Daten mehr erhoben.

Sie können im Fall des Widerrufs die Löschung der erhobenen Daten verlangen. Die Daten können auch in anonymisierter Form weiterverwendet werden, wenn Sie dem zum Zeitpunkt Ihres Widerrufs zustimmen.

Sie haben das Recht, Auskunft über die Daten zu erhalten, auch in Form einer unentgeltlichen Kopie. Darüber hinaus können Sie die Berichtigung, Sperrung, Einschränkung der Verarbeitung oder Löschung sowie gegebenenfalls eine Übertragung der Daten verlangen. Die Rechtmäßigkeit der aufgrund der Einwilligung bis zum Widerruf erfolgten Verarbeitung wird hiervon nicht berührt.

Wenden Sie sich in diesen Fällen an:

For peer review only - http://bmjopen.bmj.com/site/about/guidelines.xhtml

Prof. Dr. Matthias Richter
Lehrstuhlinhaber - Soziale Determinanten der Gesundheit
Georg-Brauchle-Ring 60/62
80992 München
Tel.: +49 89 289 24190
richter.matthias@tum.de

Bei Rückfragen zur Datenverarbeitung und zur Einhaltung des Datenschutzes wenden Sie sich bitte an den Datenschutzbeauftragten:

Behördlicher Datenschutzbeauftragter der Technischen Universität München
Postanschrift: Arcisstr. 21, 80333 München
Telefon: 089/289-17052
E-Mail: beauftragter@datenschutz.tum.de

Sie haben ebenfalls das Recht, sich bei jeder Datenschutzaufsichtsbehörde zu beschweren. Eine Liste der Aufsichtsbehörden in Deutschland finden Sie unter: https://www.bfdi.bund.de/DE/Infothek/Anschriften_Links/anschriften_links-node.html

Die für Sie zuständige Aufsichtsbehörde erreichen Sie unter:

Bayerischer Landesbeauftragter für den Datenschutz
Postanschrift: Postfach 22 12 19, 80502 München
Hausanschrift: Wagmüllerstraße 18, 80538 München
E-Mail: poststelle@datenschutz-bayern.de

For peer review only

Probandeninformation und Einwilligungserklärung zum Forschungsvorhaben: Projekt zur Entwicklung, Erprobung und Evaluierung einer psychosozialen Präventionsleistung [PE³PP] - Fokussgruppeninterviews Einwilligungserklärung

Einwilligung zur Teilnahme

Ich wurde von ____________________________ über die Studie informiert. Ich habe die schriftliche Information und Einwilligungserklärung zu der oben genannten Studie erhalten und gelesen. Ich wurde ausführlich schriftlich und mündlich über den Zweck und den Verlauf der Studie, die Chancen und Risiken der Teilnahme und meine Rechte und Pflichten aufgeklärt. Ich hatte Gelegenheit Fragen zu stellen. Diese wurden zufriedenstellend und vollständig beantwortet. Zusätzlich zur schriftlichen Information wurden folgende Punkte besprochen:

____

____

Meine Einwilligung in die Teilnahme an der Studie ist freiwillig. Ich habe das Recht, meine Einwilligung jederzeit ohne Angabe von Gründen zu widerrufen, ohne dass mir dadurch Nachteile entstehen.

Ich willige hiermit in die Teilnahme an der oben genannten Studie ein.

____
Name der teilnehmenden Person in Druckbuchstaben

____
Ort, Datum Unterschrift der teilnehmenden Person

____
Name der aufklärenden Person in Druckbuchstaben

____
Ort, Datum Unterschrift der aufklärenden Person

Einwilligung zur Datenverarbeitung

Die Verarbeitung und Nutzung der persönlichen Daten für die oben genannte Studie erfolgt ausschließlich wie in der Information zur Studie beschrieben.

Ich willige hiermit in die beschriebene Verarbeitung meiner personenbezogenen Daten, insbesondere auch Gesundheitsdaten, ein.

____
Ort, Datum Unterschrift der teilnehmenden Person

____
Ort, Datum Unterschrift der aufklärenden Person

For peer review only

For peer review only - http://bmjopen.bmj.com/site/about/guidelines.xhtml

Reporting checklist for protocol of a clinical trial.

Based on the SPIRIT guidelines.

Instructions to authors

Complete this checklist by entering the page numbers from your manuscript where readers will find each of the items listed below.

Your article may not currently address all the items on the checklist. Please modify your text to include the missing information. If you are certain that an item does not apply, please write "n/a" and provide a short explanation.

Upload your completed checklist as an extra file when you submit to a journal.

In your methods section, say that you used the SPIRITreporting guidelines, and cite them as:

Chan A-W, Tetzlaff JM, Gøtzsche PC, Altman DG, Mann H, Berlin J, Dickersin K, Hróbjartsson A, Schulz KF, Parulekar WR, Krleža-Jerić K, Laupacis A, Moher D. SPIRIT 2013 Explanation and Elaboration: Guidance for protocols of clinical trials. BMJ. 2013;346:e7586

For peer review only

		Reporting Item	Page Number
**Administrative information**			
Title	#1	Descriptive title identifying the study design, population, interventions, and, if applicable, trial acronym	1

Trial registration	#2a	Trial identifier and registry name. If not yet registered, name of intended registry	2
Trial registration: data set	#2b	All items from the World Health Organization Trial Registration Data Set	n/a
Protocol version	#3	Date and version identifier	final
Funding	#4	Sources and types of financial, material, and other support	6; 16
Roles and responsibilities: contributorship	#5a	Names, affiliations, and roles of protocol contributors	16
Roles and responsibilities: sponsor contact information	#5b	Name and contact information for the trial sponsor	16
Roles and responsibilities: sponsor and funder	#5c	Role of study sponsor and funders, if any, in study design; collection, management, analysis, and interpretation of data; writing of the report; and the decision to submit the report for publication, including whether they will have ultimate authority over any of these activities	6-6
Roles and responsibilities: committees	#5d	Composition, roles, and responsibilities of the coordinating centre, steering committee, endpoint adjudication committee, data management team, and	n/a

For peer review only

		other individuals or groups overseeing the trial, if applicable (see Item 21a for data monitoring committee)	
**Introduction**			
Background and rationale	#6a	Description of research question and justification for undertaking the trial, including summary of relevant studies (published and unpublished) examining benefits and harms for each intervention	4-5; 9-11
Background and rationale: choice of comparators	#6b	Explanation for choice of comparators	n/a
Objectives	#7	Specific objectives or hypotheses	6; 8-11
Trial design	#8	Description of trial design including type of trial (eg, parallel group, crossover, factorial, single group), allocation ratio, and framework (eg, superiority, equivalence, non-inferiority, exploratory)	8
**Methods: Participants, interventions, and outcomes**			
Study setting	#9	Description of study settings (eg, community clinic, academic hospital) and list of countries where data will be collected. Reference to where list of study sites can be obtained	6-7

For peer review only

Eligibility criteria	#10	Inclusion and exclusion criteria for participants. If applicable, eligibility criteria for study centres and individuals who will perform the interventions (eg, surgeons, psychotherapists)	11-12
Interventions: description	#11a	Interventions for each group with sufficient detail to allow replication, including how and when they will be administered	6-8
Interventions: modifications	#11b	Criteria for discontinuing or modifying allocated interventions for a given trial participant (eg, drug dose change in response to harms, participant request, or improving / worsening disease)	n/a
Interventions: adherance	#11c	Strategies to improve adherence to intervention protocols, and any procedures for monitoring adherence (eg, drug tablet return; laboratory tests)	n/a
Interventions: concomitant care	#11d	Relevant concomitant care and interventions that are permitted or prohibited during the trial	n/a
Outcomes	#12	Primary, secondary, and other outcomes, including the specific measurement variable (eg, systolic blood pressure), analysis metric (eg, change from baseline, final value, time to event), method of aggregation (eg, median, proportion), and time point for each outcome. Explanation of the clinical relevance of chosen efficacy and harm outcomes is strongly recommended	13-14

For peer review only

Participant timeline	#13	Time schedule of enrolment, interventions (including any run-ins and washouts), assessments, and visits for participants. A schematic diagram is highly recommended (see Figure)	8-10
Sample size	#14	Estimated number of participants needed to achieve study objectives and how it was determined, including clinical and statistical assumptions supporting any sample size calculations	11-12
Recruitment	#15	Strategies for achieving adequate participant enrolment to reach target sample size	11-12
**Methods: Assignment of interventions (for controlled trials)**			
Allocation: sequence generation	#16a	Method of generating the allocation sequence (eg, computer-generated random numbers), and list of any factors for stratification. To reduce predictability of a random sequence, details of any planned restriction (eg, blocking) should be provided in a separate document that is unavailable to those who enrol participants or assign interventions	n/a
Allocation concealment mechanism	#16b	Mechanism of implementing the allocation sequence (eg, central telephone; sequentially numbered, opaque,	n/a

For peer review only

		sealed envelopes), describing any steps to conceal the sequence until interventions are assigned	
Allocation: implementation	#16c	Who will generate the allocation sequence, who will enrol participants, and who will assign participants to interventions	n/a
Blinding (masking)	#17a	Who will be blinded after assignment to interventions (eg, trial participants, care providers, outcome assessors, data analysts), and how	n/a
Blinding (masking): emergency unblinding	#17b	If blinded, circumstances under which unblinding is permissible, and procedure for revealing a participant's allocated intervention during the trial	n/a
**Methods: Data collection, management, and analysis**			
Data collection plan	#18a	Plans for assessment and collection of outcome, baseline, and other trial data, including any related processes to promote data quality (eg, duplicate measurements, training of assessors) and a description of study instruments (eg, questionnaires, laboratory tests) along with their reliability and validity, if known. Reference to where data collection forms can be found, if not in the protocol	12-14

For peer review only

Data collection plan: retention	#18b	Plans to promote participant retention and complete follow-up, including list of any outcome data to be collected for participants who discontinue or deviate from intervention protocols	n/a
Data management	#19	Plans for data entry, coding, security, and storage, including any related processes to promote data quality (eg, double data entry; range checks for data values). Reference to where details of data management procedures can be found, if not in the protocol	14
Statistics: outcomes	#20a	Statistical methods for analysing primary and secondary outcomes. Reference to where other details of the statistical analysis plan can be found, if not in the protocol	14-15
Statistics: additional analyses	#20b	Methods for any additional analyses (eg, subgroup and adjusted analyses)	n/a
Statistics: analysis population and missing data	#20c	Definition of analysis population relating to protocol non-adherence (eg, as randomised analysis), and any statistical methods to handle missing data (eg, multiple imputation)	n/a

Methods: Monitoring

Data monitoring: formal committee	#21a	Composition of data monitoring committee (DMC); summary of its role and reporting structure; statement of whether it is independent from the sponsor and competing interests; and reference to where further	14

For peer review only

		details about its charter can be found, if not in the protocol. Alternatively, an explanation of why a DMC is not needed	
Data monitoring: interim analysis	#21b	Description of any interim analyses and stopping guidelines, including who will have access to these interim results and make the final decision to terminate the trial	n/a
Harms	#22	Plans for collecting, assessing, reporting, and managing solicited and spontaneously reported adverse events and other unintended effects of trial interventions or trial conduct	n/a
Auditing	#23	Frequency and procedures for auditing trial conduct, if any, and whether the process will be independent from investigators and the sponsor	n/a
**Ethics and dissemination**			
Research ethics approval	#24	Plans for seeking research ethics committee / institutional review board (REC / IRB) approval	15
Protocol amendments	#25	Plans for communicating important protocol modifications (eg, changes to eligibility criteria, outcomes, analyses) to relevant parties (eg, investigators, REC / IRBs, trial participants, trial registries, journals, regulators)	n/a

For peer review only

Consent or assent	#26a	Who will obtain informed consent or assent from potential trial participants or authorised surrogates, and how (see Item 32)	15
Consent or assent: ancillary studies	#26b	Additional consent provisions for collection and use of participant data and biological specimens in ancillary studies, if applicable	n/a
Confidentiality	#27	How personal information about potential and enrolled participants will be collected, shared, and maintained in order to protect confidentiality before, during, and after the trial	15
Declaration of interests	#28	Financial and other competing interests for principal investigators for the overall trial and each study site	16
Data access	#29	Statement of who will have access to the final trial dataset, and disclosure of contractual agreements that limit such access for investigators	15
Ancillary and post trial care	#30	Provisions, if any, for ancillary and post-trial care, and for compensation to those who suffer harm from trial participation	n/a
Dissemination policy: trial results	#31a	Plans for investigators and sponsor to communicate trial results to participants, healthcare professionals, the public, and other relevant groups (eg, via publication, reporting in results databases, or other data sharing arrangements), including any publication restrictions	15

For peer review only

Dissemination policy: authorship	#31b	Authorship eligibility guidelines and any intended use of professional writers	15
Dissemination policy: reproducible research	#31c	Plans, if any, for granting public access to the full protocol, participant-level dataset, and statistical code	n/a

Appendices

Informed consent materials	#32	Model consent form and other related documentation given to participants and authorised surrogates	15
Biological specimens	#33	Plans for collection, laboratory evaluation, and storage of biological specimens for genetic or molecular analysis in the current trial and for future use in ancillary studies, if applicable	n/a

The SPIRIT Explanation and Elaboration paper is distributed under the terms of the Creative Commons Attribution License CC-BY-NC. This checklist was completed on 16. October 2023 using https://www.goodreports.org/, a tool made by the EQUATOR Network in collaboration with Penelope.ai

For peer review only

BMJ Open

Development, piloting, and evaluation of an app-supported psychosocial prevention intervention to strengthen participation in working life: A study protocol of a mixed-methods approach

Journal:	*BMJ Open*
Manuscript ID	bmjopen-2023-081390.R2
Article Type:	Protocol
Date Submitted by the Author:	31-Jan-2024
Complete List of Authors:	Stephan, Johannes; Technical University of Munich, TUM School of Medicine and Health, Chair of Social Determinants of Health<br>Gehrmann, Jan; Technical University of Munich, TUM School of Medicine and Health, Chair of Social Determinants of Health; Technical University of Munich, Institute of General Practice and Health Services Research<br>Stullich, Ananda; Technical University of Munich, TUM School of Medicine and Health, Chair of Social Determinants of Health<br>Hoffmann, Laura; Technical University of Munich, TUM School of Medicine and Health, Chair of Social Determinants of Health<br>Richter, Matthias; Technical University of Munich, TUM School of Medicine and Health, Chair of Social Determinants of Health<br>for the, PE^{3}PP study group; for the PE^{3}PP study group
**Primary Subject Heading**:	Health services research
Secondary Subject Heading:	Mental health, Public health
Keywords:	MENTAL HEALTH, PREVENTIVE MEDICINE, Burnout, QUALITATIVE RESEARCH, Quality of Life, Stress, Psychological

SCHOLARONE™
Manuscripts

Title

Development, piloting, and evaluation of an app-supported psychosocial prevention intervention to strengthen participation in working life: A study protocol of a mixed-methods approach

Authors:

Johannes Stephan*[1], Jan Gehrmann*[1,2], Ananda Stullich[1], Laura Hoffmann[1], Matthias Richter[1] for the PE³PP study group

* Both authors contributed equally to the manuscript and share the first authorship.

[1] Technical University of Munich, TUM School of Medicine and Health, Department Health and Sport Sciences, Chair of Social Determinants of Health

[2] Technical University of Munich, TUM School of Medicine and Health, Institute of General Practice and Health Services Research

Correspondence:

Johannes Stephan, Technical University of Munich, TUM School of Medicine and Health, Department Health and Sport Sciences, Chair of Social Determinants of Health, Georg-Brauchle-Ring 60/62, D-80922 Munich, Germany, Johannes.Stephan2@tum.de

Abstract

Introduction

Rates of incapacity to work due to mental disorders have increased in many European countries. The consequences of persistent stress can impact individuals' physical and psychological well-being and gradually develop into chronic stress. Mental disorders or symptoms of burnout syndrome can have severe consequences. Mental disorders leading to work incapacity significantly burden the health system. Prevention interventions can protect against burnout, depression, anxiety, and other mental health disorders. Digital health is a promising approach to increase the utilization of effective prevention interventions. This mixed-methods study evaluates a newly developed app-supported psychosocial prevention intervention called 'RV Fit Mental Health' to strengthen participation in working life.

Methods and analysis

The study uses a three-stage parallel mixed-methods design. This study accompanies the development (stage 1), piloting (stage 2), and evaluation (stage 3) of the new intervention. Within the stages, there is a quantitative as well as a qualitative research strand. Employed

For peer review only

persons with an incipient mental disorder will be included. Additionally, experts within the project or connected areas will be included. Quantitative data will be analyzed using multifactorial variance analyses in a pre-post design. Qualitative data will be analyzed using qualitative content analysis. The study is a comprehensive research approach to investigate the development, piloting, and evaluation of an app-supported psychosocial app-based prevention intervention. The rigor of the study will be achieved through data triangulation.

Ethics and dissemination

All participants will receive detailed study information and give written informed consent before data collection. Ethical approval was obtained from the Technical University of Munich Ethics Committee. All data collection will follow all legislative rules regarding data protection, also following the Declaration of Helsinki. The study results will be disseminated in peer-reviewed journals and presented at international conferences.

Trial registration number DRKS-ID: DRKS00030818 & DRKS00033080

Wordcount: 5.008 words

Keywords

mental health care, digital health, mixed methods, study protocol, prevention

Strengths and limitations of the study

- A mixed-methods research design is used for the entire study, which allows multiple perspectives to be focused on the same topic, leading to comprehensive results.
- The generalization of quantitative and qualitative results is challenging but offers various potentials for comparison and contrast regarding other innovative projects.
- The holistic approach integrating qualitative and quantitative data collection offers the potential to analyze the peculiarities of the digital dimension of the intervention.

For peer review only

1. Introduction

Over the past two decades, cases of incapacity to work due to mental disorders have increased in many European countries and represent a significant burden of disease [1, 2]. About one in five people have a current mental disorder (18% in Germany, 17.3% in the EU, 18.9% in the US, and 17.6% worldwide) [3–5]. In a study commissioned by the German health insurance company 'Techniker Krankenkasse' (TK), 47% of respondents reported that work, school, or study caused them to feel stressed. Participants stated that the main stressors were excessive workload, time pressure, rush, interruptions, and disruptions [6]. Hogan et al. described that long-term workplace stress is often caused by an imbalance between high work demands and the limited personal resources of workers [7]. The consequences of persistent work-related stress can impact individuals' physical and psychological well-being [8]. Excessive demands at work can gradually develop into chronic work stress, whereby symptoms of burnout syndrome can be consequences [8]. Long-term symptoms of burnout syndrome increase the risk of mental disorders such as sleep disorders, depressive symptoms, adjustment disorders, and physical health [8–10]. Employee performance, sick leave, absenteeism, accidents, and staff turnover are strongly influenced by mental health problems [9]. A relationship can also be shown between burnout and incapacity to work, as well as early entry into a disability pension [10].

A study by another German health insurance company, the 'Deutsche Angestellten Krankenkasse' (DAK), showed that the number of sick leave cases caused by mental health issues has almost tripled over the last 25 years [11]. The health report of the TK showed similar findings: The number of sick days due to mentally caused inability to work more than doubled from 2000 (129 sick days per 100 insurance years) to 2021 (283 sick days per 100 insurance years) in Germany. The main reasons for this increase were depressive episodes and reactions to severe stress and adjustment disorders [12]. Mental disorders cause the most days of incapacity to work at a stretch in Germany [11, 12]. Individuals who are permanently unable to work due to health reasons can apply for and receive a disability pension. The German Pension Insurance Association reports a significant increase, by double, in first-time granted disability pensions due to mental disorders in the last 25 years [13]. As a result, in the year 2021, mental disorders (41.7%) became the primary reason for disability pensions, followed by new formations such as cancer (15.3%), musculoskeletal disorders (11.5%), and cardiovascular diseases (9.1%) [13].

According to the OECD, in 2015, the costs of mental disorders in Germany accounted for approximately 4.8% of the Gross Domestic Product (GDP) [4]. Compared to the OECD-28 average, Germany's expenditures related to mental disorders are 0.7 percentage points above the average; Germany spends proportionally the second most on mental disorders in Europe [4].

For peer review only - http://bmjopen.bmj.com/site/about/guidelines.xhtml

The European Psychiatric Association states that various mental disorders can be prevented through evidence-based interventions by strengthening protective factors or reducing risk factors to promote mental health and disease prevention, e.g. through psychoeducation, skills training, stress management or other various therapeutic elements [14]. Consequently, psychosocial prevention interventions can protect against burnout, depression, anxiety, and other mental health disorders [8, 11, 15, 16]. Effective prevention interventions strengthen environmental and individual resources while reducing risk factors [17].

In Germany, the National Prevention Conference has therefore highlighted protecting and strengthening mental health in the workplace as a priority [18]. The German Pension Insurance supports this and acts according to the principle of 'prevention before rehabilitation' [19]. There is a general need to expand attractive evidence-based prevention interventions and improve access to and utilization of these services [20].

Determinants of the use of digital health applications

Digital Health is a promising approach to increase the utilization and quality of prevention interventions by using information and communication technologies to meet health-related needs [21]. Studies have shown that psychosocial aspects can be strengthened during inpatient treatment with the application of various therapies or elements such as individual or group training or psychoeducation [22–24]. Additionally, positive outcomes have been demonstrated for digitally supported mental health prevention interventions using a variety of therapeutic or psychoeducational elements [25–27]. Among the digitally supported prevention interventions that were not exclusively related to work, various outcomes were examined in employees, such as work stress [25], perceived stress [26], anxiety, and quality of life [27], which are either related to or exacerbated by the work environment. Digital health applications can be used flexibly in terms of time and location and can therefore be integrated into everyday life alongside work [25–27]. Individuals using digital health applications are exposed to various determinants influencing their effective and efficient use [28, 29]. One of the crucial determinants seems to be digital health literacy (DHL) [30]. DHL encompasses searching for and finding health information from digital sources, comprehending and assessing it, and applying acquired knowledge to address health-specific questions and solve problems [31]. It also encompasses skills and knowledge to interact productively with technology-enabled health tools [32, 33]. Other determinants that influence the utilization of digital health applications include cultural and social factors [33–35], motivation and interest [32, 33, 36, 37], and the accessibility and availability of technology [38–40]. Subsequently, there is evidence of a relationship between DHL and adherence to digital health interventions [41, 42].

For peer review only - http://bmjopen.bmj.com/site/about/guidelines.xhtml

When investigating the effectiveness of an app-supported psychosocial prevention intervention, it is crucial to collect data on the different determinants that affect utilizing a digital health application. By expanding concepts of DHL to include socio-cultural factors, motivation, interest, technology accessibility, and availability, a holistic assessment of DHL can be operationalized that incorporates the relevant determinants and allows predictions about the use of a health application.

Implementation and acceptance of digital health applications

The importance of analyzing the acceptance and framework conditions and contexts of digital health applications is emphasized regarding the use of digital health applications. Therefore, it is essential to account for organizational and contextual factors that impact the implementation of innovations in healthcare settings [43–45]. This is particularly important as it has been shown that evidence of effectiveness has not readily led to sustained implementation [46]. Especially in mental healthcare, the implementation of digital health applications poses various and unique challenges, highlighting the importance of evaluating the effectiveness and factors for successful implementation [45, 47]. Various studies highlight that it is necessary to analyze the interrelation between digital intervention and the healthcare context in which it is implemented [48, 49]. Thus, many digital mental health care interventions show better effects when paired with some therapeutic or doctoral support [50]. It is also highlighted that the perspective of health professionals as well as patients needs to be analyzed in the development to enable successful implementation [51–53]. Analyzing the implementation of a digital intervention needs to apply a comprehensive approach using quantitative and qualitative data. As the development, piloting, and evaluation is an iterative process, there is a need to analyze the different stages, anticipating the various perspectives involved, like users as well as healthcare professionals, but also focus on the contextual factors like organizational routines, established processes, and regulatory or policy aspects [48, 54].

Problem summary

In summary, the following problems were identified: First, stress experienced by individuals related to or exacerbated by the work environment can become chronic, negatively affecting their mental and physical well-being. Consequently, this places an increasing burden on the healthcare systems. Second, prevention interventions can protect against the chronification of stress; however, prevention interventions are rarely taken up in Germany. Third, digitally enabled prevention interventions can increase utilization. However, it cannot be assumed that different people can use digital health applications equally effectively. Fourth, there are measurement tools for assessing DHL, but existing measurement tools do not account for potential determinants that may impact the effective use of digital health applications. Fifth, regarding developing and implementing a new digital intervention, there is a need to analyze the acceptance and contextual factors.

For peer review only - http://bmjopen.bmj.com/site/about/guidelines.xhtml

The study aims to investigate the development, piloting, and evaluation of a psychosocial app-based prevention intervention using a comprehensive mixed methods approach. The intervention explicitly focused on mental health problems related to or exacerbated by the work environment, such as affective disorders, phobic and other anxiety disorders, adjustment disorders, somatoform disorders, and burnout. Its main aim is to improve the participation of those affected in their working life. The study protocol was developed using the SPIRIT checklist [55].

2. Methods and analysis

Study background

The project 'Development, piloting, and evaluation of an app-supported psychosocial prevention intervention' (German acronym PE³PP) is funded under the federal program 'Innovative Ways to Participate in Working Life – rehapro' of the Federal Ministry of Labor and Social Affairs Germany. The project runs from October 2021 until September 2026 and is led by the German Pension Insurance Central Germany.

This study protocol outlines the planned scientific accompaniment for developing, piloting, and evaluating a psychosocial app-supported prevention intervention called 'RV Fit Mental Health' by the Chair of Social Determinants of Health of the Technical University of Munich (TUM). The intervention aims to promote and strengthen individuals' participation in work life. It is a prospective multicentric study. The two central German rehabilitation clinics, Median Klinik Bad Gottleuba and SRH Medinet Burgenlandklinik, will lead in developing and conducting the intervention. Further PE³PP project partners are the health insurance companies 'Allgemeine Ortskrankenkasse Sachsen-Anhalt' (AOK SAN) in the State of Saxony-Anhalt and 'Allgemeine Ortskrankenkasse PLUS' (AOK PLUS) in the states of Saxony and Thuringia.

Study intervention

Innovation of the intervention

The psychosocial prevention intervention 'RV Fit Mental Health' aims to improve existing prevention interventions of the German Pension Insurance. The secondary prevention intervention is conceptually innovative, focusing on the psychological, psychosomatic, and psychosocial aspects of work-related participation disorders. It is formally innovative in terms of its temporal and methodological design. The duration of the intervention will be 14 weeks. It commences with a two-week inpatient initial phase in a rehabilitation clinic and a 12-week app-supported training phase. Participants receive intensive therapeutic support during the inpatient and training phases. The proactive recruitment of participants by German Pension Insurance Central Germany and the two health insurances, AOK SAN and AOK Plus, is also promising.

For peer review only - http://bmjopen.bmj.com/site/about/guidelines.xhtml

The framework of the intervention

The study is multicentric and will be conducted simultaneously at two clinics. 1020 participants are expected to undergo the intervention from September 2023 to September 2026. Participants complete the two-week inpatient phase in small groups of ten participants per clinic. Each participant will be assigned a therapeutic support person for both intervention phases. During the inpatient phase, the participant and therapeutic support person collaboratively establish prevention goals, resulting in a therapy plan for the inpatient and training phases.

Modules of the intervention

The prevention intervention covers various topics, including psychological stabilization, self-care, finding meaning and purpose, coping with multiple stressors and increasing digitalization, achieving work-life balance, and providing ‘first aid’ on topics such as burnout, depression, anxiety, sleep disorders, and pain. In addition to psychoeducational elements, the intervention includes training in social and emotional competencies, professional and personal stress management, interaction, and communication training, as well as the provision of mindfulness-based and body-oriented therapeutic elements. These substantive topics will be consolidated into modules, conveyed, and practiced during the inpatient and training phases.

Didactical realization of the intervention

During the inpatient phase, therapists conduct the modules in groups. Therapists conducting the modules include psychosomatic physicians, nutritionists, sports and physiotherapists, and psychologists, each possessing diverse expertise in interventions using a digital health application. In this process, all participants attend most of the modules. Additionally, there will be specific modules tailored to the individual needs of the participants. The content of the modules is conveyed and trained through presentations, lectures, exercises, and self-development phases.

In the training phase, the participants could flexibly arrange their training in terms of time and location. The training phase will be supported by an app, creating a digital connection between the rehabilitation clinic and participants. The app will contain the training plan designed by the therapeutic support person to achieve the prevention goals. The training plan will include various modules for practice during this phase. The therapeutic support person assigned to the training phase is a psychologist trained in the administrative aspects of using the app.

The app provides various methods and formats for delivering module content, such as exercises, instructions, training, and seminars in video and audio formats. In addition, interaction and communication features, including group chats and video call functions, enable participants to engage

For peer review only - http://bmjopen.bmj.com/site/about/guidelines.xhtml

with their therapeutic support person and other participants. Consequently, the app supports and accompanies the transfer of knowledge and behaviors initiated during the inpatient phase into the participants' daily lives.

During the training phase, each participant will have a coaching session with their therapeutic support person every four weeks. Participants also confer with their therapeutic support person the level of encouragement they needed to consistently train in the individual modules, as outlined in the intervention plan, between coaching sessions. This method aims to increase and maintain commitment to participation in therapy. Through the coaching sessions, the therapeutic support person and the participant will (re)evaluate the progress toward achieving the prevention goals. Moreover, group sessions will take place during the training phase, where participants from the same group in the inpatient phase and a therapeutic support person will work on relevant intervention content and exchange experiences within the group. In the 14th week of the prevention intervention, each participant will have an individual concluding session with their therapeutic support person, ending the intervention.

Design, aims, and research questions

The study follows a parallel mixed-methods design integrating quantitative and qualitative data [56–58]. This approach offers the opportunity to highlight multiple perspectives on the same topic and consequently betters comprehension. Mixed-methods research has several methodological challenges, especially regarding study rigor, data triangulation, and integration of the various data [57–59]. Data triangulation is a methodological approach that will contribute to the validity of the results by linking qualitative and quantitative data, offering various potentials for analysis through the dynamic and emergent process of data collection and analysis [60–63]. This is especially beneficial in evaluation and implementation research [64].

The study consists of three stages in which various research questions are answered (Figure 1). In stage one, we will collect qualitative data. Stages two and three have a qualitative and quantitative research strand with corresponding objectives and research questions.

Figure 1: Overview of the study stages

Stage 1: Development (July 2022 – August 2023)

Stage one (S1) aims to scientifically accompany the development of the 'RV Fit Mental Health' intervention. The central research question is: What needs to be done to provide an attractive, innovative psychosocial prevention intervention?

For peer review only - http://bmjopen.bmj.com/site/about/guidelines.xhtml

In this stage, the different dimensions of the development process and the requirements for the organizational implementation are of interest. This stage consists of a qualitative research strand. The results thus have an evaluative character in that they enable the optimization and adaptation of the needs of the experts involved in planning and conducting the intervention and provide indications for successful cooperation between the project partners.

Qualitative research questions

We will answer the following sub-research questions:

- What are the needs and requirements of project partners with regard to the development of psychosocial intervention?
- Does the app fulfill the required functionality for the app-supported training phase?

Stage 2: Piloting (September 2023 – December 2023)

In stage two (S2), we aim to answer the central research question: How should the psychosocial prevention intervention 'RV Fit Mental Health' be implemented?

We will pilot the intervention and assess its feasibility. Thus, three research objectives emerged for stage two. First, the needs and experiences of the participants will be analyzed to identify standards for addressing potential participants and to optimize the intervention. Second, the constructed instrument for evaluating the intervention will undergo a pretest and a feasibility assessment. As the instrument will include an assessment of DHL, we aim to identify additional skills, competencies, and prerequisites that need to be analyzed to predict effectiveness. This results in five qualitative and one quantitative sub-research questions for stage two.

Qualitative research questions

- How should an app-supported prevention intervention be designed and organized to be feasible and accepted by the participants and the experts?
- What needs, expectations, and experiences were the first participants able to perceive during the initial run-through of the prevention intervention?
- What are facilitating factors and barriers regarding the implementation of the intervention?
- Which possibilities for optimization occur to increase the fit and acceptance of the prevention intervention, especially regarding the fit between the inpatient and the training phase?

For peer review only - http://bmjopen.bmj.com/site/about/guidelines.xhtml

- What competencies, skills, and prerequisites should users of digital health apps possess besides digital health literacy?

Quantitative research question

- Is the constructed survey instrument to measure primary and secondary outcomes feasible for participants?

Stage 3: Evaluation (January 2023 – April 2026)

Stage three (S3) aims to analyze the implementation and execution of the intervention. The key research questions in the evaluation stage are: Is the implementation of the psychosocial prevention intervention achieved? Is the psychosocial prevention intervention successful?

We will examine organizational conditions, accessibility of the target group, and collaboration among the project partners to implement the prevention intervention. We will investigate this from the perspectives of professionals and intervention participants. Additionally, we will assess the effectiveness of the intervention. This results in six qualitative and four quantitative sub-research questions for stage three.

Qualitative research questions

- Is the implementation of the psychosocial prevention intervention successful? What facilitating factors and barriers occurred?
- Is the app-supported prevention intervention feasible and accepted by the participants and the experts?
- What are the conditions for the implementing prevention intervention in terms of organizational processes and resources? What are the requirements from the health care professional's point of view?
- How is the dovetailing between the inpatient and the digital phase? What are the facilitating factors and barriers in the independent use of the app, and what are the determinants for successful therapeutic support in the digital phase?
- How does the cooperation and collaboration of the different partners develop during the project?
- What are the potentials, challenges, and needs for adaptation?

For peer review only - http://bmjopen.bmj.com/site/about/guidelines.xhtml

Quantitative research questions

- What effect does the intervention have on the participants' ability to work, as measured by the Work Ability Index (WAI)?
- What effect does the intervention have on quality of life as measured by the World Health Organization of Life-BREF (WHOQOL-BREF)?
- What effect does the intervention have on perceived stress levels as measured by the Depression Anxiety Stress Scale-21 (DASS-21)?
- Is there a correlation between the level of digital health literacy, measured with the G-eHEALS and a holistic DHL instrument, and post-intervention levels of work ability, quality of life, and perceived stress?

Intervention participants and study sampling

Participants in the prevention intervention

There are two pathways through which individuals are recruited for the prevention intervention. Pathway one is carried out by the German Pension Insurance Central Germany, a regional institution responsible for two million employed people and over one million pensioners. Pathway two is carried out by the health insurance companies AOK SAN and AOK PLUS, regional institutions of the Health Insurance AOK in the east of Germany, insuring a combined 4.3 million citizens. The three insurance companies will contact eligible insureds and proactively suggest the RV Fit Mental Health prevention intervention.

Sample and recruitment for all study stages

Qualitative Sample - Participants of the Intervention (S2, S3)

Focus groups (FG) with intervention participants will be conducted during the piloting (S2) and evaluation (S3) stages. We will sample through a random sampling method, where data collection time points will be predetermined. We plan four FG in the S2 with 6-10 participants each. Ten FG, also with 6-10 participants each, are planned in S3. Overall, we plan up to 14 FG with intervention participants. Contact with the individual group participants for the qualitative data collection is initiated via the respective clinic staff. If there is interest in participation, the clinics forward the time and number of participants. Inclusion criteria are the participation in the prevention intervention and sufficient German language skills.

For peer review only

Qualitative Sample - Experts (S1, S2, S3)

Expert interviews with representatives of the German Pension Insurance Central Germany, the AOK SAN, and AOK Plus, and with the executing healthcare professionals from the intervention clinics, will be conducted during the development (S1) and evaluation (S3) stages. There will also be FG in the pilot (S2) stage with these experts. Experts involved in planning and conducting the intervention are included in all stages. Inclusion criteria are the involvement in the development and/or implementation of the intervention, either organizationally or therapeutically. Up to ten participants will participate in each point of the data collection. The TUM project team will proactively recruit the participants of the expert interviews and the FG.

Furthermore, additional expert interviews will be conducted in S2. Included are healthcare professionals (this may include physicians, psychologists, and psychotherapists) who have prescribed or recommended app-based (digital) health applications for disease management, health promotion, or prevention as part of a treatment pathway, individuals who are involved in health research regarding the topic of digital health and digital health literacy as well as individuals from health policy in the field of digital health. Excluded are individuals who have prescribed, recommended or are experienced in other digital health technologies, such as wearables (smartwatches, trackers, etc.), and people who do not work in healthcare. We will interview ten to twelve experts. The experts will be recruited via professional associations and proactively through the study team.

Quantitative Sample - Participants of the Intervention (S2, S3)

We aim to survey all participants from the intervention's piloting (S2) and evaluation (S3) stages. We will use a non-probability sample for recruitment. Participants in the prevention intervention will receive a flyer on recruitment for our study via the German Pension Insurance and the intervention clinics. On the flyer are instructions and online access for our quantitative data collection. In S2, we expect a total of 60 participants. In S3, we intend to include 960 participants for the pre-post-design effectiveness assessment. Inclusion criteria are the participation in the prevention intervention and sufficient German language skills. We expect dropouts, especially during the digital training phase.

Data collection

<u>Qualitative data collection</u>

The overall qualitative data collection is depicted graphically in Figure 2.

Figure 2: Overview of qualitative data collection

For peer review only - http://bmjopen.bmj.com/site/about/guidelines.xhtml

The FG with participants occurs both during the initial inpatient phase on-site at the clinics and online during the digital training phase. The FG in the inpatient phase occurs towards the end of the inpatient stay. The online FG takes place when at least four weeks of the digital training phase have been completed.

The interviews with the experts will be conducted online. Depending on the research questions in each stage, a semi-structured interview guide will be developed and pretested for each expert group. The interview guides will be developed following the principles of Helfferich [65]. Furthermore, we are following the methodical principles of expert interviews and recognize the context of the new intervention as well as the evaluative character of the expert interviews [66–68].

The FG with the experts will take place during a project workshop on-site. The FG is based on a purposive moderation guide starting with a stimulus recognizing the specified topics, the organizational contexts, and the environment of the intervention [69].

<u>Quantitative data collection</u>

Quantitative data will be collected from the study participants in S2 and S3 at four different points (t0-t3) through (online) surveys, depicted graphically in Figure 3.

- t0 (first survey) transpires when insured persons are confirmed that participation in the prevention intervention is possible.
- t1 (second survey) takes place at the beginning of the prevention intervention within the first two days of the initial inpatient phase.
- t2 (third survey) is conducted two weeks after t1, at the end of the initial inpatient phase.
- t3 (fourth survey) occurs 12 weeks after the inpatient phase at the end of the 12-week training phase.

Figure 3: Overview of quantitative data collection

The primary outcomes of interest are workability, assessed using the German Work Ability Index [70]; quality of life, using the German WHOQOL-BREF [71, 72]; stress levels, evaluated using the DASS-21 [73–75]; and eHealth literacy, which will be assessed using the G-eHEALS [76–78], and a self-developed instrument for holistic DHL. The secondary outcomes are anxiety and depressive symptoms (DASS-21) and self-efficacy using the SWOP K9 [79–82]. Further information on the validity and reliability of the assessment instruments is provided in 'Supplementary Material 1'. In addition, we are interested in health behavior as a secondary outcome, which we operationalize as alcohol consumption, smoking, physical activity, and dietary behavior. The training phase behavioral data follow the FITT criteria [83, 84]: frequency, intensity, time, and type. We will use FITT data to

For peer review only - http://bmjopen.bmj.com/site/about/guidelines.xhtml

examine participants' adherence to the eHealth application [85]. Additionally, sociodemographic data will be collected during survey t1, and behavioral data from the training phase will be gathered during survey t3.

Data analysis

Qualitative analysis

The semi-structured interviews and the FG will be conducted by the research team (JG, JS), audiotaped, verbatim transcribed, and analyzed following Content Analysis, using MAXQDA 2022 [86, 87]. Qualitative content analysis is a systematic, rule-bound approach orientated towards rules of text analysis laid down in advance and contributes to the intersubjectivity of the analysis procedure regarding the defined interests [87]. The analytical steps taken by the category system are, firstly, the summary and the inductive category formation, then secondly by the explication and the context analysis, finalized by the structuring and the deductive category formation. The category system will then be re-checked by applying it to the material, the research questions, and the case itself [88]. Constant exchange within the study team and the working group for Qualitative Research at the Chair for Social Determinants of Health will ensure the rigor and integrity of the analysis. The analysis will be framed by applying content-analytical quality criteria and reported using the consolidated criteria for reporting qualitative research (COREQ) [89].

Quantitative analysis

Quantitative data analysis will be conducted using the IBM *Statistical Package for Social Sciences* (SPSS) software. During S2, the dataset will undergo a preliminary examination for meaningfulness. This involves checking for the data's completeness, outliers, and plausibility. A descriptive characterization of the quantitative S2 sample will be performed based on sociodemographic data to gain initial insights into the sample.

For S3, we plan a pre-post design effectiveness evaluation. The baseline data will be compared with the data from t1-t3. For this, we will conduct multifactorial variance analyses to investigate whether there are differences in the groups of individual intervention time points regarding the primary and secondary outcomes, thus demonstrating the potential effect of the prevention intervention. Additionally, we will perform regression analyses to interpret the relationship between the collected variables, allowing for statements about the influence of individual variables on different outcomes. Similarly, we present a differentiated sample characterization for S3. In addition, we add a waitlist-controlled component. After collecting data from t0 to t4, we divide participants of S3 into an intervention and waitlist group. We conduct t-tests within each group to analyze effects between t0

For peer review only - http://bmjopen.bmj.com/site/about/guidelines.xhtml

and t1 for the waitlist group and between t1 and t3 for the intervention group. Calculating Cohen's d helps us quantify effect sizes from these tests. We then use a Z-test to compare Cohen's d values of both groups to determine if there are significant differences in effect sizes.

Patient and public involvement

None.

3. Ethics and dissemination

Ethical approval was obtained from the Ethics Committee at the TUM School of Medicine and Health (2022-523-S-SR; 2023-316-S-SB). Following the Declaration of Helsinki, we will conduct the study according to ethical research principles, including the primacy of participant welfare, informed consent (example of participants' declaration of consent as 'Supplementary Material 2'), scientific validity, review by an ethics committee, appropriate risk-benefit balance, protection of vulnerable groups, data security and confidentiality, transparent reporting, and voluntary participation. In the inpatient phase, therapists inform participants about the potential disadvantages of app use for the training phase and discuss strategies to mitigate these issues. During the coaching sessions in the training phase, the therapeutic support person systematically identifies any possible disadvantages of using the app. If any study participant decides not to participate or not continue the study, they may withdraw consent at any time without any consequences. All data management follows the requirements of the German version of the General Data Protection Regulation and is approved by the TUM internal department for data security. All data collected in the study will be pseudonymized or, if possible, anonymized. The pseudonymization through a code ensures data protection of all data. All data containing identificatory characteristics (e.g., record, pseudonym assignment list, etc.) is secured separately from study data. They are stored in locked rooms at the Chair of Social Determinants of Health. We will delete all data once the study has been completed, but at the latest, after the given legal restriction of 10 years. In case of withdrawal, all data will be deleted entirely or, in extraordinary cases, evaluated carefully anonymized. Deleting data from already analyzed or published data is not possible. We will publish all analyzed data without any possibility of identifying persons. All results of the quantitative analysis will be published anonymously, and the results of the qualitative analysis will be pseudonymized. The study results will be disseminated in international peer-reviewed journals and presented at international conferences.

4. Authors´ contributions

JG and JS wrote the initial manuscript draft, will collect the quantitative and qualitative data, and are responsible for data analyses. LH and MR were involved in the grant application. JS and JG wrote the study protocol for the Ethics Review Committee. LH, AS, and MR critically reviewed the article several times and counseled the entire process of this study protocol and research All authors have

For peer review only

read and approved the final version of the manuscript. The study protocol has been developed on behalf of the PE³PP study group.

5. Funding statement

This study is supported by the Federal Ministry of Labour and Social Affairs as part of the rehapro funding program and is led by the German Pension Insurance of Central Germany (662S0033X1).

6. Competing interests statement

The authors of this study declare no conflicts of interest.

7. Acknowledgment statement

The authors thank the PE³PP study group for their support. Members of the PE³PP study group: Olaf Ballaschke, Sylvia Berlin, Florian Finke, Frank Gabel, Anastasia Grossmann, Claudia Gruhner, Kathleen Kaminski, Andreas Seemann (in alphabetical order).

8. References

REFERENCES

1 Hildebrandt-Heene S, Dehl T, Zich K, et al. Gesundheitsreport 2023: Analyse der Arbeitsunfähigkeiten. Gesundheitsrisiko Personalmangel: Arbeitswelt unter Druck. Heidelberg: medhochzwei Verlag GmbH 2023.

2 Salomonsson S, Hedman-Lagerlöf E, Öst L-G. Sickness absence: a systematic review and meta-analysis of psychological treatments for individuals on sick leave due to common mental disorders. *Psychol Med* 2018;48(12):1954–65. doi:10.1017/S0033291718000065 [published Online First: 30 January 2018].

3 Müller G, Bombana M, Heinzel-Gutenbrenner M, et al. Socio-economic consequences of mental distress: quantifying the impact of self-reported mental distress on the days of incapacity to work and medical costs in a two-year period: a longitudinal study in Germany. *BMC Public Health* 2021;21(1):625. doi:10.1186/s12889-021-10637-8 [published Online First: 31 March 2021].

4 OECD. Health at a Glance: Europe 2018. Brussels: OECD 2018.

5 Steel Z, Marnane C, Iranpour C, et al. The global prevalence of common mental disorders: a systematic review and meta-analysis 1980-2013. *Int J Epidemiol* 2014;43(2):476–93. doi:10.1093/ije/dyu038 [published Online First: 19 March 2014].

6 Meyer B, Zill A, Dibla D. Entspann dich, Deutschland!: TK Stressstudie 2021. Hamburg: Technicker Krankenkasse 2021.

7 Hogan L-M, Björklund Carlstedt A, Wagman P. Occupational therapy and stress-related exhaustion - a scoping review. *Scand J Occup Ther* 2023:1–17. doi:10.1080/11038128.2023.2207802 [published Online First: 8 June 2023].

8 Edú-Valsania S, Laguía A, Moriano JA. Burnout: A Review of Theory and Measurement. *Int J Environ Res Public Health* 2022;19(3). doi:10.3390/ijerph19031780 [published Online First: 4 February 2022].

9 Wilken U, Breucker G. Mental health in the workplace: Situation analysis Germany. Geneva: International Labour Organization 2000.

10 Ahola K, Gould R, Virtanen M, et al. Occupational burnout as a predictor of disability pension: a population-based cohort study. *Occup Environ Med* 2009;66(5):284-90;

discussion 282-3. doi:10.1136/oem.2008.038935 [published Online First: 18 November 2008].
11 Marschall J, Hildebrandt S, Nolting H-D. DAK-Gesundheitsreport 2019 2019. https://www.dak.de/dak/download/dak-gesundheitsreport-2019-sucht-pdf-2073718.pdf.
12 Grobe T, Bessel S. Gesundheitsreport 2022 Arbeitsunfähigkeit. Göttingen: aQua – Institut für angewandte Qualitätsförderung und Forschung im Gesundheitswesen GmbH 2022.
13 German Pension Insurance Association. Erwerbsminderungsrenten im Zeitablauf 2022 2022. Available at: https://www.deutsche-rentenversicherung.de/DRV/DE/Experten/Zahlen-und-Fakten/Statistiken-und-Berichte/statistiken-und-berichte_node.html Accessed July 27, 2023.
14 Campion J, Bhui K, Bhugra D. European Psychiatric Association (EPA) guidance on prevention of mental disorders. *Eur Psychiatry* 2012;27(2):68–80. doi:10.1016/j.eurpsy.2011.10.004 [published Online First: 28 January 2012].
15 Lehofer M, Glehr R, Haring C, et al. Burnout & Depression. Ein Leitfaden zur Prävention, Früherkennung und Behandlung. Experten-Statement [Burnout and Depression. A Guideline for Prevention, Early Diagnosis, and Intervention. Experts Statement] 2011.
16 Singh V, Kumar A, Gupta S. Mental Health Prevention and Promotion-A Narrative Review. *Front Psychiatry* 2022;13:898009. doi:10.3389/fpsyt.2022.898009 [published Online First: 26 July 2022].
17 World Health Organization. Mental Health 2022. Available at: https://www.who.int/news-room/fact-sheets/detail/mental-health-strengthening-our-response Accessed July 25, 2023.
18 National Prevention Conference. Spezielle Ziele für die Zielgruppe Erwerbstätige 2022. Available at: https://www.npk-info.de/praeventionsstrategie/bundesrahmenempfehlungen/spezielle-ziele-fuer-die-zielgruppe-erwerbstaetige Accessed July 26, 2023.
19 German Pension Insurance Association. Reha-Bericht 2021. Berlin: H. Heenemann GmbH & Co. KG 2021.
20 Robert Koch-Institut. Wie steht es um Prävention und Gesundheitsförderung?: RKI-Bib1 (Robert Koch-Institut) 2015.
21 World Health Organization. Global strategy on digital health 2020-2025. Geneve: World Health Organization 2021.
22 Booth S, Ryan R, Clow A, et al. Enhancing well-being and building resilience in people living with cancer part 2: a central role for nurses. *Cancer Nursing Practice* 2019;18(1):42–50.
23 Goldbach N, Reif A, Preuss H, et al. A randomized trial of group therapy for resource activation for patients with affective disorder. *Journal of Psychotherapy Integration* 2023;33(1):1–19.
24 Breuer K, John SM, Finkeldey F, et al. Tertiary individual prevention improves mental health in patients with severe occupational hand eczema. *J Eur Acad Dermatol Venereol* 2015;29(9):1724–31. doi:10.1111/jdv.12975 [published Online First: 4 February 2015].
25 Bostock S, Crosswell AD, Prather AA, et al. Mindfulness on-the-go: Effects of a mindfulness meditation app on work stress and well-being. *J Occup Health Psychol* 2019;24(1):127–38.
26 Champion L, Economides M, Chandler C. The efficacy of a brief app-based mindfulness intervention on psychosocial outcomes in healthy adults: A pilot randomised controlled trial. *PLoS One* 2018;13(12):e0209482.

For peer review only

27 Zhou T, Li X, Pei Y, et al. Internet-based cognitive behavioural therapy for subthreshold depression: a systematic review and meta-analysis. *BMC Psychiatry* 2016;16(1):356.
28 Li J, Theng Y-L, Foo S. Predictors of online health information seeking behavior: Changes between 2002 and 2012. *Health Informatics J* 2016;22(4):804–14. doi:10.1177/1460458215595851 [published Online First: 10 August 2015].
29 Perich T, Andriessen K. Predictors of digital technology-based mental health programs in young adults for mental health support. *Health Promot Int* 2023;38(3).
30 van Kessel R, Wong BLH, Clemens T, et al. Digital health literacy as a super determinant of health: More than simply the sum of its parts. *Internet Interv* 2022;27:100500. doi:10.1016/j.invent.2022.100500 [published Online First: 7 February 2022].
31 Norman CD, Skinner HA. eHealth Literacy: Essential Skills for Consumer Health in a Networked World. *J Med Internet Res* 2006;8(2):e9. doi:10.2196/jmir.8.2.e9 [published Online First: 16 June 2006].
32 Dias N, Yamamoto R, Faulkner KG. Preferences and motivational factors for the use of digital technology in real world oncology studies. *JCO* 2020;38(15_suppl):e14119-e14119.
33 Sifat MS, Saperstein SL, Tasnim N, et al. Motivations Toward Using Digital Health and Exploring the Possibility of Using Digital Health for Mental Health in Bangladesh University Students: Cross-sectional Questionnaire Study. *JMIR Form Res* 2022;6(3):e34901. doi:10.2196/34901 [published Online First: 4 March 2022].
34 Ehrari H, Tordrup L, Müller S. The Digital Divide in Healthcare: A Socio-Cultural Perspective of Digital Literacy. Proceedings of the 55th Hawaii International Conference on System Sciences 2022 2022.
35 Hsieh JJP-A, Rai A, Keil M. Addressing Digital Inequality for the Socioeconomically Disadvantaged Through Government Initiatives: Forms of Capital That Affect ICT Utilization. *Information Systems Research* 2011;22(2):233–53.
36 Kaihlanen A-M, Virtanen L, Buchert U, et al. Towards digital health equity - a qualitative study of the challenges experienced by vulnerable groups in using digital health services in the COVID-19 era. *BMC Health Serv Res* 2022;22(1):188. doi:10.1186/s12913-022-07584-4 [published Online First: 12 February 2022].
37 Park DY, Kim H. Determinants of Intentions to Use Digital Mental Healthcare Content among University Students, Faculty, and Staff: Motivation, Perceived Usefulness, Perceived Ease of Use, and Parasocial Interaction with AI Chatbot. *Sustainability* 2023;15(1):872.
38 World Health Organization. Equity within digital health technology within the WHO European Region: a scoping review. Copenhagen: WHO Regional Office for Europe 2022.
39 Sieck CJ, Sheon A, Ancker JS, et al. Digital inclusion as a social determinant of health. *NPJ Digit Med* 2021;4(1):52. doi:10.1038/s41746-021-00413-8 [published Online First: 17 March 2021].
40 Richardson S, Lawrence K, Schoenthaler AM, et al. A framework for digital health equity. *NPJ Digit Med* 2022;5(1):119. doi:10.1038/s41746-022-00663-0 [published Online First: 18 August 2022].
41 Lu X, Zhang R. Association Between eHealth Literacy in Online Health Communities and Patient Adherence: Cross-sectional Questionnaire Study. *J Med Internet Res* 2021;23(9):e14908. doi:10.2196/14908 [published Online First: 13 September 2021].
42 Do BN, Tran TV, Phan DT, et al. Health Literacy, eHealth Literacy, Adherence to Infection Prevention and Control Procedures, Lifestyle Changes, and Suspected

For peer review only - http://bmjopen.bmj.com/site/about/guidelines.xhtml

COVID-19 Symptoms Among Health Care Workers During Lockdown: Online Survey. *J Med Internet Res* 2020;22(11):e22894. doi:10.2196/22894 [published Online First: 12 November 2020].
43 Powell BJ, McMillen JC, Proctor EK, et al. A compilation of strategies for implementing clinical innovations in health and mental health. *Med Care Res Rev* 2012;69(2):123–57. doi:10.1177/1077558711430690 [published Online First: 26 December 2011].
44 Powell BJ, Waltz TJ, Chinman MJ, et al. A refined compilation of implementation strategies: results from the Expert Recommendations for Implementing Change (ERIC) project. *Implement Sci* 2015;10:21. doi:10.1186/s13012-015-0209-1 [published Online First: 12 February 2015].
45 Graham AK, Lattie EG, Powell BJ, et al. Implementation strategies for digital mental health interventions in health care settings. *Am Psychol* 2020;75(8):1080–92.
46 Mohr DC, Weingardt KR, Reddy M, et al. Three Problems With Current Digital Mental Health Research . and Three Things We Can Do About Them. *Psychiatr Serv* 2017;68(5):427–29. doi:10.1176/appi.ps.201600541 [published Online First: 17 April 2017].
47 Mohr DC, Lyon AR, Lattie EG, et al. Accelerating Digital Mental Health Research From Early Design and Creation to Successful Implementation and Sustainment. *J Med Internet Res* 2017;19(5):e153. doi:10.2196/jmir.7725 [published Online First: 10 May 2017].
48 Shaw J, Agarwal P, Desveaux L, et al. Beyond "implementation": digital health innovation and service design. *npj Digital Med* 2018;1(1):48. doi:10.1038/s41746-018-0059-8 [published Online First: 20 September 2018].
49 Marwaha JS, Landman AB, Brat GA, et al. Deploying digital health tools within large, complex health systems: key considerations for adoption and implementation. *npj Digit. Med.* 2022;5(1):13. doi:10.1038/s41746-022-00557-1 [published Online First: 27 January 2022].
50 Lattie EG, Stiles-Shields C, Graham AK. An overview of and recommendations for more accessible digital mental health services. *Nat Rev Psychol* 2022;1(2):87–100. https://www.nature.com/articles/s44159-021-00003-1.
51 Mendes-Santos C, Nunes F, Weiderpass E, et al. Understanding Mental Health Professionals' Perspectives and Practices Regarding the Implementation of Digital Mental Health: Qualitative Study. *JMIR Form Res* 2022;6(4):e32558. doi:10.2196/32558 [published Online First: 12 April 2022].
52 Lukka L, Karhulahti V-M, Palva JM. Factors Affecting Digital Tool Use in Client Interaction According to Mental Health Professionals: Interview Study. *JMIR Hum Factors* 2023;10:e44681. doi:10.2196/44681 [published Online First: 10 July 2023].
53 Birnbaum F, Lewis D, Rosen RK, et al. Patient engagement and the design of digital health. *Acad Emerg Med* 2015;22(6):754–56. doi:10.1111/acem.12692 [published Online First: 21 May 2015].
54 Desveaux L, Soobiah C, Bhatia RS, et al. Identifying and Overcoming Policy-Level Barriers to the Implementation of Digital Health Innovation: Qualitative Study. *J Med Internet Res* 2019;21(12):e14994. doi:10.2196/14994 [published Online First: 20 December 2019].
55 Chan AW, Tetzlaff JM, Altman DG, et al. SPIRIT 2013 statement: defining standard protocol items for clinical trials. *Ann Intern Med* 2013;158(3):200–07.
56 Tariq S, Woodman J. Using mixed methods in health research. *JRSM Short Rep* 2013;4(6):2042533313479197. doi:10.1177/2042533313479197 [published Online First: 7 May 2013].

For peer review only

For peer review only - http://bmjopen.bmj.com/site/about/guidelines.xhtml

57 Kuckartz U. Mixed Methods: Methodologie, Forschungsdesigns und Analyseverfahren: Springer Fachmedien Wiesbaden 2014.
58 Fetters MD, Curry LA, Creswell JW. Achieving integration in mixed methods designs-principles and practices. *Health Services Research* 2013;48(6 Pt 2):2134–56. doi:10.1111/1475-6773.12117 [published Online First: 23 October 2013].
59 Wisdom JP, Cavaleri MA, Onwuegbuzie AJ, et al. Methodological reporting in qualitative, quantitative, and mixed methods health services research articles. *Health Services Research* 2012;47(2):721–45. doi:10.1111/j.1475-6773.2011.01344.x [published Online First: 8 November 2011].
60 Casey D, Murphy K. Issues in using methodological triangulation in research. *Nurse Researcher* 2009;16(4):40–55.
61 Farmer T, Robinson K, Elliott SJ, et al. Developing and implementing a triangulation protocol for qualitative health research. *Qualitative Health Research* 2006;16(3):377–94.
62 Gibson CB. Elaboration, Generalization, Triangulation, and Interpretation. *Organizational Research Methods* 2017;20(2):193–223.
63 Jick TD. Mixing Qualitative and Quantitative Methods: Triangulation in Action. *Administrative Science Quarterly* 1979;24(4):602.
64 Green CA, Duan N, Gibbons RD, et al. Approaches to Mixed Methods Dissemination and Implementation Research: Methods, Strengths, Caveats, and Opportunities. *Adm Policy Ment Health* 2015;42(5):508–23.
65 Helfferich C. Die Qualität qualitativer Daten: Manual für die Durchführung qualitativer Interviews, 4th edn. Wiesbaden: VS Verlag 2011.
66 Meuser M, Nagel U. The Expert Interview and Changes in Knowledge Production. In: Interviewing Experts: Palgrave Macmillan, London 2009:17–42.
67 Döringer S. 'The problem-centred expert interview'. Combining qualitative interviewing approaches for investigating implicit expert knowledge. *International Journal of Social Research Methodology* 2021;24(3):265–78.
68 Wroblewski A, Leitner A. Between Scientific Standards and Claims to Efficiency: Expert Interviews in Programme Evaluation. In: Interviewing Experts: Palgrave Macmillan, London 2009:235–51.
69 Smithson J. Using and analysing focus groups: Limitations and possibilities. *International Journal of Social Research Methodology* 2000;3(2):103–19.
70 Freyer M. Eine Konstruktvalidierung des Work Ability Index anhand einer repräsentativen Stichprobe von Erwerbstätigen in Deutschland: Bundesanstalt für Arbeitsschutz und Arbeitsmedizin (BAuA) 2019.
71 Skevington SM, Lotfy M, O'Connell KA. The World Health Organization's WHOQOL-BREF quality of life assessment: psychometric properties and results of the international field trial. A report from the WHOQOL group. *Qual Life Res* 2004;13(2):299–310.
72 World Health Organization. WHOQOL. User Manual. Geneva: World Health Organization 2012.
73 Antony MM, Bieling PJ, Cox BJ, et al. Psychometric properties of the 42-item and 21-item versions of the Depression Anxiety Stress Scales in clinical groups and a community sample. *Psychological Assessment* 1998;10(2):176–81.
74 Nilges P, Essau C. Die Depressions-Angst-Stress-Skalen: Der DASS--ein Screeningverfahren nicht nur für Schmerzpatienten. *Schmerz* 2015;29(6):649–57.
75 Nilges P, Essau C. DASS. Depressions-Angst-Stress-Skalen - deutschsprachige Kurzfassung: ZPID (Leibniz Institute for Psychology) – Open Test Archive 2021.

For peer review only

76 Chung S-Y, Nahm E-S. Testing reliability and validity of the eHealth Literacy Scale (eHEALS) for older adults recruited online. *Comput Inform Nurs* 2015;33(4):150–56.
77 Norman CD, Skinner HA. eHEALS: The eHealth Literacy Scale. *J Med Internet Res* 2006;8(4):e27. doi:10.2196/jmir.8.4.e27 [published Online First: 14 November 2006].
78 Soellner R, Huber S, Reder M. The Concept of eHealth Literacy and Its Measurement. *Journal of Media Psychology* 2014;26(1):29–38.
79 Jerusalem M, Schwarzer R. "Selbstwirksamkeit". WIRK. In: Schwarzer R, ed. Skalen zur Befindlichkeit und Persönlichkeit. Berlin: Berlin Freie Universität 1986:15–28.
80 Scheier MF, Carver CS. Optimism, coping, and health: assessment and implications of generalized outcome expectancies. *Health Psychol* 1985;4(3):219–47.
81 Scholler G, Fliege H, Klapp BF. Fragebogen zu Selbstwirksamkeit, Optimismus und Pessimismus. Restrukturierung, Itemselektion und Validierung eines Instrumentes an Untersuchungen klinischer Stichproben. *Psychother Psychosom Med Psychol* 1999;49(8):275–83.
82 Scholler G, Fliege H, Klapp BF. SWOP-K9 - Fragebogen zu Selbstwirksamkeit-Optimismus-Pessimismus Kurzform: ZPID (Leibniz Institute for Psychology Information) – Testarchiv 1999.
83 Barisic A, Leatherdale ST, Kreiger N. Importance of frequency, intensity, time and type (FITT) in physical activity assessment for epidemiological research. *Can J Public Health* 2011;102(3):174–75.
84 Short CE, DeSmet A, Woods C, et al. Measuring Engagement in eHealth and mHealth Behavior Change Interventions: Viewpoint of Methodologies. *J Med Internet Res* 2018;20(11):e292. doi:10.2196/jmir.9397 [published Online First: 16 November 2018].
85 Sieverink F, Kelders SM, van Gemert-Pijnen JE. Clarifying the Concept of Adherence to eHealth Technology: Systematic Review on When Usage Becomes Adherence. *J Med Internet Res* 2017;19(12):e402. doi:10.2196/jmir.8578 [published Online First: 6 December 2017].
86 Mayring P. Qualitative Content Analysis. *Forum qualitative Sozialforschung* 2000;1(2).
87 Mayring P. Qualitative content analysis: theoretical foundation, basic procedures and software solution. Klagenfurt 2014.
88 Kohlbacher F. The Use of Qualitative Content Analysis in Case Study Research. *Forum qualitative Sozialforschung* 2006;7(1).
89 Tong A, Sainsbury P, Craig J. Consolidated criteria for reporting qualitative research (COREQ): a 32-item checklist for interviews and focus groups. *Int J Qual Health Care* 2007;19(6):349–57. doi:10.1093/intqhc/mzm042 [published Online First: 14 September 2007].

For peer review only

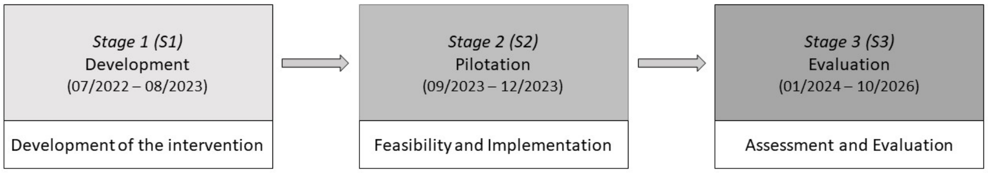

Figure1: Overview of the study stages

338x190mm (96 x 96 DPI)

Development (07/2022 – 08/2023)	Pilotation (09/2023 – 12/2023)	Evaluation (01/2024 – 10/2026)
**Expert interviews**<br>8 interviews	**Expert interviews**<br>10-12 interviews<br><br>**Focus groups with experts**<br>1-2 focus groups (each n=4-5)<br><br>**Focus groups with participants**<br>4 focus groups each with different cohorts – two focus groups (each n=6-10) during the inpatient phase & two focus groups during the training phase (each n=6-10)	**Expert interviews**<br>8-10 interviews<br><br>**Focus groups with participants**<br>10 focus groups (each n=6-10) in an intervall of six months with different cohorts of the intervention; evenly divided in inpatient phase as well as training phase

Overview qualitative data collection:
Expert interviews (n=26-30)
Expert focus groups (n=4-10)
Focus group participants (n=84-140)

Figure2: Overview of qualitative data collection

338x190mm (96 x 96 DPI)

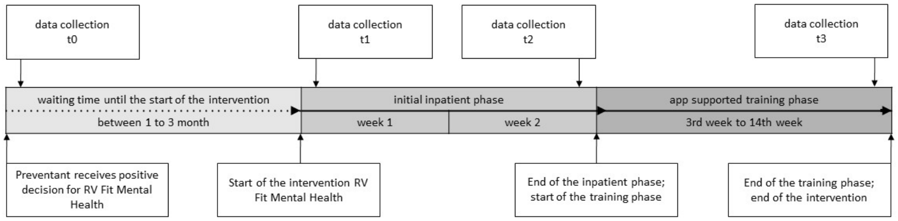

Figure 3: Overview of quantitative data collection

338x190mm (96 x 96 DPI)

Information on the validity and reliability of the assessment instruments

Instrument full name	Measurement	Number of items	Instrument abbreviation	Validity and reliability	Source
**Depression Anxiety Stress Scale-21**	depression, anxiety, stress	21	DASS-21	The participants in the total sample (n=307) can be divided into several groups based on their diagnosis and a group of people from the general population.<br>Panic disorder with or without agoraphobia: n=67, obsessive-compulsive disorder: n=54, social phobia: n=74, specific phobia: n=17, major depressive disorder: n=46, and non-clinical volunteers: n=49.<br>Internal consistency values for each subscale: depression α=0.94, anxiety α=0.87, and stress α=0.91.	[73]
			DASS-21 (German Version)	German Version of the DASS-21. Participants of the total sample size (n=714) was divided into two groups:<br>Pain patients: n=301, adults in the general population: n=413.<br>Internal consistency values for each subscale: depression α=0.88, anxiety α=0.76-0.80, stress α=0.86-0.87.	[74]
**eHealth Literacy Scale**	ability to find, evaluate, and apply health information from the internet	8	eHEALS	The total sample size comprised 664 participants aged 13–21 years.<br>The internal consistency of the overall scale is α=0.88.	[77]
			eHEALS	The total sample (n=866) consisted of people aged 50 years and over who were recruited for the Bone Power Study via two online portals.<br>The internal consistency of the overall scale is α=0.94.	[76]
			G-eHEALS (German Version)	This is a version of the eHEALS that has been translated into German. The sample (n=327) consisted of German students.<br>Reliability was tested using a 2-factor model, which resulted in α=0.88 for the information search dimension and α=0.82 for the information evaluation dimension.	[78]

1

Information on the validity and reliability of the assessment instruments

Instrument full name	Measurement	Number of items	Instrument abbreviation	Validity and reliability	Source
**Self-efficacy-optimism-pessimism questionnaire**	self-efficacy, optimism, pessimism	9	SWOP-K9	The SWOP-K9 consists of two questionnaires that were combined and shortened [82]. These are the self-efficacy questionnaire [79] and the optimism questionnaire [80].<br>The resulting SWOP-K9 was compared with the original questionnaires in five different clinical samples with n=726 inpatients. Statistical validation revealed good to optimal scores [81].<br>The reliability of the SWOP-K9 is based on the testing of the individual questionnaires on self-efficacy with an internal consistency of α=0.86 (n=208) and on optimism with an internal consistency of α=0.76 (n=624). According to the creators, the questionnaire is valid for adults.	[79-82]
**WHO Quality of Life Instrument, Short Form**	physical health, psychological health, social relationships, environment	26	WHOQOL-BREF	The total sample (n=11830) included individuals from 23 countries. Of these, n=2308 people from Germany participated. Participants were adults recruited from a variety of inpatient and outpatient healthcare facilities as well as from the general population.<br>For the German-speaking sample, the internal consistency for the subdomains was as follows: physical health α=0.88, mental health α=0.83, social relationships α=0.76, and environment α=0.78.	[71]
**Work Ability Index**	Ability to work, physical and mental health, job require-ment and job satisfaction	7	WAI	The sample size (n=3968) comprises people employed in Germany.<br>The internal consistency of the scale was α= 0.75.	[70]

α = Cronbach's alpha

For peer review only - http://bmjopen.bmj.com/site/about/guidelines.xhtml

Probandeninformation und Einwilligungserklärung zum Forschungsvorhaben: Erprobung und Evaluierung einer psychosozialen Präventionsleistung im Rahmen des Projektes PE³PP - Fokusgruppeninterviews

Probandeninformation

Sehr geehrte Teilnehmerin, sehr geehrter Teilnehmer,

wir bitten Sie, an einer wissenschaftlichen Studie teilzunehmen. In dieser Probandeninformation finden Sie alles Wesentliche zu der Studie.

Bitte lesen Sie diese Information sorgfältig durch. Wir werden zusätzlich mit Ihnen über die Studie sprechen und Ihre Fragen beantworten.

Pro Fokusgruppe werden 7-10 Teilnehmer:innen eingeschlossen. Insgesamt sollen über den Projektverlauf zwölf Fokusgruppen durchgeführt werden, sodass sich eine Teilnehmendenzahl von insgesamt 84 bis zu 120 Teilnehmer:innen ergibt.

Diese Studie wird vom Lehrstuhl für Soziale Determinanten der Gesundheit, Fakultät für Sport- und Gesundheitswissenschaften an der Technischen Universität München geplant und durchgeführt.

Sie wird durch öffentliche Mittel (Bundesministerium für Arbeit und Soziales) gefördert.

Die Studie wurde der zuständigen Ethikkommission vorgelegt. Sie hat keine Einwände erhoben.

Die Teilnahme an der Studie ist freiwillig. Wenn Sie nicht teilnehmen wollen oder wenn Sie später Ihre Einwilligung widerrufen, werden Ihnen daraus keine Nachteile entstehen.

Wenn Sie jetzt oder auch später weitere Fragen zur Studie haben, können Sie uns gerne kontaktieren. Wenden Sie sich dazu an Johannes Stephan, Georg-Brauchle-Ring 60/62, 80992 München, johannes.stephan2@tum.de.

Warum wird diese Studie durchgeführt?

Die Präventionsleistung RV Fit Psychische Gesundheit, an der Sie teilnehmen, ist ein neues Angebot der Deutschen Rentenversicherung Mitteldeutschland. Da es ein neues Angebot ist, soll untersucht werden, wie die Präventionsleistung aus Sicht der Teilnehmenden, also aus Ihrer Sicht, umgesetzt werden sollte. Es sollen Erkenntnisse darüber gewonnen werden, welche Erwartungen und Erfahrungen Sie bezüglich der Präventionsleistung haben. Ein weiteres Ziel der Fokusgruppeninterviews ist es, Punkte, durch die die Präventionsleistung verbessert werden kann, zu identifizieren. Die Ergebnisse dieser Untersuchung sollen dann im konkreten dazu genutzt werden die Präventionsleistung stetig zu verbessern und auf Basis Ihrer Erwartungen und Erfahrungen die Präventionsleistung auch für zukünftige Teilnehmer:inenn von RV Fit Psychische Gesundheit optimal gestalten zu können.

Wie läuft die Studie ab?

Zu Beginn des vereinbarten Termins geben wir Ihnen die Möglichkeit, aufkommende Fragen und Anmerkungen hinsichtlich der Einwilligungserklärung zu stellen. Nachdem Sie informiert

For peer review only - http://bmjopen.bmj.com/site/about/guidelines.xhtml

wurden, bitten wir Sie die Einwilligungserklärung zu unterschreiben. Die unterschriebene Einwilligungserklärung können Sie dann entweder unmittelbar vor Ort, im Nachgang via E-Mail oder auch postalisch an die Forschenden übersenden. Die Fokusgruppen basieren auf einem wissenschaftlich konzipierten Leitfaden inklusive eines Erzählanreizes, der dazu dient den Austausch in der Gruppe zu initiieren. Die Fokusgruppe dauert zwischen 60 und 90 Minuten und wird aufgezeichnet. Die Fokusgruppe findet entweder in Präsenz vor Ort in den Kliniken oder online mittels einer Videokonferenz statt. Bei den Präsenzfokusgruppen erfolgt die Aufnahme mittels zweier im Raum verteilter Aufnahmegeräten. Die Präsenzfokusgruppen finden in entsprechenden passenden und geschützten Räumen in den Örtlichkeiten der Kliniken vor Ort statt. Die Onlinefokusgruppen finden über das Videokonferenztool Zoom statt. Die Tonaufnahme wird dabei anhand eines zusätzlich in das Zoom Meeting eingeloggten Computers sichergestellt, auf welchem über ein Aufnahmeprogramm aufgezeichnet wird.

Gibt es einen persönlichen Nutzen durch die Teilnahme an der Studie?

Durch die Teilnahme an den Fokusgruppeninterviews können Sie ihre Erfahrungen und Sichtweisen auf die absolvierte Präventionsleistung teilen und damit die Gestaltung und den Ablauf der Präventionsleistung mitgestalten. Ihre Perspektiven werden als wissenschaftliche fundiertes Feedback auch in das Projektteam rückgespiegelt.

Weiterhin ist es allerdings möglich, dass Sie durch Ihre Teilnahme keinen direkten Nutzen haben. Die Ergebnisse der Studie können jedoch in Zukunft anderen Menschen helfen.

Welche Risiken sind mit einer Teilnahme an der Studie verbunden?

Die Teilnahme an der Studie ist mit keinen Risiken verbunden.

Entstehen zusätzliche Kosten?

Durch die Teilnahme an der Studie entstehen Ihnen keine Kosten.

Was wird von mir erwartet?

Für die Teilnahme benötigen Sie nichts Weiteres. Alle notwendigen Materialien werden zur Verfügung gestellt. Die einzige Erwartung an Sie ist, dass Sie ihre Perspektive zu den verhandelten Themen kundtun.

Ist es möglich, im Verlauf aus der Studie auszuscheiden?

Ihre Teilnahme an dieser Studie ist freiwillig. Sie können jederzeit die Teilnahme beenden. Sie müssen dies nicht begründen. Es entstehen für Sie dadurch auch keine Nachteile.

Information zum Datenschutz

In dieser Studie ist der Lehrstuhl für Soziale Determinanten der Gesundheit für die Datenverarbeitung verantwortlich. Rechtsgrundlage für die Verarbeitung ist die persönliche

For peer review only - http://bmjopen.bmj.com/site/about/guidelines.xhtml

Einwilligung (Art. 6 Abs. 1a, Art. 9 Abs. 2a DSGVO). Die Daten werden zu jeder Zeit vertraulich behandelt.

Die Daten werden ausschließlich zum Zweck dieser oben beschriebenen Studie erhoben und nur in diesem Rahmen verwendet.

Die erfassten Daten umfassen auch personenbezogene Informationen, wie Ihre Stimme in der Tonbandaufnahme. Im nächsten Schritt wird die Tonbandaufnahme wortwörtlich niedergeschrieben, und danach wird die Aufnahme gelöscht. Alle Daten, durch die Sie unmittelbar identifiziert werden könnten, z.B. Ihr Name oder Ihr Geburtsdatum, werden durch einen Identifizierungscode ersetzt (pseudonymisiert). Damit ist es Unbefugten fast unmöglich, Sie zu identifizieren.

Die Daten werden an der Technische Universität München, Fakultät Sport- und Gesundheitswissenschaften, Lehrstuhl für Soziale Determinanten der Gesundheit gespeichert.

Wir bewahren die personenbezogenen Daten nur solange auf, wie dies für den oben genannten Zweck erforderlich ist. Die Daten werden spätestens nach Ablauf von 10 Jahre nach Abbruch oder Beendigung der Studie gelöscht.

Für die Studie führen wir eine Fokusgruppe mit Ihnen durch. Die Fokusgruppe vor Ort in Klinken wird mit Aufnahmegeräten aufgezeichnet. Die Fokusgruppen, welche während der Trainingsphase online via Videokonferenz durchgeführt werden, werden mittels eines zusätzlich eingeloggten Computers, auf welchem ein Aufnahmeprogramm läuft, aufgezeichnet. Die Tonaufnahme der Fokusgruppe wird zunächst an der Fakultät für Sport- und Gesundheitswissenschaften gespeichert. Das Gespräch wird innerhalb von sechs Monaten wortwörtlich aufgeschrieben (transkribiert). Die Tonaufnahmen werden dann gelöscht, sodass nur noch diese Abschrift des Gesprächs existiert. Alle Informationen, die dritten Personen Rückschluss auf Sie ermöglichen würden, werden bei der Abschrift so verändert, dass ein Rückschluss nicht mehr möglich ist. Der pseudonymisierte, aufgeschriebene Text wird für 10 Jahre in der Fakultät für Sport- und Gesundheitswissenschaften gespeichert und dann gelöscht. Wir übermitteln die personenbezogenen Daten weder an andere Einrichtungen in Deutschland, der EU, noch an ein Drittland außerhalb der EU oder an eine internationale Organisation.

Die Einwilligung zur Verarbeitung Ihrer Daten ist freiwillig. Sie können jederzeit die Einwilligung ohne Angabe von Gründen und ohne Nachteile für Sie widerrufen. Danach werden keine Daten mehr erhoben.

Sie können im Fall des Widerrufs die Löschung der erhobenen Daten verlangen. Die Daten können auch in anonymisierter Form weiterverwendet werden, wenn Sie dem zum Zeitpunkt Ihres Widerrufs zustimmen.

Sie haben das Recht, Auskunft über die Daten zu erhalten, auch in Form einer unentgeltlichen Kopie. Darüber hinaus können Sie die Berichtigung, Sperrung, Einschränkung der Verarbeitung oder Löschung sowie gegebenenfalls eine Übertragung der Daten verlangen. Die Rechtmäßigkeit der aufgrund der Einwilligung bis zum Widerruf erfolgten Verarbeitung wird hiervon nicht berührt.

Wenden Sie sich in diesen Fällen an:

For peer review only - http://bmjopen.bmj.com/site/about/guidelines.xhtml

Prof. Dr. Matthias Richter
Lehrstuhlinhaber - Soziale Determinanten der Gesundheit
Georg-Brauchle-Ring 60/62
80992 München
Tel.: +49 89 289 24190
richter.matthias@tum.de

Bei Rückfragen zur Datenverarbeitung und zur Einhaltung des Datenschutzes wenden Sie sich bitte an den Datenschutzbeauftragten:

Behördlicher Datenschutzbeauftragter der Technischen Universität München
Postanschrift: Arcisstr. 21, 80333 München
Telefon: 089/289-17052
E-Mail: beauftragter@datenschutz.tum.de

Sie haben ebenfalls das Recht, sich bei jeder Datenschutzaufsichtsbehörde zu beschweren. Eine Liste der Aufsichtsbehörden in Deutschland finden Sie unter: https://www.bfdi.bund.de/DE/Infothek/Anschriften_Links/anschriften_links-node.html

Die für Sie zuständige Aufsichtsbehörde erreichen Sie unter:

Bayerischer Landesbeauftragter für den Datenschutz
Postanschrift: Postfach 22 12 19, 80502 München
Hausanschrift: Wagmüllerstraße 18, 80538 München
E-Mail: poststelle@datenschutz-bayern.de

For peer review only

Probandeninformation und Einwilligungserklärung zum Forschungsvorhaben: Projekt zur Entwicklung, Erprobung und Evaluierung einer psychosozialen Präventionsleistung [PE³PP] - Fokussgruppeninterviews Einwilligungserklärung

Einwilligung zur Teilnahme

Ich wurde von ______________________________ über die Studie informiert. Ich habe die schriftliche Information und Einwilligungserklärung zu der oben genannten Studie erhalten und gelesen. Ich wurde ausführlich schriftlich und mündlich über den Zweck und den Verlauf der Studie, die Chancen und Risiken der Teilnahme und meine Rechte und Pflichten aufgeklärt. Ich hatte Gelegenheit Fragen zu stellen. Diese wurden zufriedenstellend und vollständig beantwortet. Zusätzlich zur schriftlichen Information wurden folgende Punkte besprochen:

____

____

Meine Einwilligung in die Teilnahme an der Studie ist freiwillig. Ich habe das Recht, meine Einwilligung jederzeit ohne Angabe von Gründen zu widerrufen, ohne dass mir dadurch Nachteile entstehen.

Ich willige hiermit in die Teilnahme an der oben genannten Studie ein.

____
Name der teilnehmenden Person in Druckbuchstaben

____
Ort, Datum Unterschrift der teilnehmenden Person

____
Name der aufklärenden Person in Druckbuchstaben

____
Ort, Datum Unterschrift der aufklärenden Person

Einwilligung zur Datenverarbeitung

Die Verarbeitung und Nutzung der persönlichen Daten für die oben genannte Studie erfolgt ausschließlich wie in der Information zur Studie beschrieben.

Ich willige hiermit in die beschriebene Verarbeitung meiner personenbezogenen Daten, insbesondere auch Gesundheitsdaten, ein.

____
Ort, Datum Unterschrift der teilnehmenden Person

____
Ort, Datum Unterschrift der aufklärenden Person

For peer review only - http://bmjopen.bmj.com/site/about/guidelines.xhtml

Reporting checklist for protocol of a clinical trial.

Based on the SPIRIT guidelines.

Instructions to authors

Complete this checklist by entering the page numbers from your manuscript where readers will find each of the items listed below.

Your article may not currently address all the items on the checklist. Please modify your text to include the missing information. If you are certain that an item does not apply, please write "n/a" and provide a short explanation.

Upload your completed checklist as an extra file when you submit to a journal.

In your methods section, say that you used the SPIRITreporting guidelines, and cite them as:

Chan A-W, Tetzlaff JM, Gøtzsche PC, Altman DG, Mann H, Berlin J, Dickersin K, Hróbjartsson A, Schulz KF, Parulekar WR, Krleža-Jerić K, Laupacis A, Moher D. SPIRIT 2013 Explanation and Elaboration: Guidance for protocols of clinical trials. BMJ. 2013;346:e7586

		Reporting Item	Page Number
**Administrative information**			
Title	#1	Descriptive title identifying the study design, population, interventions, and, if applicable, trial acronym	1

For peer review only

Trial registration	#2a	Trial identifier and registry name. If not yet registered, name of intended registry	2
Trial registration: data set	#2b	All items from the World Health Organization Trial Registration Data Set	n/a
Protocol version	#3	Date and version identifier	final
Funding	#4	Sources and types of financial, material, and other support	6; 16
Roles and responsibilities: contributorship	#5a	Names, affiliations, and roles of protocol contributors	16
Roles and responsibilities: sponsor contact information	#5b	Name and contact information for the trial sponsor	16
Roles and responsibilities: sponsor and funder	#5c	Role of study sponsor and funders, if any, in study design; collection, management, analysis, and interpretation of data; writing of the report; and the decision to submit the report for publication, including whether they will have ultimate authority over any of these activities	6-6
Roles and responsibilities: committees	#5d	Composition, roles, and responsibilities of the coordinating centre, steering committee, endpoint adjudication committee, data management team, and	n/a

For peer review only

		other individuals or groups overseeing the trial, if applicable (see Item 21a for data monitoring committee)	
**Introduction**			
Background and rationale	#6a	Description of research question and justification for undertaking the trial, including summary of relevant studies (published and unpublished) examining benefits and harms for each intervention	4-5; 9-11
Background and rationale: choice of comparators	#6b	Explanation for choice of comparators	n/a
Objectives	#7	Specific objectives or hypotheses	6; 8-11
Trial design	#8	Description of trial design including type of trial (eg, parallel group, crossover, factorial, single group), allocation ratio, and framework (eg, superiority, equivalence, non-inferiority, exploratory)	8
**Methods: Participants, interventions, and outcomes**			
Study setting	#9	Description of study settings (eg, community clinic, academic hospital) and list of countries where data will be collected. Reference to where list of study sites can be obtained	6-7

For peer review only

Eligibility criteria	#10	Inclusion and exclusion criteria for participants. If applicable, eligibility criteria for study centres and individuals who will perform the interventions (eg, surgeons, psychotherapists)	11-12
Interventions: description	#11a	Interventions for each group with sufficient detail to allow replication, including how and when they will be administered	6-8
Interventions: modifications	#11b	Criteria for discontinuing or modifying allocated interventions for a given trial participant (eg, drug dose change in response to harms, participant request, or improving / worsening disease)	n/a
Interventions: adherance	#11c	Strategies to improve adherence to intervention protocols, and any procedures for monitoring adherence (eg, drug tablet return; laboratory tests)	n/a
Interventions: concomitant care	#11d	Relevant concomitant care and interventions that are permitted or prohibited during the trial	n/a
Outcomes	#12	Primary, secondary, and other outcomes, including the specific measurement variable (eg, systolic blood pressure), analysis metric (eg, change from baseline, final value, time to event), method of aggregation (eg, median, proportion), and time point for each outcome. Explanation of the clinical relevance of chosen efficacy and harm outcomes is strongly recommended	13-14

For peer review only

Participant timeline	#13	Time schedule of enrolment, interventions (including any run-ins and washouts), assessments, and visits for participants. A schematic diagram is highly recommended (see Figure)	8-10
Sample size	#14	Estimated number of participants needed to achieve study objectives and how it was determined, including clinical and statistical assumptions supporting any sample size calculations	11-12
Recruitment	#15	Strategies for achieving adequate participant enrolment to reach target sample size	11-12
**Methods: Assignment of interventions (for controlled trials)**			
Allocation: sequence generation	#16a	Method of generating the allocation sequence (eg, computer-generated random numbers), and list of any factors for stratification. To reduce predictability of a random sequence, details of any planned restriction (eg, blocking) should be provided in a separate document that is unavailable to those who enrol participants or assign interventions	n/a
Allocation concealment mechanism	#16b	Mechanism of implementing the allocation sequence (eg, central telephone; sequentially numbered, opaque,	n/a

For peer review only

		sealed envelopes), describing any steps to conceal the sequence until interventions are assigned	
Allocation: implementation	#16c	Who will generate the allocation sequence, who will enrol participants, and who will assign participants to interventions	n/a
Blinding (masking)	#17a	Who will be blinded after assignment to interventions (eg, trial participants, care providers, outcome assessors, data analysts), and how	n/a
Blinding (masking): emergency unblinding	#17b	If blinded, circumstances under which unblinding is permissible, and procedure for revealing a participant's allocated intervention during the trial	n/a
**Methods: Data collection, management, and analysis**			
Data collection plan	#18a	Plans for assessment and collection of outcome, baseline, and other trial data, including any related processes to promote data quality (eg, duplicate measurements, training of assessors) and a description of study instruments (eg, questionnaires, laboratory tests) along with their reliability and validity, if known. Reference to where data collection forms can be found, if not in the protocol	12-14

For peer review only

Data collection plan: retention	#18b	Plans to promote participant retention and complete follow-up, including list of any outcome data to be collected for participants who discontinue or deviate from intervention protocols	n/a
Data management	#19	Plans for data entry, coding, security, and storage, including any related processes to promote data quality (eg, double data entry; range checks for data values). Reference to where details of data management procedures can be found, if not in the protocol	14
Statistics: outcomes	#20a	Statistical methods for analysing primary and secondary outcomes. Reference to where other details of the statistical analysis plan can be found, if not in the protocol	14-15
Statistics: additional analyses	#20b	Methods for any additional analyses (eg, subgroup and adjusted analyses)	n/a
Statistics: analysis population and missing data	#20c	Definition of analysis population relating to protocol non-adherence (eg, as randomised analysis), and any statistical methods to handle missing data (eg, multiple imputation)	n/a

Methods: Monitoring

Data monitoring: formal committee	#21a	Composition of data monitoring committee (DMC); summary of its role and reporting structure; statement of whether it is independent from the sponsor and competing interests; and reference to where further	14

For peer review only

		details about its charter can be found, if not in the protocol. Alternatively, an explanation of why a DMC is not needed	
Data monitoring: interim analysis	#21b	Description of any interim analyses and stopping guidelines, including who will have access to these interim results and make the final decision to terminate the trial	n/a
Harms	#22	Plans for collecting, assessing, reporting, and managing solicited and spontaneously reported adverse events and other unintended effects of trial interventions or trial conduct	n/a
Auditing	#23	Frequency and procedures for auditing trial conduct, if any, and whether the process will be independent from investigators and the sponsor	n/a
**Ethics and dissemination**			
Research ethics approval	#24	Plans for seeking research ethics committee / institutional review board (REC / IRB) approval	15
Protocol amendments	#25	Plans for communicating important protocol modifications (eg, changes to eligibility criteria, outcomes, analyses) to relevant parties (eg, investigators, REC / IRBs, trial participants, trial registries, journals, regulators)	n/a

For peer review only

Consent or assent	#26a	Who will obtain informed consent or assent from potential trial participants or authorised surrogates, and how (see Item 32)	15
Consent or assent: ancillary studies	#26b	Additional consent provisions for collection and use of participant data and biological specimens in ancillary studies, if applicable	n/a
Confidentiality	#27	How personal information about potential and enrolled participants will be collected, shared, and maintained in order to protect confidentiality before, during, and after the trial	15
Declaration of interests	#28	Financial and other competing interests for principal investigators for the overall trial and each study site	16
Data access	#29	Statement of who will have access to the final trial dataset, and disclosure of contractual agreements that limit such access for investigators	15
Ancillary and post trial care	#30	Provisions, if any, for ancillary and post-trial care, and for compensation to those who suffer harm from trial participation	n/a
Dissemination policy: trial results	#31a	Plans for investigators and sponsor to communicate trial results to participants, healthcare professionals, the public, and other relevant groups (eg, via publication, reporting in results databases, or other data sharing arrangements), including any publication restrictions	15

For peer review only

Dissemination policy: authorship	#31b	Authorship eligibility guidelines and any intended use of professional writers	15
Dissemination policy: reproducible research	#31c	Plans, if any, for granting public access to the full protocol, participant-level dataset, and statistical code	n/a

Appendices

Informed consent materials	#32	Model consent form and other related documentation given to participants and authorised surrogates	15
Biological specimens	#33	Plans for collection, laboratory evaluation, and storage of biological specimens for genetic or molecular analysis in the current trial and for future use in ancillary studies, if applicable	n/a

The SPIRIT Explanation and Elaboration paper is distributed under the terms of the Creative Commons Attribution License CC-BY-NC. This checklist was completed on 16. October 2023 using https://www.goodreports.org/, a tool made by the EQUATOR Network in collaboration with Penelope.ai

For peer review only
